# Genome-Wide Association Study of Growth Performance and Immune Response to Newcastle Disease Virus of Indigenous Chicken in Rwanda

**DOI:** 10.3389/fgene.2021.723980

**Published:** 2021-08-16

**Authors:** Richard Habimana, Kiplangat Ngeno, Tobias Otieno Okeno, Claire D’ andre Hirwa, Christian Keambou Tiambo, Nasser Kouadio Yao

**Affiliations:** ^1^College of Agriculture, Animal Science and Veterinary Medicine, University of Rwanda, Kigali, Rwanda; ^2^Animal Breeding and Genomics Group, Department of Animal Science, Egerton University, Egerton, Kenya; ^3^Rwanda Agricultural and Animal Resources Development Board, Kigali, Rwanda; ^4^Centre for Tropical Livestock Genetics and Health, International Livestock Research Institute, Nairobi, Kenya; ^5^Biosciences Eastern and Central Africa – International Livestock Research Institute (BecA-ILRI) Hub, Nairobi, Kenya

**Keywords:** genome-wide association studies, quantitative trait loci, putative gene, single nucleotide polymorphism, growth performance, indigenous chicken

## Abstract

A chicken genome has several regions with quantitative trait loci (QTLs). However, replication and confirmation of QTL effects are required particularly in African chicken populations. This study identified single nucleotide polymorphisms (SNPs) and putative genes responsible for body weight (BW) and antibody response (AbR) to Newcastle disease (ND) in Rwanda indigenous chicken (IC) using genome-wide association studies (GWAS). Multiple testing was corrected using chromosomal false detection rates of 5 and 10% for significant and suggestive thresholds, respectively. BioMart data mining and variant effect predictor tools were used to annotate SNPs and candidate genes, respectively. A total of four significant SNPs (rs74098018, rs13792572, rs314702374, and rs14123335) significantly (*p* ≤ 7.6E−5) associated with BW were identified on chromosomes (CHRs) 8, 11, and 19. In the vicinity of these SNPs, four genes such as pre-B-cell leukaemia homeobox 1 (PBX1), GPATCH1, MPHOSPH6, and MRM1 were identified. Four other significant SNPs (rs314787954, rs13623466, rs13910430, and rs737507850) all located on chromosome 1 were strongly (*p* ≤ 7.6E−5) associated with chicken antibody response to ND. The closest genes to these four SNPs were cell division cycle 16 (CDC16), zinc finger, BED-type containing 1 (ZBED1), myxovirus (influenza virus) resistance 1 (MX1), and growth factor receptor bound protein 2 (GRB2) related adaptor protein 2 (GRAP2). Besides, other SNPs and genes suggestively (*p* ≤ 1.5E−5) associated with BW and antibody response to ND were reported. This work offers a useful entry point for the discovery of causative genes accountable for essential QTLs regulating BW and antibody response to ND traits. Results provide auspicious genes and SNP-based markers that can be used in the improvement of growth performance and ND resistance in IC populations based on gene-based and/or marker-assisted breeding selection.

## Introduction

The genome-wide identification of genes linked to complex traits started in the 1990s (Zhang et al., 2012). The most suitable way to detect genetic variation for economically significant traits at the genome-wide level was to map quantitative trait loci (QTL; Soller et al., 2006). Earlier genomic studies have mostly used low-density marker assays like microsatellites (Goddard and Hayes, 2009). However, this approach does not provide novel information any longer (Zhang et al., 2012). Genome-wide association studies (GWAS) may now be undertaken on a larger scale thanks to the development of high-throughput genotyping tools and relevant statistical techniques (Sharmaa et al., 2015). Currently, GWAS is the most commonly used approach for searching a single variant and identifying functional complex traits genes (Berghof et al., 2018; Ji et al., 2019). Compared with previous QTL mapping approaches, GWAS is the most important in the detection of causal variants having simple effects. It is also powerful in delineating narrow genomic regions that have causal variants (MacKay et al., 2009; Sharmaa et al., 2015). GWAS does not assume that certain QTLs or genes are related to specific traits (Sharmaa et al., 2015), rather it gives the relationship between given traits and genetic markers (Berghof et al., 2018; Ji et al., 2019).

The use of GWAS in chickens has been to ascertain major loci related to economic traits (Liu et al., 2019; Saelao et al., 2019; Zhang et al., 2020). Most economic traits in animals display quantitative variation, which is regulated by many QTLs with comparatively small effects and altered by the environment (Tsudzuki et al., 2007). Growth performance traits are among the furthermost important economic traits in a poultry venture (Jin et al., 2015). Significant improvements in the study of growth performance traits in chicken have been realized, and many related genes and QTLs have been reported (Zhang et al., 2020). Over 1,500 QTLs, covering the majority of the chicken genome has been linked with growth performance traits (Hu et al., 2010; Sharmaa et al., 2015; Noorai et al., 2019). The majority of these reported QTLs are from crossbred chicken populations (Mebratie et al., 2019) and were discovered using microsatellites, markers with low map resolution, resulting in the detection of a few causative genes (Sharmaa et al., 2015).

Chicken has been threatened by various diseases (Jie and Liu, 2011) including Newcastle disease (ND; Kapczynski et al., 2013), a highly contagious viral disease of birds (El Sayed et al., 2016). The development of molecular and quantitative genetics assist in breeding for resistance in poultry (Walugembe et al., 2019). Currently, numerous efforts have been made globally to genetically improve disease resistance (Saelao et al., 2019). The immune capacity for specific diseases is a beneficial indicator of a good immunological response. This trait can be assessed and measured in live animals for indirect selection when breeding for resistance (Goddard and Hayes, 2009). Immunological characteristics such as antibody titres are heritable in poultry (Lamont et al., 2009; Lwelamira et al., 2009; Biscarini et al., 2010). This implies that there is a possibility of discovering loci and/or genes related to the immune and/or disease resistance traits. Selection for antibody response (AbR) is a suitable process for effectively improving resistance to diseases such as ND (Lillie et al., 2017). The development and distribution of disease-resistant chicken flocks represent a proactive approach for diseases control as compared to the current methods, which use drugs and vaccination (Dar et al., 2018). More than 10 QTLs associated with the antibody response to ND on chicken have been reported (Saelao et al., 2019; Walugembe et al., 2019).

Despite the existing QTL reports for BW and antibody response to ND in chicken, replication and confirmation of these QTL effects in IC in Rwanda have not been done. Growth performance and antibody response to ND of IC in Rwanda have been evaluated (Habimana et al., 2020b). This study examined the genetic architecture of BW and immune response to ND traits of IC in Rwanda. In this study, GWAS was performed to detect single nucleotide polymorphisms (SNPs) and candidate genes significantly related to growth and immune response to ND traits.

## Methodology

### Ethical Statement

All chicken manipulations were carried out as per the revised Animals Act 1986 with the approval of the ethical clearance committee of the College of Agriculture, Animal Sciences and Veterinary Medicine, University of Rwanda (Ref: 031/19/DRI September 2, 2019). The birds were humanely handled, and during the research, none of them was sacrificed.

### Experimental Birds

Indigenous chicken (IC) used in this study were kept under the same conditions on- station at the University of Rwanda in the Eastern region, Nyagatare district as explained in Habimana et al. (2020b).

### Phenotyping

The live body weights (BWs) of 185 ICs were weighed at the 20th week, and immune response to ND [mainly immunoglobulin Y (IgY)] titres recorded after 7 days from the second immunization (Table 1) as outlined in Habimana et al. (2020b).

**Table 1:** Means and SDs for 20-week body weight (BW) and antibody titres to Newcastle disease (ND) traits.

Traits	Samples	Mean	SD	Minimum	Maximum
Body weight (g)	185	1,435.15	549.62	706.35	3,000.50
Antibody (titre)	185	6,064.12	2,178.67	1,615	12,000

### Genotyping

Blood samples from 185 ICs were collected using an EDTA tube. Promega genomic DNA extraction kit was used to extract genomic DNA from blood. The concentration and quality of Genomic DNA were assessed using NanoDrop™ 2000 spectrophotometer (Thermo Scientific™ Nanodrop 2000) and gel electrophoresis (1% agarose) ensuring they met genotyping requirements. Genotyping-by-sequencing (GBS) approach (at the BecA-ILRI –Integrated Genotyping Service and support, Nairobi – Kenya) was used to obtain the raw reads (Elshire et al., 2011).

### Reads Alignment and SNP Calling

Raw reads were trimmed through sickle (Joshi and Fass, 2011) and aligned to Galgal6 (chicken reference genome) by the Burrows-Wheeler Alignment tool (BWA v0.7.17; Li et al., 2008). Picard package was used to remove duplicated reads. SNPs calling was done using SAMtools v1.3.1 (Li et al., 2009).

Resultant SNPs were subjected to the following filtering criteria in Plink v1.07 software (Purcell et al., 2007); minimum SNP quality of 20, 5% missing SNP genotypes, Hardy–Weinberg equilibrium (*p* < 10^−6^), call rate >95%, heterozygosity >0.4, and minor allele frequency >0.05. Genotype imputation was performed (Marchini and Howie, 2010) to increase the power of genome-wide analysis. Missing genotypes were imputed using the LD KNNi imputation method in Tassel 5.2.60 (Bradbury et al., 2007). For each chromosome, Tassel 5.2.60 estimated pairwise linkage disequilibrium (LD; Bradbury et al., 2007). Autosomal SNPs were pruned by using indep-pairwise parameters described by Wang et al. (2009), resulting in 65,945 independent SNP markers.

### Data Analysis

Multidimensional scaling (MDS) based on the centred identical-by-state (IBS) approach was used to test the population structure. Pairwise IBS distances were computed using independent SNPs. MDS components were got using the MDS-plot option based on the IBS matrix. Due to the internal population structure, the first MDS component was included as a covariate in the statistical model for assessing SNP effects on growth and antibody response attributes to account for sample stratification. Tassel 5.2.60 was used to construct a relative kinship matrix (K) using 65,945 independent SNP markers (Bradbury et al., 2007).

The analysis for GWAS was performed using Tassel 5.2.60 (Bradbury et al., 2007). SNPs significantly associated with body weight and antibody response to ND were identified using the following mixed linear model (MLM) as implemented in Tassel (Zhang et al., 2010).


y=X+Zu+e

where *y* is the vector of quantitative traits (20-week bodyweight and antibody response to Newcastle disease); *β*, vectors containing fixed effect (sex, gene pools, and SNPs) and covariates (population structure); *u*, a vector of random effect of the relative kinship matrix constructed by matrix simple matching coefficients based on the independent SNPs; *e*, a vector of random residuals; and *X* and *Z* are design matrices.

The family-wise error rate was controlled by using a Bonferroni correction. Based on the estimated number of independent SNP markers, the threshold value of *p* of the 5% Bonferroni genome-wide significance was computed. Therefore, the threshold value of *p* of the 5% Bonferroni genome-wide significance was 7.6E−5 (0.05/65,945). Similarly, the threshold value of *p* for the significance of suggestive linkage allowing one false-positive effect in a genome-wide test (Lander and Kruglyak, 1995) was calculated using the same approach as above and was 1.5E−5 (1/65,945). Relationship of normal theoretical quantiles of the probability distributions between expected (*x*-axis) and observed (*y*-axis) values of *p* from each, the, respectively, associated trait was shown by the Quantile-quantile (QQ) plots (Supplementary Datasheet 2). A comprehensive view of all values of *p* for each trait’s SNP markers was observed *via* the Manhattan plot. Manhattan and QQ plots were produced by the qqman package of the R software (Turner, 2018).

BioMart data-mining (Kinsella et al., 2011) and variant effect predictor (Dashti and Gamieldien, 2017) tools were used to annotate all significant SNPs found in GWAS, as well as genes situated 100 kb upstream and downstream of these SNPs, respectively. This was done in order to catalogue all of the genes found around the discovered SNPs, resulting in the compilation of gene lists for BW and antibody response to ND traits.

Database for Annotation, Visualization, and Integrated Discovery (DAVID; Dennis et al., 2003) was used to analyze putative genes for each trait. Gene ontology and functional annotation clustering analysis were performed to figure out what the mapped genes mean biologically. When enrichment score (ES) of the DAVID, an adjusted Fisher exact value of *p* is higher, it reflects more enriched clusters. When an ES is more than unity, the functional category is overrepresented.

## Results

Genome-wide association study using genotyping by sequencing identified SNPs and putative genes linked to growth performance and immune response to Newcastle disease virus in indigenous chicken in Rwanda.

### Population Structure of Indigenous Chicken in Rwanda

**Figure 1:**
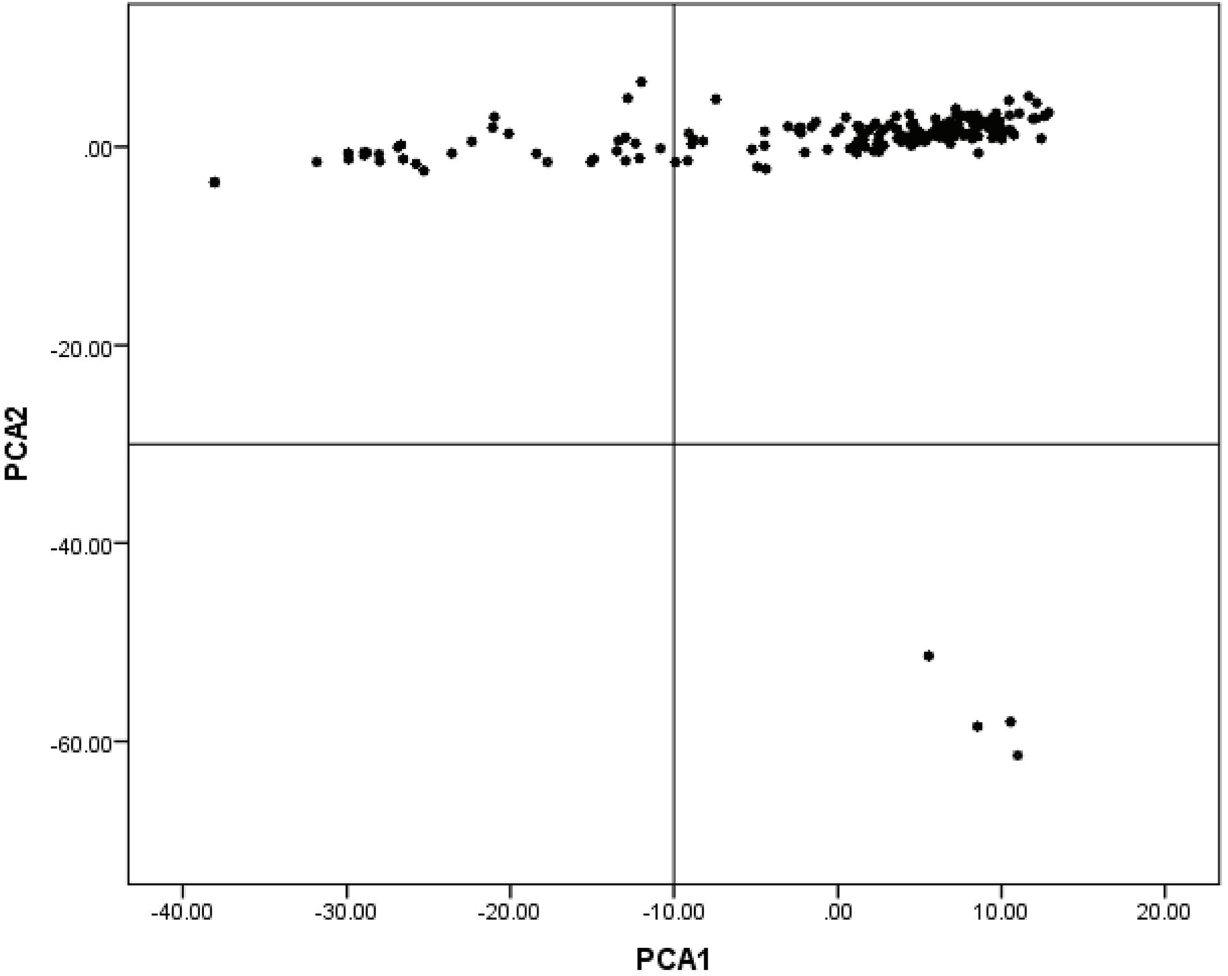
Population structure revealed by multidimensional scaling (MDS) analysis in indigenous chicken (IC) in Rwanda.

Multidimensional scaling analysis of 65,945 SNPs with the first two components revealed an internal population structure as explained by the variance among individuals’ population stratification (Figure 1).

### Genome-Wide Association Studies

#### Possible Causal Variant for Body Weight in Indigenous Chicken in Rwanda

**Figure 2:**
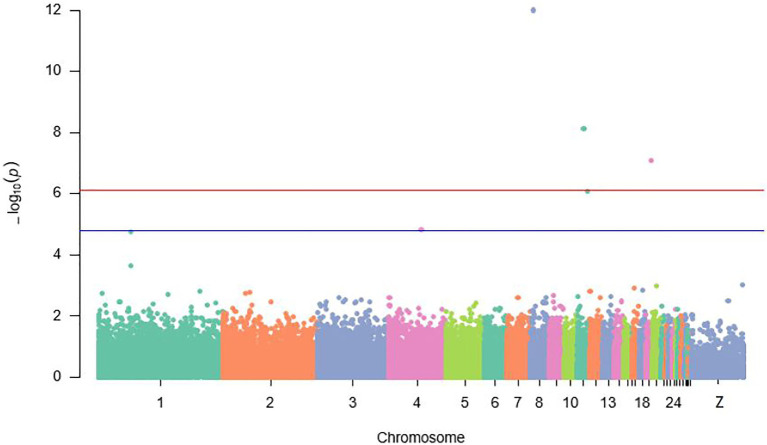
Manhattan plot displaying significant and suggestive single nucleotide polymorphisms (SNPs) associated with IC body weight {the red line designates a Bonferroni-adjusted genome-wide threshold [−log10 (*p*) ≥ 6.1] and the blue line indicates a suggestive genome-wide threshold [−log10 (*p*) ≥ 4.8]}.

Location and annotation of all significant and suggestive SNPs identified by GWAS are displayed in Figure 2; Tables 2 and 3. A total of four SNPs (rs74098018, rs13792572, rs314702374, and rs14123335) significantly (*p* ≤ 7.6E−5) associated with 20-week BW were detected on chromosomes 8, 11, and 19. Furthermore, rs318161016, a suggestive SNP (*p* ≤ 1.5E−5) associated also with BW was detected on chromosome 4 (Figure 2).

**Table 2:** Summary of significant SNPs and nearest genes detected at false discovery rate of 5% for body weight and antibody response to Newcastle disease in indigenous chicken in Rwanda.

Traits	SNP ID	CHR	SNP position (bp)	GWAS value of *p*	Allele	Candidate gene	Distance (bp)	Biological function
BW	rs740980181	8	5,672,840	1E−10	A	*PBX1*	Within	Positive regulation of cell proliferation and negative regulation of neuron differentiation
	rs13792572	11	10,145,725	7.6E−09	G	*GPATCH1*	7,341^D^	mRNA processing
	rs314702374	19	8,356,215	8E−08	G	*MPHOSPH6*	22,019^U^	Maturation of 5.8S rRNA
	rs14123335	11	15,831,341	8.4E−07	C	*MRM1*	54^U^	Enzyme-directed rRNA 2'-O-methylation
IR	rs314787954	1	137,692,275	4E−10	C	*CDC16*	Within	Protein K11-linked ubiquitination
	rs13623466	1	129,958,897	6.42E−08	T	*ZBED1*	7,631^U^	Regulation of transcription from RNA polymerase II promoter
	rs13910430	1	101,256,943	1E−07	T	*MX1*	29,169^U^	Innate immune response, organelle fission, and defense response to the virus
	rs737507850	1	50,383,558	1.55E−07	C	*GRAP2*	44,310^U^	Leukocyte-specific protein-tyrosine kinase signaling, innate and adaptive immune response

CHR, chromosome; BW, body weight; IR, immune response to Newcastle disease; SNP, Single nucleotide polymorphism; *PBX1*, *pre-B-cell leukaemia homeobox 1*; *GPATCH1*, *G-patch domain containing 1*; *MPHOSPH6*, *M-phase phosphoprotein 6*; *MRM1*, *mitochondrial rRNA methyltransferase 1*; *CDC16*, *cell division cycle 16*; *ZBED1*, *zinc finger, BED-type containing 1*; *MX1*, *myxovirus (influenza virus) resistance 1*; *GRAP2*, *GRB2 related adaptor protein 2*; superscript letters U and D indicate that the SNP is located upstream and downstream of the nearest gene, respectively.

**Table 3:** Summary of suggestive SNPs and nearest genes detected at false discovery rate of 10% for body weight and antibody response to Newcastle disease in indigenous chicken in Rwanda.

Traits	SNP ID	CHR	SNP position (bp)	GWAS value of *p*	Allele	Candidate genes	Distance (bp)	Biological function
BW	rs318161016	4	51,805,394	0.00001535	A	*FGF2*	8,033^U^	Growth factor-dependent regulation of skeletal muscle satellite cell proliferation
IR	rs735333650	17	8,458,226	0.000000939	T	*UBAC1*	Within	Regulation of cell survival and proteasomal degradation
	rs15900019	8	4,304,485	0.00000138	A	*TEDC1*	Within	Duplication and assembly of centrioles and basal bodies
	rs1060144701	26	2,497,963	0.00000145	A	*RASSF5*	Within	Negative regulation of cell proliferation and positive regulation of protein ubiquitination
	rs14118744	19	3,336,281	0.00000184	G	*IFT22*	Within	Small GTPase mediated signal transduction
	rs736576816	2	61,140,781	0.00000191	G	*JARDI2*	Within	Liver, spleen, and thymus development, stem cell differentiation, and positive regulation of histone H3-K9 methylation
	rs741342879	13	8,272,097	6.2874E−06	T	*GABRB2*	67,439^D^	Ion transport and negative regulation of neuron apoptotic process
	rs736427856	2	119,443,614	0.00000729	T	*ZFHX4*	377^D^	Zinc ion binding and sequence-specific DNA binding
	rs1060031521	2	93,149,588	0.0000079	T	*ADCYAP1*	89,139^U^	Positive regulation of the cAMP biosynthetic process, drinking behavior, and positive regulation of hormone secretion
	rs739117494	13	16,352,162	0.00000832	T	*PCBD2*	Within	Tetrahydrobiopterin biosynthetic process, positive regulation of transcription, DNA-templated, protein homotetramerisation, and protein heterooligomerisation
	rs740392770	4	51,805,394	0.00001201	T	*ALB*	7,988^U^	Negative regulation of apoptotic process and cellular response to starvation, vitamin A, and virus

CHR, chromosome; BW, body weight; IR, immune response; SNP, single nucleotide polymorphism; *FGF2*, *Fibroblast growth factor 2*; *UBAC1*, *ubiquitin-associated domain containing 1*; *TEDC1*, *tubulin epsilon and delta complex 1*; *RASSF5*, *Ras association domain family member 5*; *IFT22*, *intraflagellar transport 22*; *JARDI2*, *jumonji and AT-rich interaction domain containing 2*; *GABRB2*, *gamma-aminobutyric acid (GABA) A receptor, beta 2*; *ZFHX4*, *zinc finger homeobox 4*; *ADCYAP1*, *adenylate cyclase-activating polypeptide 1*; *PCBD2*, *pterin-4 alpha-carbinolamine dehydratase 2*; *ALB*, *albumin*; superscript letters U and D indicate that the SNP is located upstream and downstream of the nearest gene, respectively.

Lists of candidate genes situated 100 kb downstream and upstream of the significant SNPs for BW at week 20 are summarized in Table 2 and Supplementary Table S1. Among all the mapped genes in the candidate genomic regions, only four known genes namely *pre-B-cell leukaemia homeobox 1* (*PBX1*), *G-patch domain containing 1* (*GPATCH1*), *M-phase phosphoprotein 6* (*MPHOSPH6*), and *mitochondrial rRNA methyltransferase 1* (*MRM1*) were the closest to the significant SNPs (Table 2). One gene called *Fibroblast growth factor 2* (*FGF2*) was reported in the vicinity of a suggestive SNP (Table 2).

Functional annotation clustering analysis of all mapped genes for BW exposed the presence of enriched gene groups related to transcription factor activity, sequence-specific DNA binding, arylamine *N*-acetyltransferase activity, and growth factor activity (Supplementary Table S2). The biological functions of all genes neighboring the significant and suggestive SNPs for BW were identified and are presented in the Supplementary Table S3.

#### Possible Causal Variant for Antibody Response to Newcastle Disease in Indigenous Chicken in Rwanda

**Figure 3:**
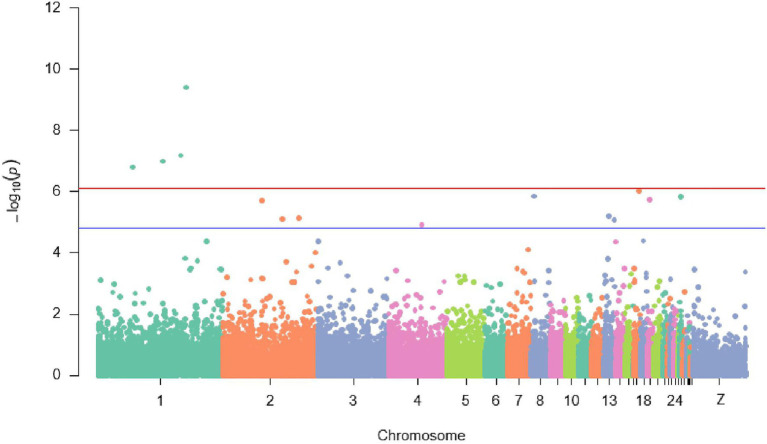
Manhattan plot displaying significant and suggestive SNPs associated with antibody response to Newcastle disease {the red line designates a Bonferroni-adjusted genome-wide threshold [−log10 (*p*) ≥ 6.1] and the blue line indicates a suggestive genome}.

Overall view of values of *p* for all the SNPs contributing to the antibody response to ND showed that a genomic region on chromosome 1 with four SNPs (rs314787954, rs13623466, rs13910430, and rs737507850) was strongly (*p* ≤ 7.6E−5) associated with the IC antibody response to ND after 7 days from the second immunization. Additionally, 10 suggestive SNPs (*p* ≤ 1.5E−5) detected on chromosomes 2, 4, 8, 13, 17, 19, and 26 influenced also antibody response to ND in IC in Rwanda (Figure 3).

All genes located at 100 kb upstream and downstream of the four significant and suggestive SNPs were identified for antibody response to ND (Supplementary Table S1). Among the known genes, four including *cell division cycle 16* (*CDC16*), *zinc finger, BED-type containing 1* (*ZBED1*), *myxovirus (influenza virus) resistance 1* (*MX1*), and *growth factor receptor bound protein 2 (GRB2) related adaptor protein 2* (*GRAP2*) were the closest to the significant SNPs (Table 2). In addition, a total of 10 known genes namely *ubiquitin-associated domain containing 1* (*UBAC1*), *tubulin epsilon and delta complex 1* (*TEDC1*), *Ras association domain family member 5* (*RASSF5*), *intraflagellar transport 22* (*IFT22*), *jumonji and AT-rich interaction domain containing 2* (*JARDI2*), *gamma-aminobutyric acid A receptor, beta 2* (*GABRB2*), *zinc finger homeobox 4* (*ZFHX4*), *adenylate cyclase-activating polypeptide 1* (*ADCYAP1*), *pterin-4 alpha-carbinolamine dehydratase 2* (*PCBD2*), and *albumin* (*ALB*) were mapped in the neighborhood of the identified suggestive SNPs (Table 3). Biological functions of these genes mapped nearby significant and suggestive SNPs for antibody response (Ab) response to ND are presented in Supplementary Table S3.

Functional annotation clustering analysis exposed the presence of enriched gene clusters associated with regulation of transcription, DNA-templated, metal-ions binding and transport, ATP binding, and cytokine activity (Supplementary Table S2).

## Discussion

Genome-wide association study is a prevailing tool for genetic analysis of essential traits in domestic animals. In this study, a GWAS experiment was performed to investigate the genetic basis of the BW and Ab response to ND among IC populations in Rwanda. A supposition made in GWAS is that it is possible to identify significant associations since SNPs are in LD with causal mutations for the traits of interest (Zhang et al., 2012).

### Population Structure of Indigenous Chicken in Rwanda

The structured population witnessed in this study is consistent with findings in the previous report on the IC population in Rwanda (Habimana et al., 2020a). This population stratification could, however, confound GWAS. Nevertheless, the MLM method used in this study corrected for the stratification and removed confounding effects (Price et al., 2010) and markedly reduced the number of false-positive associations (Sharmaa et al., 2015).

### Genome-Wide Association Studies

#### Possible Causal Variant for Body Weight in Indigenous Chicken in Rwanda

Bodyweight is under complex genetic control (Zhao et al., 2012). Detection of the molecular growth mechanism would contribute to a more efficient selection for growth in chicken (Deeb and Lamont, 2002). In this study, the GWAS resulted in the identification of four significant and one suggestive SNPs associated with BW. Of these four leading SNPs, one was detected on chromosome 8, two on chromosome 11, and one on chromosome 19. However, the strongest association signal was revealed on chromosome 8. This study confirmed the existing candidate genomic regions for BW on chromosomes 8 (Pértille et al., 2017) and 11 (Jennen et al., 2004; Jacobsson et al., 2005; McElroy et al., 2006). However, the region on chromosome 19, which is associated with BW can be taken as a novel candidate genomic region controlling BW in IC, since QTL or SNP was never reported in this genomic region.

In the present study, a suggestive QTL for 20-week BW was discovered on chromosome 4. This suggestive QTL corroborated previous studies, which revealed QTLs for BW on the same chromosome (Tsudzuki et al., 2007; Sharmaa et al., 2015; Pértille et al., 2017). Our study also confirmed a study by Johansson et al. (2010), which reported that a region on chromosome 4 was recounted to be subjected to intense selection in several chicken lines with different selection on BW for up to 50 generations. This phenomenon could be attributed to the fact that one QTL on chromosome 4 harbors genes with vast effects on developmental increases in BW (Ankra-Badu et al., 2010). Chromosome 4 could harbor an important genomic region for further analysis as the different selection responses in chicken lines are triggered by the genomic region covering one or more genes with genetic background-dependent effects. In previous studies, apart from chromosomes 4, 8, 11,16, 19, 20, 22, and 25, the remaining chromosomes including Z were reported to harbor QTLs affecting BW in chicken at different ages (Gu et al., 2011; Rikimaru et al., 2011; Xie et al., 2012). However, major QTLs are commonly mapped on chromosome 1 (Liu et al., 2007; Tsudzuki et al., 2007; Uemoto et al., 2009).

The differences in QTL affecting BW could be attributed to several factors such as breed types and generations (Gu et al., 2011; Xie et al., 2012) and experimental population used (McElroy et al., 2006). The magnitude of QTL affecting BW may be population-dependent and the frequency of alleles in any population depends on that population’s adaptive environment. For instance, the growth dynamics of local chicken are different from layers, broilers, and crossbreed populations (Ankra-Badu et al., 2010), and grandparental breeds used for the construction of the inter-crossed lines (Tsudzuki et al., 2007). Age differences also account for the differences in QTLs (Uemoto et al., 2009; Pértille et al., 2017). This study indicates the existence of several genes involved in the growth and development of chicken at various stages of life. In addition, age-specific QTLs have been found to control BW on chromosomes 1 and 4 in earlier studies; QTL controlling BW at initial stages was identified on chromosome 1 (Liu et al., 2007; Uemoto et al., 2009). This confirms the presence of major QTLs on chromosome 1 modulating early development (Rikimaru et al., 2011). Contrarily, QTLs for BW at later ages like in this study were uncovered on chromosome 4 (Rikimaru et al., 2011; Pértille et al., 2017) with few exceptional cases reported by Wardȩcka et al. (2002) and Kerje et al. (2003). Consideration should, therefore, be given to the age-specificity of the QTLs. Consequently, QTL studies on BW, each week from hatching to adult would be needed for the identification of age-specific QTLs. The discrepancy in QTLs in previous studies could be also a result of the mapping approach used. Earlier genomic studies have mostly used low-density microsatellites markers (Tsudzuki et al., 2007). Currently, GWAS employs, however, SNPs as potential markers that are dispersed throughout the whole genome at a higher density (Zhang et al., 2012). Finally, the choice of the statistical model could explain the inconsistent results (McElroy et al., 2006). Some studies used a general linear model (GLM; Wang et al., 2015; Zhang et al., 2015a), and others including this study, used a MLM (Zhang et al., 2013; Mebratie et al., 2019). The study by Mebratie et al. (2019) emphasized that using GLM and MLM in GWAS, does not essentially give the same results even without the presence of a robust population structure in the data. Kennedy et al. (1992) reported that in the presence of relations between animals, the use of GLM analysis results in an inflated *F*-test. This leads to an excess of spurious significant effects of QTLs, even if there is no effect or bias in effect estimation. The MLM approaches have, however, demonstrated worthwhile in controlling population structure and relatedness within GWAS. This analysis method offers the utmost power to discover QTLs while controlling at the preferred level for false-positive rate or false discovery rate and providing the greatest accurate estimates of QTL effect (Zhang et al., 2010; Ekine et al., 2014).

Regions on chromosomes 8, 11, and 19 seemed to be promising genomic regions for putative genes controlling indigenous chicken BW. Within these genomic regions, five candidate genes namely *PBX1*, *GPATCH1*, *MPHOSPH6*, *MRM1*, and *FGF2* were discovered. *PBX1* gene, localized in the nucleus (Berthelsen et al., 1999; Mercader et al., 1999), has been known as a homeodomain transcription factor that can form heterodimers with homeodomain proteins. These proteins are encoded by the *homeotic selector* (*Hox*) gene complexes and rise their DNA-binding affinity and specificity. McWhirter et al. (1997) suggested that interactions between *PBX1* gene and homeodomain proteins were required for Hox proteins to control downstream target genes that in turn regulate growth, differentiation, and morphogenesis during development. The expression pattern of this gene suggests its importance in early nervous system development. This gene was initially identified for its role in the translocation of the chromosome which happens in pre-B acute lymphoblastic leukaemia (Kamps et al., 1990; Nourse et al., 1990). Besides, Charboneau et al. (2006) revealed the involvement of *PBX1* in pro-angiogenic Hox DNA binding and transcriptional activity in endothelial cells. A study in the rodent fibroblast revealed that *PBX1* encodes a new different member of the fibroblast growth factor (FGF) family (McWhirter et al., 1997). The same study unveiled that *PBX1* homeodomain was required for the induction of FGF. This confirms the presence of *FGF2* among the identified candidate gene in this study. Therefore, we may hypothesize that *PBX1* has a significant effect on chicken growth. *FGF2* is known for its role in regulating skeletal muscle satellite cell proliferation. Also, *FGF2* is famous to be related to chicken growth (Xue et al., 2017). Another study confirms this by revealing two genes of the family of FGF namely growth factor binding protein 1 (*FGFBP1*) and 2 (*FGFBP2*) in modulating chicken growth (Ankra-Badu et al., 2010). *GPATCH1* gene is involved in mRNA processing. Besides its role in RNA transport inside the cells, the *G-patch domain* has been linked with other RNA processing functions (Yiu et al., 2004). This gene was identified and was believed to be associated with biogenesis and the developmental process in Goat (Brito et al., 2017). Moreover, it has been characterized as a novel cholesterol metabolism regulator, reducing cholesterol synthesis, and increasing the concentration of low-density lipoprotein uptake in chicken (Zhang et al., 2019). The role of the *GPATCH1* gene in modulating BW in chicken has not yet been elucidated. *MPHOSPH6* gene is involved in maturation of 5.8S rRNA and a novel target in Cytokine-mediated modulation of the hepatic miRNome (Kirchmeyer et al., 2018). Besides, *MPHOSPH6* regulates the shrimp cell cycle and development of ovary in black tiger (Zhou et al., 2013). However, its role in BW regulation in chicken has not been yet discussed. *MRM1* gene is an enzyme-directed rRNA 2'-O-methylation (Lee et al., 2013; Lee and Bogenhagen, 2014), but its function in controlling BW has not been established.

In the present study, some QTLs for 20-week BW are overlapping those already found in previous studies and others have never been reported. This study, therefore, postulates that these genomic regions may play a key role in the fundamental molecular mechanisms that are responsible for BW. These genomic regions are hence separate in many various chicken populations, confirming the difference in BW revealed in IC gene pools in Rwanda (Habimana et al., 2020a). Besides, this study offers a preliminary assumption that genes uncovered in these genomic regions could be promising candidate genes for 20-week BW in IC populations in Rwanda. Extra confirmation experimentations, however, suggest *PBX1*, *GPATCH1*, *MPHOSPH6*, and *MRM1* be novel targets for 20-week BW. For additional validation of the importance of these genes, studies including their silencing and overexpression need to be conducted both *in-vivo* and *in-vitro*. Since growth performance is the utmost crucial trait of selection, getting profound insights into the growth molecular mechanism would result in a more efficient selection for growth performance in chickens.

#### Possible Causal Variant for Antibody Response to Newcastle Disease in Indigenous Chicken in Rwanda

Host antibody response to viruses is a composite process (Fischer et al., 2013). Immune response to ND could be considered as a quantitative trait under polygenic control, but having few QTLs (Biscarini et al., 2010; Luo et al., 2013; Saelao et al., 2019). In this present study, all four significant SNPs strongly (*p* ≤ 7.6E−5) associated with antibody (Ab) response to ND were detected on chromosome 1 (50,383,558–137,692,275 bp). This study confirmed the findings by Luo et al. (2013) and Saelao et al. (2019) who recounted chromosome 1 to harbor genomic region associated with Ab response to ND, hence strongly suggests the presence of an important regulatory region for ND control in this region. Also, this genomic region on chromosome 1 overlays the QTLs for the Ab response to sheep red blood cells (Dorshorst et al., 2011). Therefore, this region could have great importance for chicken immune response and, probably, disease resistance in general.

Conversely, results from this study differed from those previously reported (Yonash et al., 2001; Biscarini et al., 2010; Wang et al., 2015). Yonash et al. (2001) reported that QTLs for the Ab response to ND in broiler chicken were located on chromosomes 2 and 18. Biscarini et al. (2010) reported 13 QTLs related to Ab response to ND on chromosomes 3, 4, 5, 9, 13, 16, and 22, and Z. Wang et al. (2015) revealed six QTLs affecting Ab response to ND on chromosomes 2, 4, and Z, whereas Jacob et al. (2000) discovered on chromosome 16, major histocompatibility complex (MHC) which is associated with chicken immunity (Zhou and Lamont, 2003; Zhang et al., 2015b). These inconsistent results could have happened for numerous reasons; comprising a dose of ND vaccine applied, time post-vaccination, markers used, choice of statistical models, the genetic composition of the experimental populations, and limited power of most QTLs mapping studies (McElroy et al., 2006; Ioannidis et al., 2009; Saelao et al., 2019).

The current study used IC resource populations, while other previous studies were based on broiler and layer populations (Yonash et al., 2001; Biscarini et al., 2010). The present work used an MLM while the previous studies performed GWAS through a GLM (Wang et al., 2015). The majority of the previous studies used microsatellite markers whereas this study performed GWAS using SNPs. In addition, the nature of the Ab response targeted could result in different QTLs (Saelao et al., 2019). Previous studies focused on the primary Ab response to ND virus, while the present study scrutinized the secondary Ab response to ND virus. In the primary Ab response, the main class of Ab produced is immunoglobulin M (IgM) whereas in the secondary Ab response is IgY (Sarker et al., 1999); thus, the ranking of chicken on Ab response to the primary vaccination could be different to that after the second one leading to the detection of different QTLs (Biscarini et al., 2010). The QTLs for the Ab response to ND in this study could reflect the aptitude of the memory cell pool to respond to ND.

In addition to the significant SNPs, 10 suggestive SNPs (*p* ≤ 1.5E−5) were detected on chromosomes 2, 4, 8, 13, 17, 19, and 26. However, apart from chromosomes 2, 4, and 13 which were reported earlier (Rowland et al., 2018; Saelao et al., 2019; Walugembe et al., 2019), there were no other earlier studies on QTLs modulating ND antibody response on chromosomes 8, 17, 19, and 26. Contrarily, Luo et al. (2013) uncovered a candidate QTL for the ND antibody response on chromosome 12. This could be attributed to the different techniques used in the current study to identify SNPs, which might have permitted the detection of novel SNPs. Surprisingly and consistently, one QTL currently identified on chromosome 4 was also associated with BW at 20 weeks of age. This could be explained by the pleiotropic mechanisms of the QTL region on that chromosome.

Some QTLs for ND antibody response reported in the current study confirmed those found in earlier studies, and other ones have never been reported. This study, therefore, hypothesizes that the reported genomic regions could contribute to the important molecular mechanisms responsible for the total effective host AbR. These genomic regions segregate in several diverse chicken populations, confirming the difference in Ab response to ND existing in IC gene pools in Rwanda (Habimana et al., 2020a). However, further investigations preferably with independent and large populations (Spelman and Bovenhuis, 1998; Marklund et al., 1999), are needed to validate the findings from this study.

Disease resistant genes encode antibodies, microRNA, and other materials helping the host to repel the harm caused by pathogens (Dar et al., 2018). So far, lots of anti-disease genes (*MH*, *NRAMP1*, *IFN*, *MX*, *ANTI-ALV*, *ZYXIN*, *TVB*, *CD1B*, *CD1CB*, *ROBO1*, *ROBO2*, *CHMP2B*, *MHC*, *SEMA5A*, and *TGFBR2*) have been revealed with the development of several molecular technologies and assays in chickens (Zhang et al., 2015b; Deist et al., 2017; Lillie et al., 2017). Other genes related to poultry immunity (*CAMK1d*, *CCDC3 TIRAP*, *ETS1*, and *KIRREL3*) are still under a validation process (Saelao et al., 2019). However, the accurate mechanism of disease resistance is not entirely clear (Jie and Liu, 2011), and only a few causal genes have been identified due to low map resolution (Luo et al., 2013). Many more genes associated with disease resistance (Jie and Liu, 2011) could be revealed with a high map resolution.

This study revealed, however, 14 putative genes (*CDC16*, *ZBED1*, *MX1*, *GRAP2*, *UBAC1*, *TEDC1*, *RASSF5*, *IFT22*, *JARDI2*, *GABRB2*, *ADCYAP1*, *PCBD2*, *ZFHX4*, and *ALB*) associated with Ab response to ND in IC populations in Rwanda. The gene ontology annotation advises that all these genes contribute to the regulation of transcription, binding, transport, cytokine activity, and immune responses. Among these genes, four were near the significant SNPs (*CDC16*, *ZBED1*, *MX1*, and *GRAP2*). These genes might be used as putative genes to be further explored to determine associations with Ab response to ND in IC populations in Rwanda.

The *CDC16* gene identified in this stud is yet to be characterized in chicken immunity. In mammals, the *CDC16* gene is, however, necessary for the normal coupling of DNA replication to mitosis and might act downstream of *CDC28* to negatively control DNA replication (Heichman and Roberts, 1998). This gene plays a significant role in cellular functions in mammals, and defects of which are closely associated with various disease processes (Shi et al., 2018). Besides, Paglialunga et al. (2017) established a vital role for *CDC16* in maintaining *in vivo* β-cell mass. This gene may have a similar function in chickens and could be a promising gene for Ab response for ND in chickens. *ZBED1* gene also known as DNA replication-related element-binding factor uncovered in this study, was initially identified as a transcription factor in *Drosophila* (Hirose et al., 1996). It binds to box A and positively regulates genes involved in DNA replication and cell proliferation, such as the proliferating cell nuclear antigen and DNA polymerase (Matsukage et al., 2008). Later on, a study found that *ZBED1* played a crucial role in promoting proliferation and decreases the chemosensitivity of gastro cancer cells (Jiang et al., 2018). Still, no information concerning its function and key mechanisms in chicken immunity is reported. *GRAP2* gene also known as *GADS*, *GRAP-2*, *GRB2L*, *GRBLG*, *GRID*, *GRPL*, *GrbX*, *Grf40*, *Mona*, and *P38* has been identified in the current study. The function of this gene has not yet been established in chicken but it has been shown in humans that the *GRAP2* gene is involved in leukocyte-specific protein-tyrosine kinase signaling and immune response by stimulating T cells (Dufner et al., 2015; Breuning and Brown, 2017). This role makes this gene a promising gene for Ab response for ND in Chicken. This study confirmed the previous results which revealed that *MX1* gene was associated with Ab response to ND in chicken (Mpenda et al., 2020). Apart from that, the *MX1* gene is associated with resistance to avian influenza (AI) and infectious bursal disease virus (IBDV; Yin et al., 2010; Ewald et al., 2011; Jie and Liu, 2011). Thus, this is an indication that the *MX* gene is involved in chicken immunity.

Ten genes (*UBAC1*, *TEDC1*, *RASSF5*, *IFT22*, *JARDI2*, *GABRB2*, *ADCYAP1*, *PCBD2*, *ZFHX4*, and *ALB*) were found to be associated with suggestively correlated SNPs for Ab response to ND in this study. All these candidate genes for Ab response to ND have not been heretofore directly associated with immune response in poultry (Adhikari and Davie, 2018; Elbeltagy et al., 2019). Some of these genes are new genes in the chicken and have mostly been discussed in other species (Zhou et al., 2014; Fleming et al., 2016; Zhuang et al., 2020). Further examination is therefore required to prove these novel genes as putative genes for Ab response to ND in chicken. Kompetitive Allele Specific Polymerase Chain Reaction (KASP)-based markers (Semagn et al., 2014; Islam and Blair, 2018) could be developed using the SNPs significantly associated with Ab response to ND and validated before being used to screen targeted chicken breeds to establish the involvement of these genes in chicken immunity to ND. Another approach will be to develop an RNA-guided cas9 nuclease from the microbial clustered regularly interspaced short palindromic repeats (CRISPR-CAS9) protocol (Ran et al., 2013; Doudna and Charpentier, 2014) for chicken with the ultimate aim of silencing these genes to ascertain their role in the chicken immunity to ND.

## Conclusion

Genomic regions that putatively control body weight and antibody response to Newcastle disease in an indigenous chicken were identified in the current study. This information provides insights on the genetic control of these traits and makes available genetic markers that could be useful for selective breeding programs to improve growth performance and ND resistance in IC. Few of the genomic regions overlapped with hitherto reported QTL regions provide evidence for confirmation of these QTLs and their corresponding effects. In addition, the variants and genes uncovered for the first time in this study, merit further scrutiny to understand the fundamental molecular mechanisms before practical application.

## Data Availability Statement

The original contributions presented in the study are included in the article/Supplementary Material, further inquiries can be directed to the corresponding author.

## Ethics Statement

The animal study was reviewed and approved by The Research Screening and Ethical Clearance Committee of the College of Agriculture, Animal Sciences, and Veterinary Medicine, University of Rwanda. Written informed consent was obtained from the owners for the participation of their animals in this study.

## Author Contributions

RH and KN: conception of the work and data analysis. CH: contributed to the data acquisition. RH, KN, and NY: results interpretation. RH: drafting the article. KN, CK, NY, and TO: critical revision of the article and final approval of the version to be published. All authors contributed to the article and approved the submitted version.

## Conflict of Interest

The authors declare that the research was conducted in the absence of any commercial or financial relationships that could be construed as a potential conflict of interest.

## Publisher’s Note

All claims expressed in this article are solely those of the authors and do not necessarily represent those of their affiliated organizations, or those of the publisher, the editors and the reviewers. Any product that may be evaluated in this article, or claim that may be made by its manufacturer, is not guaranteed or endorsed by the publisher.
