# Supplementary material for: Genome-Wide Association Study of Growth Performance and Immune Response to Newcastle Disease Virus of Indigenous Chicken in Rwanda
Source: Front Genet. 2021 Aug 16;12:723980. doi: 10.3389/fgene.2021.723980 (PMC8570395; doi:10.3389/fgene.2021.723980)
Supplement: Supplementary file 1 [file Data_Sheet_1.zip › Suppl. Table 1.DOCX]

**S1 Table. Catalogue of all genes located 100 kb upstream and downstream of the significant SNPs for body weight and antibody response to Newcastle disease in indigenous chicken in Rwanda**

| **SNP ID** | **Location** | **Allele** | **Consequence** | **SYMBOL** | **Gene** | **BIOTYPE** | **DISTANCE (Bp)** | **ENSP** |
| --- | --- | --- | --- | --- | --- | --- | --- | --- |
| **Antibody Response** |  |  |  |  |  |  |  |  |
| **Significant SNPs** |  |  |  |  |  |  |  |  |
| rs314787954 | 1:138290589-138290589 | C | intron_variant | CDC16 | ENSGALG00000016816 | protein_coding | Within | ENSGALP00000027097 |
| rs314787954 | 1:138290589-138290589 | C | upstream_gene_variant | - | ENSGALG00000016815 | protein_coding | 7876 | ENSGALP00000073904 |
| rs314787954 | 1:138290589-138290589 | C | upstream_gene_variant | - | ENSGALG00000016815 | protein_coding | 9159 | ENSGALP00000027096 |
| rs314787954 | 1:138290589-138290589 | C | upstream_gene_variant | - | ENSGALG00000051908 | lncRNA | 13896 | - |
| rs314787954 | 1:138290589-138290589 | C | upstream_gene_variant | - | ENSGALG00000050442 | protein_coding | 26351 | ENSGALP00000065694 |
| rs314787954 | 1:138290589-138290589 | C | upstream_gene_variant | CHAMP1 | ENSGALG00000016813 | protein_coding | 39460 | ENSGALP00000027093 |
| rs314787954 | 1:138290589-138290589 | C | downstream_gene_variant | - | ENSGALG00000055129 | lncRNA | 41846 | - |
| rs314787954 | 1:138290589-138290589 | C | downstream_gene_variant | - | ENSGALG00000055129 | lncRNA | 41856 | - |
| rs314787954 | 1:138290589-138290589 | C | upstream_gene_variant | RASA3 | ENSGALG00000016817 | protein_coding | 57407 | ENSGALP00000027098 |
| rs314787954 | 1:138290589-138290589 | C | downstream_gene_variant | SOWAHC | ENSGALG00000026360 | protein_coding | 90675 | ENSGALP00000042644 |
| rs314787954 | 1:138290589-138290589 | C | upstream_gene_variant | SEPTIN10 | ENSGALG00000016811 | protein_coding | 92397 | ENSGALP00000027091 |
| rs13623466 | 1:129973354-129973354 | T | upstream_gene_variant | ZBED1 | ENSGALG00000016681 | protein_coding | 7631 | ENSGALP00000026866 |
| rs13623466 | 1:129973354-129973354 | T | upstream_gene_variant | ZBED1 | ENSGALG00000016681 | protein_coding | 7631 | ENSGALP00000058272 |
| rs13623466 | 1:129973354-129973354 | T | upstream_gene_variant | ZBED1 | ENSGALG00000016681 | protein_coding | 8229 | ENSGALP00000073586 |
| rs13623466 | 1:129973354-129973354 | T | upstream_gene_variant | ZBED1 | ENSGALG00000016681 | protein_coding | 15250 | ENSGALP00000074009 |
| rs13623466 | 1:129973354-129973354 | T | upstream_gene_variant | ZBED1 | ENSGALG00000016681 | protein_coding | 15250 | ENSGALP00000064909 |
| rs13623466 | 1:129973354-129973354 | T | downstream_gene_variant | - | ENSGALG00000049881 | lncRNA | 36088 | - |
| rs13623466 | 1:129973354-129973354 | T | downstream_gene_variant | - | ENSGALG00000049881 | lncRNA | 36088 | - |
| rs13623466 | 1:129973354-129973354 | T | upstream_gene_variant | CD99 | ENSGALG00000024488 | protein_coding | 86097 | ENSGALP00000066228 |
| rs13623466 | 1:129973354-129973354 | T | upstream_gene_variant | CD99 | ENSGALG00000024488 | protein_coding | 86127 | ENSGALP00000040606 |
| rs13910430 | 1:100656316-100656316 | T | upstream_gene_variant | - | ENSGALG00000015701 | protein_coding | 29169 | ENSGALP00000029745 |
| rs13910430 | 1:100656316-100656316 | T | upstream_gene_variant | - | ENSGALG00000015701 | protein_coding | 36375 | ENSGALP00000065960 |
| rs13910430 | 1:100656316-100656316 | T | downstream_gene_variant | C1H21ORF91 | ENSGALG00000028290 | protein_coding | 93967 | ENSGALP00000041567 |
| rs737507850 | 1:50383576-50383576 | C | downstream_gene_variant | - | ENSGALG00000052047 | lncRNA | 1814 | - |
| rs737507850 | 1:50383576-50383576 | C | upstream_gene_variant | - | ENSGALG00000054068 | lncRNA | 24319 | - |
| rs737507850 | 1:50383576-50383576 | C | upstream_gene_variant | - | ENSGALG00000054068 | lncRNA | 24579 | - |
| rs737507850 | 1:50383576-50383576 | C | downstream_gene_variant | - | ENSGALG00000035051 | pseudogene | 27942 | - |
| rs737507850 | 1:50383576-50383576 | C | downstream_gene_variant | gga-mir-12209 | ENSGALG00000054232 | miRNA | 34824 | - |
| rs737507850 | 1:50383576-50383576 | C | upstream_gene_variant | GRAP2 | ENSGALG00000012056 | protein_coding | 44310 | ENSGALP00000065140 |
| rs737507850 | 1:50383576-50383576 | C | upstream_gene_variant | GRAP2 | ENSGALG00000012056 | protein_coding | 44385 | ENSGALP00000071655 |
| rs737507850 | 1:50383576-50383576 | C | upstream_gene_variant | GRAP2 | ENSGALG00000012056 | protein_coding | 65186 | ENSGALP00000019667 |
| **Suggestive SNPs** |  |  |  |  |  |  |  |  |
| rs736576816 | 2:61140776-61140776 | G | intron_variant | JARID2 | ENSGALG00000012702 | protein_coding | Within | ENSGALP00000020711 |
| rs736576816 | 2:61140776-61140776 | G | downstream_gene_variant | gga-mir-6557 | ENSGALG00000038174 | miRNA | 74245 | - |
| rs736576816 | 2:61140776-61140776 | G | downstream_gene_variant | - | ENSGALG00000051834 | lncRNA | 77120 | - |
| rs736576816 | 2:61140776-61140776 | G | upstream_gene_variant | - | ENSGALG00000052762 | lncRNA | 86132 | - |
| rs736427856 | 2:119443693-119443693 | T | downstream_gene_variant | ZFHX4 | ENSGALG00000037164 | protein_coding | 377 | ENSGALP00000070594 |
| rs736427856 | 2:119443693-119443693 | T | downstream_gene_variant | ZFHX4 | ENSGALG00000037164 | protein_coding | 1377 | ENSGALP00000046296 |
| rs736427856 | 2:119443693-119443693 | T | downstream_gene_variant | - | ENSGALG00000055115 | lncRNA | 18229 | - |
| rs736427856 | 2:119443693-119443693 | T | downstream_gene_variant | PEX2 | ENSGALG00000035752 | protein_coding | 41177 | ENSGALP00000056867 |
| rs1060031521 | 2:93149682-93149682 | T | downstream_gene_variant | - | ENSGALG00000050956 | miRNA | 9248 | - |
| rs1060031521 | 2:93149682-93149682 | T | upstream_gene_variant | - | ENSGALG00000053313 | protein_coding | 89139 | ENSGALP00000068591 |
| rs740392770 | 4:51805377-51805377 | T | upstream_gene_variant | - | ENSGALG00000011812 | protein_coding | 7988 | ENSGALP00000019259 |
| rs740392770 | 4:51805377-51805377 | T | upstream_gene_variant | SULT1B1 | ENSGALG00000023120 | protein_coding | 10251 | ENSGALP00000019258 |
| rs740392770 | 4:51805377-51805377 | T | upstream_gene_variant | SULT1B | ENSGALG00000023122 | protein_coding | 12910 | ENSGALP00000019254 |
| rs740392770 | 4:51805377-51805377 | T | upstream_gene_variant | - | ENSGALG00000011805 | protein_coding | 25690 | ENSGALP00000019241 |
| rs740392770 | 4:51805377-51805377 | T | upstream_gene_variant | - | ENSGALG00000051655 | lncRNA | 28946 | - |
| rs740392770 | 4:51805377-51805377 | T | downstream_gene_variant | YTHDC1 | ENSGALG00000035906 | protein_coding | 36382 | ENSGALP00000055285 |
| rs740392770 | 4:51805377-51805377 | T | downstream_gene_variant | YTHDC1 | ENSGALG00000035906 | protein_coding | 36535 | ENSGALP00000051147 |
| rs740392770 | 4:51805377-51805377 | T | downstream_gene_variant | YTHDC1 | ENSGALG00000035906 | protein_coding | 43196 | ENSGALP00000060750 |
| rs740392770 | 4:51805377-51805377 | T | upstream_gene_variant | - | ENSGALG00000053851 | lncRNA | 46069 | - |
| rs740392770 | 4:51805377-51805377 | T | upstream_gene_variant | - | ENSGALG00000053851 | lncRNA | 46069 | - |
| rs740392770 | 4:51805377-51805377 | T | upstream_gene_variant | - | ENSGALG00000049878 | lncRNA | 73886 | - |
| rs740392770 | 4:51805377-51805377 | T | upstream_gene_variant | - | ENSGALG00000049878 | lncRNA | 73886 | - |
| rs740392770 | 4:51805377-51805377 | T | upstream_gene_variant | - | ENSGALG00000011792 | protein_coding | 87406 | ENSGALP00000019213 |
| rs15900019 | 8:4363454-4363454 | A | intron_variant | TEDC1 | ENSGALG00000027492 | protein_coding | Within | ENSGALP00000042595 |
| rs15900019 | 8:4363454-4363454 | A | downstream_gene_variant | CRIP1 | ENSGALG00000002771 | protein_coding | 8227 | ENSGALP00000004366 |
| rs15900019 | 8:4363454-4363454 | A | downstream_gene_variant | CRIP2 | ENSGALG00000026471 | protein_coding | 17826 | ENSGALP00000004365 |
| rs15900019 | 8:4363454-4363454 | A | downstream_gene_variant | MTA1 | ENSGALG00000002803 | protein_coding | 32635 | ENSGALP00000057894 |
| rs15900019 | 8:4363454-4363454 | A | downstream_gene_variant | MTA1 | ENSGALG00000002803 | protein_coding | 32650 | ENSGALP00000004425 |
| rs15900019 | 8:4363454-4363454 | A | downstream_gene_variant | MTA1 | ENSGALG00000002803 | protein_coding | 34181 | ENSGALP00000055048 |
| rs15900019 | 8:4363454-4363454 | A | upstream_gene_variant | TMEM121 | ENSGALG00000054831 | protein_coding | 94618 | ENSGALP00000070792 |
| rs741342879 | 13:8272081-8272081 | T | upstream_gene_variant | - | ENSGALG00000047226 | lncRNA | 42509 | - |
| rs741342879 | 13:8272081-8272081 | T | downstream_gene_variant | GABRB2 | ENSGALG00000001690 | protein_coding | 67439 | ENSGALP00000054204 |
| rs741342879 | 13:8272081-8272081 | T | downstream_gene_variant | GABRB2 | ENSGALG00000001690 | protein_coding | 69688 | ENSGALP00000002581 |
| rs739117494 | 13:16352161-16352161 | T | 3_prime_UTR_variant | PCBD2 | ENSGALG00000006367 | protein_coding | Within | ENSGALP00000010278 |
| rs739117494 | 13:16352161-16352161 | T | downstream_gene_variant | TXNDC15 | ENSGALG00000039781 | protein_coding | 16468 | ENSGALP00000057379 |
| rs739117494 | 13:16352161-16352161 | T | downstream_gene_variant | C5orf24 | ENSGALG00000006371 | protein_coding | 25530 | ENSGALP00000010282 |
| rs739117494 | 13:16352161-16352161 | T | downstream_gene_variant | PITX1 | ENSGALG00000030237 | protein_coding | 30747 | ENSGALP00000054758 |
| rs739117494 | 13:16352161-16352161 | T | downstream_gene_variant | PITX1 | ENSGALG00000030237 | protein_coding | 31078 | ENSGALP00000047128 |
| rs739117494 | 13:16352161-16352161 | T | downstream_gene_variant | PITX1 | ENSGALG00000030237 | protein_coding | 31128 | ENSGALP00000044159 |
| rs739117494 | 13:16352161-16352161 | T | downstream_gene_variant | DDX46 | ENSGALG00000006404 | protein_coding | 39445 | ENSGALP00000010325 |
| rs739117494 | 13:16352161-16352161 | T | downstream_gene_variant | CAMLG | ENSGALG00000006411 | protein_coding | 60078 | ENSGALP00000010335 |
| rs739117494 | 13:16352161-16352161 | T | downstream_gene_variant | SEC24A | ENSGALG00000006417 | protein_coding | 64601 | ENSGALP00000010342 |
| rs739117494 | 13:16352161-16352161 | T | upstream_gene_variant | SAR1B | ENSGALG00000038753 | protein_coding | 87941 | ENSGALP00000056196 |
| rs739117494 | 13:16352161-16352161 | T | upstream_gene_variant | SAR1B | ENSGALG00000038753 | protein_coding | 88648 | ENSGALP00000055599 |
| rs739117494 | 13:16352161-16352161 | T | downstream_gene_variant | - | ENSGALG00000039105 | lncRNA | 99102 | - |
| rs739117494 | 13:16352161-16352161 | T | downstream_gene_variant | - | ENSGALG00000039105 | lncRNA | 99102 | - |
| rs735333650 | 17:8458273-8458273 | T | intron_variant | UBAC1 | ENSGALG00000042564 | protein_coding | Within | ENSGALP00000054569 |
| rs735333650 | 17:8458273-8458273 | T | intron_variant | UBAC1 | ENSGALG00000042564 | protein_coding | Within | ENSGALP00000045014 |
| rs735333650 | 17:8458273-8458273 | T | intron_variant | UBAC1 | ENSGALG00000042564 | protein_coding | Within | ENSGALP00000072006 |
| rs735333650 | 17:8458273-8458273 | T | upstream_gene_variant | gga-mir-1465 | ENSGALG00000025509 | miRNA | 9756 |  |
| rs735333650 | 17:8458273-8458273 | T | downstream_gene_variant | - | ENSGALG00000054657 | protein_coding | 16643 | ENSGALP00000072709 |
| rs735333650 | 17:8458273-8458273 | T | downstream_gene_variant | NACC2 | ENSGALG00000001728 | protein_coding | 19407 | ENSGALP00000070565 |
| rs735333650 | 17:8458273-8458273 | T | downstream_gene_variant | NACC2 | ENSGALG00000001728 | protein_coding | 19634 | ENSGALP00000065793 |
| rs735333650 | 17:8458273-8458273 | T | downstream_gene_variant | NACC2 | ENSGALG00000001728 | protein_coding | 19743 | ENSGALP00000002649 |
| rs735333650 | 17:8458273-8458273 | T | downstream_gene_variant | NACC2 | ENSGALG00000001728 | protein_coding | 19850 | ENSGALP00000067866 |
| rs735333650 | 17:8458273-8458273 | T | upstream_gene_variant | CAMSAP1 | ENSGALG00000001692 | protein_coding | 20186 | ENSGALP00000002590 |
| rs735333650 | 17:8458273-8458273 | T | upstream_gene_variant | CAMSAP1 | ENSGALG00000001692 | protein_coding | 25335 | ENSGALP00000044107 |
| rs735333650 | 17:8458273-8458273 | T | upstream_gene_variant | CAMSAP1 | ENSGALG00000001692 | protein_coding | 25335 | ENSGALP00000072601 |
| rs735333650 | 17:8458273-8458273 | T | downstream_gene_variant | KCNT1 | ENSGALG00000001645 | protein_coding | 53544 | ENSGALP00000038129 |
| rs735333650 | 17:8458273-8458273 | T | downstream_gene_variant | KCNT1 | ENSGALG00000001645 | protein_coding | 53898 | ENSGALP00000055306 |
| rs735333650 | 17:8458273-8458273 | T | downstream_gene_variant | KCNT1 | ENSGALG00000001645 | protein_coding | 53898 | ENSGALP00000067334 |
| rs735333650 | 17:8458273-8458273 | T | downstream_gene_variant | KCNT1 | ENSGALG00000001645 | protein_coding | 53898 | ENSGALP00000068541 |
| rs735333650 | 17:8458273-8458273 | T | downstream_gene_variant | C9orf69 | ENSGALG00000032627 | protein_coding | 65602 | ENSGALP00000053348 |
| rs735333650 | 17:8458273-8458273 | T | downstream_gene_variant | - | ENSGALG00000049918 | lncRNA | 72304 | - |
| rs735333650 | 17:8458273-8458273 | T | downstream_gene_variant | - | ENSGALG00000053159 | lncRNA | 94450 | - |
| rs14118744 | 19:3534961-3534961 | G | intron_variant | IFT22 | ENSGALG00000021653 | protein_coding | Within | ENSGALP00000034460 |
| rs14118744 | 19:3534961-3534961 | G | intron_variant | CAMKK1 | ENSGALG00000001617 | protein_coding | Within | ENSGALP00000067445 |
| rs14118744 | 19:3534961-3534961 | G | intron_variant | CAMKK1 | ENSGALG00000001617 | protein_coding | Within | ENSGALP00000072527 |
| rs14118744 | 19:3534961-3534961 | G | upstream_gene_variant | DERL2 | ENSGALG00000041844 | protein_coding | 29117 | ENSGALP00000057135 |
| rs14118744 | 19:3534961-3534961 | G | upstream_gene_variant | - | ENSGALG00000052647 | protein_coding | 33889 | ENSGALP00000060273 |
| rs14118744 | 19:3534961-3534961 | G | upstream_gene_variant | C1QBP | ENSGALG00000001654 | protein_coding | 43362 | ENSGALP00000002522 |
| rs14118744 | 19:3534961-3534961 | G | downstream_gene_variant | RPAIN | ENSGALG00000001666 | protein_coding | 46653 | ENSGALP00000002549 |
| rs14118744 | 19:3534961-3534961 | G | upstream_gene_variant | NUP88 | ENSGALG00000032280 | protein_coding | 49225 | ENSGALP00000056538 |
| rs14118744 | 19:3534961-3534961 | G | downstream_gene_variant | RABEP1 | ENSGALG00000001737 | protein_coding | 61411 | ENSGALP00000059063 |
| rs14118744 | 19:3534961-3534961 | G | downstream_gene_variant | P2RX1 | ENSGALG00000001573 | protein_coding | 64667 | ENSGALP00000034476 |
| rs14118744 | 19:3534961-3534961 | G | downstream_gene_variant | ATP2A3 | ENSGALG00000001564 | protein_coding | 79000 | ENSGALP00000002373 |
| rs14118744 | 19:3534961-3534961 | G | downstream_gene_variant | ATP2A3 | ENSGALG00000001564 | protein_coding | 79713 | ENSGALP00000074136 |
| rs14118744 | 19:3534961-3534961 | G | intron_variant | CAMKK1 | ENSGALG00000001617 | protein_coding | Within | ENSGALP00000002465 |
| rs1060144701 | 26:2497950-2497950 | A | intron_variant | RASSF5 | ENSGALG00000038943 | protein_coding | Within | ENSGALP00000055882 |
| rs1060144701 | 26:2497950-2497950 | A | intron_variant | RASSF5 | ENSGALG00000038943 | protein_coding | Within | ENSGALP00000072021 |
| rs1060144701 | 26:2497950-2497950 | A | intron_variant | RASSF5 | ENSGALG00000038943 | protein_coding | Within | ENSGALP00000070224 |
| rs1060144701 | 26:2497950-2497950 | A | downstream_gene_variant | EIF2D | ENSGALG00000000856 | protein_coding | 9166 | ENSGALP00000001253 |
| rs1060144701 | 26:2497950-2497950 | A | downstream_gene_variant | IKBKE | ENSGALG00000013356 | protein_coding | 11143 | ENSGALP00000021757 |
| rs1060144701 | 26:2497950-2497950 | A | upstream_gene_variant | DYRK3 | ENSGALG00000000863 | protein_coding | 19044 | ENSGALP00000001263 |
| rs1060144701 | 26:2497950-2497950 | A | downstream_gene_variant | - | ENSGALG00000000816 | protein_coding | 28248 | ENSGALP00000070104 |
| rs1060144701 | 26:2497950-2497950 | A | downstream_gene_variant | - | ENSGALG00000000816 | protein_coding | 28294 | ENSGALP00000065187 |
| rs1060144701 | 26:2497950-2497950 | A | downstream_gene_variant | - | ENSGALG00000000816 | protein_coding | 28294 | ENSGALP00000067209 |
| rs1060144701 | 26:2497950-2497950 | A | downstream_gene_variant | - | ENSGALG00000000816 | protein_coding | 29294 | ENSGALP00000001187 |
| rs1060144701 | 26:2497950-2497950 | A | upstream_gene_variant | MAPKAPK2 | ENSGALG00000000883 | protein_coding | 37355 | ENSGALP00000001295 |
| rs1060144701 | 26:2497950-2497950 | A | downstream_gene_variant | IL10 | ENSGALG00000000892 | protein_coding | 64751 | ENSGALP00000001310 |
| rs1060144701 | 26:2497950-2497950 | A | upstream_gene_variant | IL19 | ENSGALG00000000911 | protein_coding | 77658 | ENSGALP00000001338 |
| rs1060144701 | 26:2497950-2497950 | A | downstream_gene_variant | PIGR | ENSGALG00000000919 | protein_coding | 82214 | ENSGALP00000001351 |
| rs1060144701 | 26:2497950-2497950 | A | downstream_gene_variant | - | ENSGALG00000049589 | protein_coding | 94484 | ENSGALP00000066483 |
| **Body Weight** |  |  |  |  |  |  |  |  |
| **Significant SNPS** |  |  |  |  |  |  |  |  |
| rs740980181 | 8:5672824-5672824 | A | intron_variant | PBX1 | ENSGALG00000003429 | protein_coding | Within | ENSGALP00000005416 |
| rs740980181 | 8:5672824-5672824 | A | intron_variant | PBX1 | ENSGALG00000003429 | protein_coding | Within | ENSGALP00000021849 |
| rs740980181 | 8:5672824-5672824 | A | downstream_gene_variant | gga-mir-1633 | ENSGALG00000025313 | miRNA | 29432 | - |
| rs740980181 | 8:5672824-5672824 | A | upstream_gene_variant | gga-mir-6665 | ENSGALG00000028814 | miRNA | 75353 | - |
| rs13792572 | 11:10145710-10145710 | G | downstream_gene_variant | GPATCH1 | ENSGALG00000004839 | protein_coding | 7341 | ENSGALP00000007714 |
| rs13792572 | 11:10145710-10145710 | G | upstream_gene_variant | - | ENSGALG00000052771 | lncRNA | 18616 | - |
| rs13792572 | 11:10145710-10145710 | G | upstream_gene_variant | SLC7A10 | ENSGALG00000004729 | protein_coding | 25276 | ENSGALP00000007520 |
| rs13792572 | 11:10145710-10145710 | G | upstream_gene_variant | SLC7A10 | ENSGALG00000004729 | protein_coding | 25276 | ENSGALP00000057646 |
| rs13792572 | 11:10145710-10145710 | G | downstream_gene_variant | LRP3 | ENSGALG00000004853 | protein_coding | 65312 | ENSGALP00000069558 |
| rs13792572 | 11:10145710-10145710 | G | downstream_gene_variant | LRP3 | ENSGALG00000004853 | protein_coding | 65787 | ENSGALP00000007738 |
| rs13792572 | 11:10145710-10145710 | G | downstream_gene_variant | LRP3 | ENSGALG00000004853 | protein_coding | 65787 | ENSGALP00000058513 |
| rs13792573 | 11:10145737-10145737 | A | downstream_gene_variant | GPATCH1 | ENSGALG00000004839 | protein_coding | 7368 | ENSGALP00000007714 |
| rs13792573 | 11:10145737-10145737 | A | upstream_gene_variant | - | ENSGALG00000052771 | lncRNA | 18589 | - |
| rs13792573 | 11:10145737-10145737 | A | upstream_gene_variant | SLC7A10 | ENSGALG00000004729 | protein_coding | 25303 | ENSGALP00000007520 |
| rs13792573 | 11:10145737-10145737 | A | upstream_gene_variant | SLC7A10 | ENSGALG00000004729 | protein_coding | 25303 | ENSGALP00000057646 |
| rs13792573 | 11:10145737-10145737 | A | downstream_gene_variant | LRP3 | ENSGALG00000004853 | protein_coding | 65339 | ENSGALP00000069558 |
| rs13792573 | 11:10145737-10145737 | A | downstream_gene_variant | LRP3 | ENSGALG00000004853 | protein_coding | 65814 | ENSGALP00000007738 |
| rs13792573 | 11:10145737-10145737 | A | downstream_gene_variant | LRP3 | ENSGALG00000004853 | protein_coding | 65814 | ENSGALP00000058513 |
| rs314702374 | 11:15831363-15831363 | G | intron_variant,non_coding_transcript_variant | - | ENSGALG00000053008 | lncRNA | Within | - |
| rs314702374 | 11:15831363-15831363 | G | intron_variant,non_coding_transcript_variant | - | ENSGALG00000053008 | lncRNA | Within | - |
| rs314702374 | 11:15831363-15831363 | G | downstream_gene_variant | - | ENSGALG00000053008 | lncRNA | 18671 | - |
| rs314702374 | 11:15831363-15831363 | G | upstream_gene_variant | MPHOSPH6 | ENSGALG00000031983 | protein_coding | 22019 | ENSGALP00000051969 |
| rs314702374 | 11:15831363-15831363 | G | upstream_gene_variant | MPHOSPH6 | ENSGALG00000031983 | protein_coding | 22038 | ENSGALP00000047225 |
| rs314702374 | 11:15831363-15831363 | G | downstream_gene_variant | PNAT10 | ENSGALG00000005474 | protein_coding | 33472 | ENSGALP00000008780 |
| rs314702374 | 11:15831363-15831363 | G | downstream_gene_variant | NAT | ENSGALG00000005472 | protein_coding | 39253 | ENSGALP00000069401 |
| rs314702374 | 11:15831363-15831363 | G | downstream_gene_variant | NAT | ENSGALG00000005472 | protein_coding | 39359 | ENSGALP00000008778 |
| rs314702374 | 11:15831363-15831363 | G | downstream_gene_variant | PNAT3 | ENSGALG00000005473 | protein_coding | 44681 | ENSGALP00000008779 |
| rs314702374 | 11:15831363-15831363 | G | downstream_gene_variant | PNAT3 | ENSGALG00000005473 | protein_coding | 44683 | ENSGALP00000066462 |
| rs314702374 | 11:15831363-15831363 | G | downstream_gene_variant | HSD17B2 | ENSGALG00000005467 | protein_coding | 74222 | ENSGALP00000008766 |
| rs314702374 | 11:15831363-15831363 | G | upstream_gene_variant | SDR42E1 | ENSGALG00000005456 | protein_coding | 95616 | ENSGALP00000008742 |
| rs14123335 | 19:8356215-8356215 | C | upstream_gene_variant | - | ENSGALG00000041948 | lncRNA | 54 | - |
| rs14123335 | 19:8356215-8356215 | C | downstream_gene_variant | MRM1 | ENSGALG00000039523 | protein_coding | 11278 | ENSGALP00000058906 |
| rs14123335 | 19:8356215-8356215 | C | downstream_gene_variant | DHRS11 | ENSGALG00000005403 | protein_coding | 20687 | ENSGALP00000008664 |
| rs14123335 | 19:8356215-8356215 | C | upstream_gene_variant | - | ENSGALG00000048282 | lncRNA | 21431 | - |
| rs14123335 | 19:8356215-8356215 | C | downstream_gene_variant | - | ENSGALG00000042528 | lncRNA | 24024 | - |
| rs14123335 | 19:8356215-8356215 | C | downstream_gene_variant | GGNBP2 | ENSGALG00000005397 | protein_coding | 43332 | ENSGALP00000066168 |
| rs14123335 | 19:8356215-8356215 | C | downstream_gene_variant | GGNBP2 | ENSGALG00000005397 | protein_coding | 43387 | ENSGALP00000008651 |
| rs14123335 | 19:8356215-8356215 | C | downstream_gene_variant | PIGW | ENSGALG00000023554 | protein_coding | 63556 | ENSGALP00000038373 |
| rs14123335 | 19:8356215-8356215 | C | upstream_gene_variant | MYO19 | ENSGALG00000005374 | protein_coding | 65963 | ENSGALP00000067832 |
| rs14123335 | 19:8356215-8356215 | C | upstream_gene_variant | MYO19 | ENSGALG00000005374 | protein_coding | 65966 | ENSGALP00000038375 |
| rs14123335 | 19:8356215-8356215 | C | downstream_gene_variant | ZNHIT3 | ENSGALG00000026031 | protein_coding | 81595 | ENSGALP00000008613 |
| rs14123335 | 19:8356215-8356215 | C | downstream_gene_variant | ZNHIT3 | ENSGALG00000026031 | protein_coding | 82357 | ENSGALP00000061216 |
| rs14123335 | 19:8356215-8356215 | C | downstream_gene_variant | - | ENSGALG00000005350 | protein_coding | 90049 | ENSGALP00000008566 |
| rs14123335 | 19:8356215-8356215 | C | upstream_gene_variant | CA4 | ENSGALG00000005360 | protein_coding | 93923 | ENSGALP00000008591 |
| **Suggestive SNPs** |  |  |  |  |  |  |  |  |
| rs318161016 | 4:51805422-51805422 | A | upstream_gene_variant | -FGF2 | ENSGALG00000011812 | protein_coding | 8033 | ENSGALP00000019259 |
| rs318161016 | 4:51805422-51805422 | A | upstream_gene_variant | SULT1B1 | ENSGALG00000023120 | protein_coding | 10296 | ENSGALP00000019258 |
| rs318161016 | 4:51805422-51805422 | A | upstream_gene_variant | SULT1B | ENSGALG00000023122 | protein_coding | 12955 | ENSGALP00000019254 |
| rs318161016 | 4:51805422-51805422 | A | upstream_gene_variant | - | ENSGALG00000011805 | protein_coding | 25735 | ENSGALP00000019241 |
| rs318161016 | 4:51805422-51805422 | A | upstream_gene_variant | - | ENSGALG00000051655 | lncRNA | 28901 | - |
| rs318161016 | 4:51805422-51805422 | A | downstream_gene_variant | YTHDC1 | ENSGALG00000035906 | protein_coding | 36427 | ENSGALP00000055285 |
| rs318161016 | 4:51805422-51805422 | A | downstream_gene_variant | YTHDC1 | ENSGALG00000035906 | protein_coding | 36580 | ENSGALP00000051147 |
| rs318161016 | 4:51805422-51805422 | A | downstream_gene_variant | YTHDC1 | ENSGALG00000035906 | protein_coding | 43241 | ENSGALP00000060750 |
| rs318161016 | 4:51805422-51805422 | A | upstream_gene_variant | - | ENSGALG00000053851 | lncRNA | 46024 | - |
| rs318161016 | 4:51805422-51805422 | A | upstream_gene_variant | - | ENSGALG00000053851 | lncRNA | 46024 | - |
| rs318161016 | 4:51805422-51805422 | A | upstream_gene_variant | - | ENSGALG00000049878 | lncRNA | 73841 | - |
| rs318161016 | 4:51805422-51805422 | A | upstream_gene_variant | - | ENSGALG00000049878 | lncRNA | 73841 | - |
| rs318161016 | 4:51805422-51805422 | A | upstream_gene_variant | - | ENSGALG00000011792 | protein_coding | 87451 | ENSGALP00000019213 |
