# Supplementary material for: Genome-Wide Association Study of Growth Performance and Immune Response to Newcastle Disease Virus of Indigenous Chicken in Rwanda
Source: Front Genet. 2021 Aug 16;12:723980. doi: 10.3389/fgene.2021.723980 (PMC8570395; doi:10.3389/fgene.2021.723980)
Supplement: Supplementary file 1 [file Data_Sheet_1.zip › Suppl. Table 2.DOC]

| **Cluster 1** | **Enrichment score: 2.2** | **P-Value** | **Bonferroni** | **FDR** | **Genes** |
| --- | --- | --- | --- | --- | --- |
| **GOTERM_MF_DIRECT** | [Arylamine N-acetyltransferase activity](http://www.ebi.ac.uk/QuickGO/GTerm?id=GO:0004060) | 1.20E-04 | 2.30E-02 | 1.50E-01 | NAT  PNAT10  PNAT3 |
| **KEGG_PATHWAY** | [Caffeine metabolism](https://david.ncifcrf.gov/kegg.jsp?path=gga00232$Caffeine%20metabolism&termId=550025550&source=kegg) | 2.80E-04 | 2.90E-02 | 3.10E-01 |  |
| **UP_SEQ_FEATURE** | active site: Acyl-thioester intermediate | 5.90E-04 | 2.70E-02 | 5.60E-01 |  |
| **UP_KEYWORDS** | [Acyltransferase](http://www.uniprot.org/keywords/?query=Acyltransferase) | 8.90E-04 | 1.10E-01 | 1.00E+00 | NAT  PNAT10  PNAT3  PIGW |
| **KEGG_PATHWAY** | [Drug metabolism - other enzymes](https://david.ncifcrf.gov/kegg.jsp?path=gga00983$Drug%20metabolism%20-%20other%20enzymes&termId=550025615&source=kegg) | 4.50E-03 | 3.50E-01 | 4.80E+00 | NAT  PNAT10  PNAT3 |
| **GOTERM_BP_DIRECT** | [metabolic process](http://www.ebi.ac.uk/QuickGO/GTerm?id=GO:0008152) | 1.70E-02 | 5.50E-01 | 1.50E+01 | NAT  PNAT10  PNAT3 |
| **UP_KEYWORDS** | [Transferase](http://www.uniprot.org/keywords/?query=Transferase) | 6.20E-02 | 1.00E+00 | 5.80E+01 | NAT  PNAT10  PNAT3  PIGW  CAMKK1  SULT1B  SULT1B1 |
| **KEGG_PATHWAY** | [Metabolic pathways](https://david.ncifcrf.gov/kegg.jsp?path=gga01100$Metabolic%20pathways&termId=550025617&source=kegg) | 2.40E-01 | 1.00E+00 | 9.50E+01 | NAT  PNAT10  PNAT3  ACACA  GLUL  HSD17B2  PIGW |

**S2 Table. Functional annotation clustering analysis of candidate genes for body weight and antibody response to Newcastle disease in indigenous chicken in Rwanda**

| **Cluster 2** | **Enrichment score: 1.02** | **P-Value** | **Bonferroni** | **FDR** | **Genes** |
| --- | --- | --- | --- | --- | --- |
| **GOTERM_MF_DIRECT** | [transcription factor activity, sequence-specific DNA binding](http://www.ebi.ac.uk/QuickGO/GTerm?id=GO:0003700) | 1.30E-02 | 7.60E-01 | 1.40E+01 | CEBPA, LHX1, MTA1, PITX1, PBX1, RXRG, ZBED1 |
| **INTERPRO** | [Homeodomain-like](http://www.ebi.ac.uk/interpro/IEntry?ac=IPR009057) | 3.80E-02 | 1.00E+00 | 3.90E+01 | LHX1, MTA1, PITX1, PBX1, ZFHX4 |
| **INTERPRO** | [Homeobox, conserved site](http://www.ebi.ac.uk/interpro/IEntry?ac=IPR017970) | 5.40E-02 | 1.00E+00 | 5.00E+01 | LHX1, PITX1, PBX1, ZFHX4 |
| **GOTERM_CC_DIRECT** | [nucleus](http://www.ebi.ac.uk/QuickGO/GTerm?id=GO:0005634) | 7.50E-02 | 1.00E+00 | 5.80E+01 | CEBPA, LHX1, RASSF5.ALB, CALB1,DYRK3, EIF2D, FGF2, GGNBP2, JARID2, MTA1, MX1, PITX1, PBX1, PCBD2, RXRG, ZFHX4, ZBED1 |
| **SMART** | [HOX](http://smart.embl.de/smart/do_annotation.pl?DOMAIN=SM00389) | 7.50E-02 | 9.80E-01 | 5.20E+01 | LHX1, PITX1, PBX1, ZFHX4 |
| **UP_KEYWORDS** | [Homeobox](http://www.uniprot.org/keywords/?query=Homeobox) | 8.00E-02 | 1.00E+00 | 6.00E+01 | LHX1, PITX1, PBX1, ZFHX4 |
| **INTERPRO** | [Homeodomain](http://www.ebi.ac.uk/interpro/IEntry?ac=IPR001356) | 8.50E-02 | 1.00E+00 | 6.70E+01 | LHX1, PITX1, PBX1, ZFHX4 |
| **GOTERM_MF_DIRECT** | [sequence-specific DNA binding](http://www.ebi.ac.uk/QuickGO/GTerm?id=GO:0043565) | 1.40E-01 | 1.00E+00 | 8.10E+01 | LHX1, MTA1, PITX1, PBX1, ZFHX4 |
| **GOTERM_CC_DIRECT** | [transcription factor complex](http://www.ebi.ac.uk/QuickGO/GTerm?id=GO:0005667) | 1.90E-01 | 1.00E+00 | 9.10E+01 | PITX1, PBX1, RXRG |
| **UP_KEYWORDS** | [DNA-binding](http://www.uniprot.org/keywords/?query=DNA-binding) | 4.50E-01 | 1.00E+00 | 1.00E+02 | LHX1, PITX1, PBX1, RXRG, ZFHX4 |
| **UP_KEYWORDS** | Nucleus | 4.8E-1 | 1.0E0 | 1.0E2 | LHX1, FGF2, JARID2, MTA1, PITX1 PBX1, RXRG, ZFHX4 |
| **Cluster 3** | **Enrichment score: 0.94** | **P-Value** | **Bonferroni** | **FDR** | **Genes** |
| **GOTERM__CC_DIRECT** | Neuron projection | 2.6E-2 | 9.3E-1 | 2.5E1 | ADCYAP1, CDH13, CDH2, CALB1 |
| **UP_KEYWORDS** | Calcium | 7.2E-2 | 1.0E0 | 5.6E1 | ATP2A3, CDH13, CDH2, CALB1, KCNT1 |
| **GOTERM_MF_DIRECT** | Calcium ion binding | 7.8E-1 | 1.0E0 | 1.0E2 | CDH13, CDH2, CLB1 |

| **Cluster 4** | **Enrichment score: 0.82** | **P-Value** | **Bonferroni** | **FDR** | **Genes** |
| --- | --- | --- | --- | --- | --- |
| **UP_KEYWORDS** | Metal-binding | 7.6E-2 | 1.0E0 | 5.8E1 | ATP2A3, LHX1, ACACA, ALB, CDH13, CDH2, CALB1, CRIP1, PEX2, RXRG, ZFHX4 |
| **GOTERM_MF_DIRECT** | Zinc ion binding | 9.5E-2 | 1.0E0 | 6.8E1 | LHX1, SEC24A, CALB1, CA4, CRIP1, MTA1, PEX2, RXRG, ZFHX4 |
| **UP_KEYWORDS** | Zinc | 4.9E-1 | 1.0E0 | 1.0E2 | LHX1, CRIP1, PEX2, RXRG, ZFHX4 |

| **Cluster 5** | **Enrichment score: 0.65** | **P-Value** | **Bonferroni** | **FDR** | **Genes** |
| --- | --- | --- | --- | --- | --- |
| **UP_KEYWORDS** | Ion transport | 1.7E-1 | 1.0E0 | 8.7E1 | ATP2A3, GABRB2, KCNT1, P2RX1 |
| **UP_KEYWORDS** | Ion chanel | 2.4E-1 | 1.0E0 | 9.5E1 | GABRB2, KCNT1, P2RX1 |
| **UP_KEYWORDS** | Transport | 2.8E-1 | 1.0E0 | 9.7E1 | ATP2A3, SAR1B, SEC24A, GABRB2, KCNTI, P2RX1 |

| **Cluster 6** | **Enrichment score: 0.61** | **P-Value** | **Bonferroni** | **FDR** | **Genes** |
| --- | --- | --- | --- | --- | --- |
| **UP_KEYWORDS** | [extracellular space](http://www.ebi.ac.uk/QuickGO/GTerm?id=GO:0005615) | 3.90E-02 | 9.80E-01 | 3.50E+01 | ADCYAP1, ALB, DHRS11, FGF2, IL10, IL8L2, PRL |
| **GOTERM_CC_DIRECT** | signal peptide | 7.30E-02 | 1.00E+00 | 5.70E+01 | ALB, CDH13, FGF2, IL10, IL8L2, LECT2, PIGR, PRL |
| **UP_SEQ_FEATURE** | [Cytokine-cytokine receptor interaction](https://david.ncifcrf.gov/kegg.jsp?path=gga04060$Cytokine-cytokine%20receptor%20interaction&termId=550025646&source=kegg) | 2.40E-01 | 1.00E+00 | 9.60E+01 | ADCYAP1, ALB, CDH13, CDH2, DHRS11, IL10, IL8L2, LECT2 PRL |
| **KEGG_PATHWAY** | [Signal](http://www.uniprot.org/keywords/?query=Signal) | 3.20E-01 | 1.00E+00 | 9.70E+01 | IL10, IL8L2, PRL |
| **UP_KEYWORDS** | [Disulfide bond](http://www.uniprot.org/keywords/?query=Disulfide%20bond) | 4.70E-01 | 1.00E+00 | 1.00E+02 | CD99, ADCYAP1, ALB, CDH13, CDH2, DHRS11, GABRB2, IL10, IL8L2, LECT2, PIGR, PRL, TXNDC15 |
| **UP_KEYWORDS** | disulfide bond | 6.60E-01 | 1.00E+00 | 1.00E+02 | ALB, GABRB2, IL10, IL8L2, PRL, TMPRSS11F |
| **UP_SEQ_FEATURE** | [extracellular space](http://www.ebi.ac.uk/QuickGO/GTerm?id=GO:0005615) | 8.20E-01 | 1.00E+00 | 1.00E+02 | ALB, IL10, IL8L2, PRL |

| **Cluster 7** | **Enrichment score: 0.43** | **P-Value** | **Bonferroni** | **FDR** | **Genes** |
| --- | --- | --- | --- | --- | --- |
| **UP_KEYWORDS** | [Cell membrane](http://www.uniprot.org/keywords/?query=Cell%20membrane) | 7.20E-02 | 1.00E+00 | 5.60E+01 | ATP2A3, CDH13, CDH2, CALB1, KCNT1 |
| **UP_KEYWORDS** | [Glycoprotein](http://www.uniprot.org/keywords/?query=Glycoprotein) | 5.00E-01 | 1.00E+00 | 1.00E+02 | CDH13, CDH2, GABRB2, KCNT1 |
| **UP_KEYWORDS** | glycosylation site:N-linked (GlcNAc...) | 5.80E-01 | 1.00E+00 | 1.00E+02 | ALB, CDH13, CDH2, KCNT1 |
| **UP_SEQ_FEATURE** | [Cell membrane](http://www.uniprot.org/keywords/?query=Cell%20membrane) | 9.20E-01 | 1.00E+00 | 1.00E+02 | ALB, CDH13, CDH2, KCNT1 |

| **Cluster 8** | **Enrichment score: 0.41** | **P-Value** | **Bonferroni** | **FDR** | **Genes** |
| --- | --- | --- | --- | --- | --- |
| **UP_SEQ_FEATURE** | topological domain:Cytoplasmic | 7.20E-02 | 1.00E+00 | 5.60E+01 | ATP2A3, CDH13, CDH2, CALB1, KCNT1 |
| **UP_SEQ_FEATURE** | transmembrane region | 9.00E-01 | 1.00E+00 | 1.00E+02 | ATP2A3, CDH2, KCNT1 |
| **UP_SEQ_FEATURE** | topological domain:Cytoplasmic | 9.10E-01 | 1.00E+00 | 1.00E+02 | ATP2A3, CDH2, KCNT1, TMEM121 |

| **Cluster 9** | **Enrichment score: 0.4** | **P-Value** | **Bonferroni** | **FDR** | **Genes** |
| --- | --- | --- | --- | --- | --- |
| **GOTERM_MF_DIRECT** | [ATP binding](http://www.ebi.ac.uk/QuickGO/GTerm?id=GO:0005524) | 3.20E-01 | 1.00E+00 | 9.90E+01 | ATP2A3, DDX46, ACACA, CAMKK1, DYRK3, GLUL, IKBKE, MYO19, P2RX1 |
| **UP_KEYWORDS** | [Nucleotide-binding](http://www.uniprot.org/keywords/?query=Nucleotide-binding) | 3.70E-01 | 1.00E+00 | 9.90E+01 | ATP2A3, SAR1B, ACACA, CAMKK1, GLUL, MYO19, MX1 |
| **UP_KEYWORDS** | [ATP-binding](http://www.uniprot.org/keywords/?query=ATP-binding) | 5.40E-01 | 1.00E+00 | 1.00E+02 | ATP2A3, ACACA, CAMKK1, GLUL, MYO19 |

| **Cluster 10** | **Enrichment score: 0.32** | **P-Value** | **Bonferroni** | **FDR** | **Genes** |
| --- | --- | --- | --- | --- | --- |
| **GOTERM_BP_DIRECT** | [transcription, DNA-templated](http://www.ebi.ac.uk/QuickGO/GTerm?id=GO:0006351) | 3.20E-01 | 1.00E+00 | 9.90E+01 | CEBPA, JARID2, PITX1, RXRG, ZFHX4 |
| **UP_KEYWORDS** | [DNA-binding](http://www.uniprot.org/keywords/?query=DNA-binding) | 4.50E-01 | 1.00E+00 | 1.00E+02 | LHX1, PITX1, PBX1, RXRG, ZFHX4 |
| **UP_KEYWORDS** | [Transcription regulation](http://www.uniprot.org/keywords/?query=Transcription%20regulation) | 4.80E-01 | 1.00E+00 | 1.00E+02 | JARID2, PITX1, RXRG, ZFHX4 |
| **UP_KEYWORDS** | [Nucleus](http://www.uniprot.org/keywords/?query=Nucleus) | 4.80E-01 | 1.00E+00 | 1.00E+02 | LHX1, FGF2, JARID2, MTA1, PITX1, PBX1, RXRG, ZFHX4 |
| **UP_KEYWORDS** | [Transcription](http://www.uniprot.org/keywords/?query=Transcription) | 5.10E-01 | 1.00E+00 | 1.00E+02 | JARID2, PITX1, RXRG, ZFHX4 |
| **GOTERM_BP_DIRECT** | [Regulation of transcription, DNA-templated](http://www.ebi.ac.uk/QuickGO/GTerm?id=GO:0006355) | 7.50E-01 | 1.00E+00 | 1.00E+02 | PITX1, RXRG, ZFHX4 |

| **Cluster 11** | **Enrichment score: 0.23** | **P-Value** | **Bonferroni** | **FDR** | **Genes** |
| --- | --- | --- | --- | --- | --- |
| **SMART** | [S_TKc](http://smart.embl.de/smart/do_annotation.pl?DOMAIN=SM00220) | 4.60E-01 | 1.00E+00 | 1.00E+02 | CAMKK1, DYRK3, IKBKE |
| **INTERPRO** | [Protein kinase, ATP binding site](http://www.ebi.ac.uk/interpro/IEntry?ac=IPR017441) | 5.30E-01 | 1.00E+00 | 1.00E+02 | CAMKK1, DYRK3, IKBKE |
| **INTERPRO** | [Protein kinase, catalytic domain](http://www.ebi.ac.uk/interpro/IEntry?ac=IPR000719) | 6.60E-01 | 1.00E+00 | 1.00E+02 | CAMKK1, DYRK3, IKBKE |
| **INTERPRO** | [Protein kinase-like domain](http://www.ebi.ac.uk/interpro/IEntry?ac=IPR011009) | 7.20E-01 | 1.00E+00 | 1.00E+02 | CAMKK1, DYRK3, IKBKE |

| **Cluster 11** | **Enrichment score: 0.11** | **P-Value** | **Bonferroni** | **FDR** | **Genes** |
| --- | --- | --- | --- | --- | --- |
| **UP_KEYWORDS** | [Transmembrane helix](http://www.uniprot.org/keywords/?query=Transmembrane%20helix) | 7.40E-01 | 1.00E+00 | 1.00E+02 | ATP2A3, CD99, DERL2, CDH2, FMO3, GABRB2, HSD17B2, PIGW, PIGR, KCNT1, P2RX1, SDR42E1, SLC7A10, SPNS3, TXNDC15, TMPRSS11F, TMEM121 |
| **UP_KEYWORDS** | [Transmembrane](http://www.uniprot.org/keywords/?query=Transmembrane) | 7.40E-01 | 1.00E+00 | 1.00E+02 | ATP2A3, CD99, DERL2, CDH2, FMO3, GABRB2, HSD17B2, PIGW, PIGR, KCNT1, P2RX1, SDR42E1, SLC7A10, SPNS3, TXNDC15, TMPRSS11F, TMEM121 |
| **UP_KEYWORDS** | [Membrane](http://www.uniprot.org/keywords/?query=Membrane) | 8.00E-01 | 1.00E+00 | 1.00E+02 | ATP2A3, CD99, DERL2, CDH13, CDH2, FMO3, GABRB2, HSD17B2, PIGW, PIGR, KCNT1, P2RX1, SDR42E1, SLC7A10, SPNS3, TXNDC15, TMPRSS11F, TMEM121 |
| **GOTERM_CC_DIRECT** | [integral component of membrane](http://www.ebi.ac.uk/QuickGO/GTerm?id=GO:0016021) | 8.30E-01 | 1.00E+00 | 1.00E+02 | ATP2A3, CD99, CDH2, FMO3, GABRB2, HSD17B2, PIGW, PIGR, KCNT1, SDR42E1, SPNS3, TXNDC15, TMEM121 |
