# Supplementary material for: Genome-Wide Association Study of Growth Performance and Immune Response to Newcastle Disease Virus of Indigenous Chicken in Rwanda
Source: Front Genet. 2021 Aug 16;12:723980. doi: 10.3389/fgene.2021.723980 (PMC8570395; doi:10.3389/fgene.2021.723980)
Supplement: Supplementary file 1 [file Data_Sheet_1.zip › Suppl. Table 3.DOC]

| **S3. Table. Biological functions of genes mapped nearby the significant SNPs for body weight and antibody response to Newcastle disease in indigenous chicken in Rwanda** | |
| --- | --- |
| **ATP2A3** | [**ATPase, Ca++ transporting, ubiquitous(ATP2A3)**](https://david.ncifcrf.gov/geneReportFull.jsp?rowids=395707) |
| **GOTERM_BP_DIRECT** | [cellular calcium ion homeostasis](http://www.ebi.ac.uk/QuickGO/GTerm?id=GO:0006874), [calcium ion transmembrane transport](http://www.ebi.ac.uk/QuickGO/GTerm?id=GO:0070588), |
| **GOTERM_CC_DIRECT** | [integral component of plasma membrane](http://www.ebi.ac.uk/QuickGO/GTerm?id=GO:0005887), [integral component of membrane](http://www.ebi.ac.uk/QuickGO/GTerm?id=GO:0016021), [sarcoplasmic reticulum](http://www.ebi.ac.uk/QuickGO/GTerm?id=GO:0016529), [sarcoplasmic reticulum membrane](http://www.ebi.ac.uk/QuickGO/GTerm?id=GO:0033017), |
| **GOTERM_MF_DIRECT** | [calcium-transporting ATPase activity](http://www.ebi.ac.uk/QuickGO/GTerm?id=GO:0005388), [ATP binding](http://www.ebi.ac.uk/QuickGO/GTerm?id=GO:0005524), [metal ion binding](http://www.ebi.ac.uk/QuickGO/GTerm?id=GO:0046872), |
| **INTERPRO** | [Cation-transporting P-type ATPase](http://www.ebi.ac.uk/interpro/IEntry?ac=IPR001757), [Cation-transporting P-type ATPase, N-terminal](http://www.ebi.ac.uk/interpro/IEntry?ac=IPR004014), [Calcium-transporting P-type ATPase, subfamily IIA, SERCA-type](http://www.ebi.ac.uk/interpro/IEntry?ac=IPR005782), [Cation-transporting P-type ATPase, C-terminal](http://www.ebi.ac.uk/interpro/IEntry?ac=IPR006068), [P-type ATPase, A domain](http://www.ebi.ac.uk/interpro/IEntry?ac=IPR008250), [P-type ATPase, phosphorylation site](http://www.ebi.ac.uk/interpro/IEntry?ac=IPR018303), [HAD-like domain](http://www.ebi.ac.uk/interpro/IEntry?ac=IPR023214), [P-type ATPase, transmembrane domain](http://www.ebi.ac.uk/interpro/IEntry?ac=IPR023298), [P-type ATPase, cytoplasmic domain N](http://www.ebi.ac.uk/interpro/IEntry?ac=IPR023299), |
| **KEGG_PATHWAY** | [Calcium signaling pathway](https://david.ncifcrf.gov/kegg.jsp?path=gga04020$Calcium%20signaling%20pathway&termId=550025645&source=kegg), |
| **SMART** | [SM00831](http://smart.embl.de/smart/do_annotation.pl?DOMAIN=SM00831), |
| **UP_KEYWORDS** | [Alternative splicing](http://www.uniprot.org/keywords/?query=Alternative%20splicing), [ATP-binding](http://www.uniprot.org/keywords/?query=ATP-binding), [Calcium](http://www.uniprot.org/keywords/?query=Calcium), [Calcium transport](http://www.uniprot.org/keywords/?query=Calcium%20transport), [Complete proteome](http://www.uniprot.org/keywords/?query=Complete%20proteome), [Endoplasmic reticulum](http://www.uniprot.org/keywords/?query=Endoplasmic%20reticulum), [Hydrolase](http://www.uniprot.org/keywords/?query=Hydrolase), [Ion transport](http://www.uniprot.org/keywords/?query=Ion%20transport), [Magnesium](http://www.uniprot.org/keywords/?query=Magnesium), [Membrane](http://www.uniprot.org/keywords/?query=Membrane), [Metal-binding](http://www.uniprot.org/keywords/?query=Metal-binding), [Nucleotide-binding](http://www.uniprot.org/keywords/?query=Nucleotide-binding), [Phosphoprotein](http://www.uniprot.org/keywords/?query=Phosphoprotein), [Reference proteome](http://www.uniprot.org/keywords/?query=Reference%20proteome), [Sarcoplasmic reticulum](http://www.uniprot.org/keywords/?query=Sarcoplasmic%20reticulum), [Transmembrane](http://www.uniprot.org/keywords/?query=Transmembrane), [Transmembrane helix](http://www.uniprot.org/keywords/?query=Transmembrane%20helix), [Transport](http://www.uniprot.org/keywords/?query=Transport), |
| **UP_SEQ_FEATURE** | active site:4-aspartylphosphate intermediate, binding site:ATP, chain:Sarcoplasmic/endoplasmic reticulum calcium ATPase 3, metal ion-binding site:Calcium 1, metal ion-binding site:Calcium 2, metal ion-binding site:Calcium 2; via carbonyl oxygen, metal ion-binding site:Magnesium, splice variant, topological domain:Cytoplasmic, topological domain:Lumenal, transmembrane region, |
| **CEBPA** | [**CCAAT/enhancer binding protein (C/EBP), alpha(CEBPA)**](https://david.ncifcrf.gov/geneReportFull.jsp?rowids=427549) |
| **GOTERM_BP_DIRECT** | [transcription, DNA-templated](http://www.ebi.ac.uk/QuickGO/GTerm?id=GO:0006351), [positive regulation of gene expression](http://www.ebi.ac.uk/QuickGO/GTerm?id=GO:0010628), |
| **GOTERM_CC_DIRECT** | [nucleus](http://www.ebi.ac.uk/QuickGO/GTerm?id=GO:0005634), |
| **GOTERM_MF_DIRECT** | [core promoter sequence-specific DNA binding](http://www.ebi.ac.uk/QuickGO/GTerm?id=GO:0001046), [transcription factor activity, sequence-specific DNA binding](http://www.ebi.ac.uk/QuickGO/GTerm?id=GO:0003700), [promoter-specific chromatin binding](http://www.ebi.ac.uk/QuickGO/GTerm?id=GO:1990841), |
| **INTERPRO** | [Basic-leucine zipper domain](http://www.ebi.ac.uk/interpro/IEntry?ac=IPR004827), [CCAAT/enhancer-binding](http://www.ebi.ac.uk/interpro/IEntry?ac=IPR016468), |
| **PIR_SUPERFAMILY** | [CCAAT/enhancer-binding protein](http://pir.georgetown.edu/cgi-bin/ipcSF?id=PIRSF005879), |
| **SMART** | [BRLZ](http://smart.embl.de/smart/do_annotation.pl?DOMAIN=SM00338), |
| **UP_KEYWORDS** | [Coiled coil](http://www.uniprot.org/keywords/?query=Coiled%20coil), |
| **CD99** | [**CD99 molecule(CD99)**](https://david.ncifcrf.gov/geneReportFull.jsp?rowids=418662) |
| **GOTERM_CC_DIRECT** | [focal adhesion](http://www.ebi.ac.uk/QuickGO/GTerm?id=GO:0005925), [integral component of membrane](http://www.ebi.ac.uk/QuickGO/GTerm?id=GO:0016021), |
| **INTERPRO** | [CD99 antigen-like protein 2](http://www.ebi.ac.uk/interpro/IEntry?ac=IPR022078), |
| **KEGG_PATHWAY** | [Cell adhesion molecules (CAMs)](https://david.ncifcrf.gov/kegg.jsp?path=gga04514$Cell%20adhesion%20molecules%20(CAMs)&termId=550025675&source=kegg), |
| **UP_KEYWORDS** | [Complete proteome](http://www.uniprot.org/keywords/?query=Complete%20proteome), [Membrane](http://www.uniprot.org/keywords/?query=Membrane), [Reference proteome](http://www.uniprot.org/keywords/?query=Reference%20proteome), [Signal](http://www.uniprot.org/keywords/?query=Signal), [Transmembrane](http://www.uniprot.org/keywords/?query=Transmembrane), [Transmembrane helix](http://www.uniprot.org/keywords/?query=Transmembrane%20helix), |
| **DDX46** | [**DEAD (Asp-Glu-Ala-Asp) box polypeptide 46(DDX46)**](https://david.ncifcrf.gov/geneReportFull.jsp?rowids=416312) |
| **COG_ONTOLOGY** | [DNA replication, recombination, and repair / Transcription / Translation, ribosomal structure and biogenesis](http://www.ncbi.nlm.nih.gov/COG/new/), |
| **GOTERM_BP_DIRECT** | [mRNA splicing, via spliceosome](http://www.ebi.ac.uk/QuickGO/GTerm?id=GO:0000398), [regulation of gene expression](http://www.ebi.ac.uk/QuickGO/GTerm?id=GO:0010468), [RNA secondary structure unwinding](http://www.ebi.ac.uk/QuickGO/GTerm?id=GO:0010501), |
| **GOTERM_MF_DIRECT** | [nucleic acid binding](http://www.ebi.ac.uk/QuickGO/GTerm?id=GO:0003676), [ATP-dependent RNA helicase activity](http://www.ebi.ac.uk/QuickGO/GTerm?id=GO:0004004), [ATP binding](http://www.ebi.ac.uk/QuickGO/GTerm?id=GO:0005524), |
| **INTERPRO** | [RNA helicase, ATP-dependent, DEAD-box, conserved site](http://www.ebi.ac.uk/interpro/IEntry?ac=IPR000629), [Helicase, C-terminal](http://www.ebi.ac.uk/interpro/IEntry?ac=IPR001650), [DNA/RNA helicase, DEAD/DEAH box type, N-terminal](http://www.ebi.ac.uk/interpro/IEntry?ac=IPR011545), [Helicase, superfamily 1/2, ATP-binding domain](http://www.ebi.ac.uk/interpro/IEntry?ac=IPR014001), [RNA helicase, DEAD-box type, Q motif](http://www.ebi.ac.uk/interpro/IEntry?ac=IPR014014), [P-loop containing nucleoside triphosphate hydrolase](http://www.ebi.ac.uk/interpro/IEntry?ac=IPR027417), |
| **KEGG_PATHWAY** | [Spliceosome](https://david.ncifcrf.gov/kegg.jsp?path=gga03040$Spliceosome&termId=550025633&source=kegg), |
| **SMART** | [DEXDc](http://smart.embl.de/smart/do_annotation.pl?DOMAIN=SM00487), [HELICc](http://smart.embl.de/smart/do_annotation.pl?DOMAIN=SM00490), |
| **UP_KEYWORDS** | [Complete proteome](http://www.uniprot.org/keywords/?query=Complete%20proteome), [Reference proteome](http://www.uniprot.org/keywords/?query=Reference%20proteome), |
| **DERL2** | [**Der1-like domain family, member 2(DERL2)**](https://david.ncifcrf.gov/geneReportFull.jsp?rowids=417498) |
| **GOTERM_BP_DIRECT** | [positive regulation of cell proliferation](http://www.ebi.ac.uk/QuickGO/GTerm?id=GO:0008284), [positive regulation of cell growth](http://www.ebi.ac.uk/QuickGO/GTerm?id=GO:0030307), [ER-associated ubiquitin-dependent protein catabolic process](http://www.ebi.ac.uk/QuickGO/GTerm?id=GO:0030433), [endoplasmic reticulum unfolded protein response](http://www.ebi.ac.uk/QuickGO/GTerm?id=GO:0030968), [retrograde protein transport, ER to cytosol](http://www.ebi.ac.uk/QuickGO/GTerm?id=GO:0030970), [negative regulation of retrograde protein transport, ER to cytosol](http://www.ebi.ac.uk/QuickGO/GTerm?id=GO:1904153), |
| **GOTERM_CC_DIRECT** | [early endosome](http://www.ebi.ac.uk/QuickGO/GTerm?id=GO:0005769), [late endosome](http://www.ebi.ac.uk/QuickGO/GTerm?id=GO:0005770), [integral component of endoplasmic reticulum membrane](http://www.ebi.ac.uk/QuickGO/GTerm?id=GO:0030176), |
| **INTERPRO** | [Derlin](http://www.ebi.ac.uk/interpro/IEntry?ac=IPR007599), |
| **UP_KEYWORDS** | [Complete proteome](http://www.uniprot.org/keywords/?query=Complete%20proteome), [Endoplasmic reticulum](http://www.uniprot.org/keywords/?query=Endoplasmic%20reticulum), [Membrane](http://www.uniprot.org/keywords/?query=Membrane), [Reference proteome](http://www.uniprot.org/keywords/?query=Reference%20proteome), [Transmembrane](http://www.uniprot.org/keywords/?query=Transmembrane), [Transmembrane helix](http://www.uniprot.org/keywords/?query=Transmembrane%20helix), |
| **GPATCH1** | [**G-patch domain containing 1(GPATCH1)**](https://david.ncifcrf.gov/geneReportFull.jsp?rowids=415772) |
| **GOTERM_BP_DIRECT** | [mRNA processing](http://www.ebi.ac.uk/QuickGO/GTerm?id=GO:0006397), |
| **GOTERM_CC_DIRECT** | [catalytic step 2 spliceosome](http://www.ebi.ac.uk/QuickGO/GTerm?id=GO:0071013), |
| **GOTERM_MF_DIRECT** | [nucleic acid binding](http://www.ebi.ac.uk/QuickGO/GTerm?id=GO:0003676), |
| **INTERPRO** | [G-patch domain](http://www.ebi.ac.uk/interpro/IEntry?ac=IPR000467), [Domain of unknown function DUF1604](http://www.ebi.ac.uk/interpro/IEntry?ac=IPR011666), |
| **UP_KEYWORDS** | [Complete proteome](http://www.uniprot.org/keywords/?query=Complete%20proteome), [Reference proteome](http://www.uniprot.org/keywords/?query=Reference%20proteome), |
| **LHX1** | [**LIM homeobox 1(LHX1)**](https://david.ncifcrf.gov/geneReportFull.jsp?rowids=396381) |
| **GOTERM_BP_DIRECT** | [urogenital system development](http://www.ebi.ac.uk/QuickGO/GTerm?id=GO:0001655), [ureteric bud development](http://www.ebi.ac.uk/QuickGO/GTerm?id=GO:0001657), [gastrulation with mouth forming second](http://www.ebi.ac.uk/QuickGO/GTerm?id=GO:0001702), [ectoderm formation](http://www.ebi.ac.uk/QuickGO/GTerm?id=GO:0001705), [endoderm formation](http://www.ebi.ac.uk/QuickGO/GTerm?id=GO:0001706), [kidney development](http://www.ebi.ac.uk/QuickGO/GTerm?id=GO:0001822), [transcription from RNA polymerase II promoter](http://www.ebi.ac.uk/QuickGO/GTerm?id=GO:0006366), [cell-cell signaling](http://www.ebi.ac.uk/QuickGO/GTerm?id=GO:0007267), [pattern specification process](http://www.ebi.ac.uk/QuickGO/GTerm?id=GO:0007389), [motor neuron axon guidance](http://www.ebi.ac.uk/QuickGO/GTerm?id=GO:0008045), [anatomical structure morphogenesis](http://www.ebi.ac.uk/QuickGO/GTerm?id=GO:0009653), [post-embryonic development](http://www.ebi.ac.uk/QuickGO/GTerm?id=GO:0009791), [embryonic pattern specification](http://www.ebi.ac.uk/QuickGO/GTerm?id=GO:0009880), [anterior/posterior axis specification](http://www.ebi.ac.uk/QuickGO/GTerm?id=GO:0009948), [anterior/posterior pattern specification](http://www.ebi.ac.uk/QuickGO/GTerm?id=GO:0009952), [dorsal/ventral pattern formation](http://www.ebi.ac.uk/QuickGO/GTerm?id=GO:0009953), [regulation of gene expression](http://www.ebi.ac.uk/QuickGO/GTerm?id=GO:0010468), [retina layer formation](http://www.ebi.ac.uk/QuickGO/GTerm?id=GO:0010842), [ventral spinal cord development](http://www.ebi.ac.uk/QuickGO/GTerm?id=GO:0021517), [spinal cord motor neuron differentiation](http://www.ebi.ac.uk/QuickGO/GTerm?id=GO:0021522), [spinal cord association neuron differentiation](http://www.ebi.ac.uk/QuickGO/GTerm?id=GO:0021527), [cerebellum development](http://www.ebi.ac.uk/QuickGO/GTerm?id=GO:0021549), [cerebellar Purkinje cell differentiation](http://www.ebi.ac.uk/QuickGO/GTerm?id=GO:0021702), [forebrain regionalization](http://www.ebi.ac.uk/QuickGO/GTerm?id=GO:0021871), [cerebellar Purkinje cell-granule cell precursor cell signaling involved in regulation of granule cell precursor cell proliferation](http://www.ebi.ac.uk/QuickGO/GTerm?id=GO:0021937), [response to retinoic acid](http://www.ebi.ac.uk/QuickGO/GTerm?id=GO:0032526), [positive regulation of embryonic development](http://www.ebi.ac.uk/QuickGO/GTerm?id=GO:0040019), [negative regulation of transcription, DNA-templated](http://www.ebi.ac.uk/QuickGO/GTerm?id=GO:0045892), [positive regulation of transcription, DNA-templated](http://www.ebi.ac.uk/QuickGO/GTerm?id=GO:0045893), [anatomical structure formation involved in morphogenesis](http://www.ebi.ac.uk/QuickGO/GTerm?id=GO:0048646), [embryonic viscerocranium morphogenesis](http://www.ebi.ac.uk/QuickGO/GTerm?id=GO:0048703), [embryonic retina morphogenesis in camera-type eye](http://www.ebi.ac.uk/QuickGO/GTerm?id=GO:0060059), [head development](http://www.ebi.ac.uk/QuickGO/GTerm?id=GO:0060322), [epithelium development](http://www.ebi.ac.uk/QuickGO/GTerm?id=GO:0060429), [kidney morphogenesis](http://www.ebi.ac.uk/QuickGO/GTerm?id=GO:0060993), [paramesonephric duct development](http://www.ebi.ac.uk/QuickGO/GTerm?id=GO:0061205), [comma-shaped body morphogenesis](http://www.ebi.ac.uk/QuickGO/GTerm?id=GO:0072049), [S-shaped body morphogenesis](http://www.ebi.ac.uk/QuickGO/GTerm?id=GO:0072050), [renal vesicle morphogenesis](http://www.ebi.ac.uk/QuickGO/GTerm?id=GO:0072077), [nephric duct morphogenesis](http://www.ebi.ac.uk/QuickGO/GTerm?id=GO:0072178), [nephric duct formation](http://www.ebi.ac.uk/QuickGO/GTerm?id=GO:0072179), [primitive streak formation](http://www.ebi.ac.uk/QuickGO/GTerm?id=GO:0090009), [positive regulation of branching involved in ureteric bud morphogenesis](http://www.ebi.ac.uk/QuickGO/GTerm?id=GO:0090190), [lateral motor column neuron migration](http://www.ebi.ac.uk/QuickGO/GTerm?id=GO:0097477), [positive regulation of gastrulation](http://www.ebi.ac.uk/QuickGO/GTerm?id=GO:2000543), [positive regulation of anterior head development](http://www.ebi.ac.uk/QuickGO/GTerm?id=GO:2000744), [positive regulation of nephron tubule epithelial cell differentiation](http://www.ebi.ac.uk/QuickGO/GTerm?id=GO:2000768), [regulation of RNA biosynthetic process](http://www.ebi.ac.uk/QuickGO/GTerm?id=GO:2001141), |
| **GOTERM_CC_DIRECT** | [nucleus](http://www.ebi.ac.uk/QuickGO/GTerm?id=GO:0005634), [protein complex](http://www.ebi.ac.uk/QuickGO/GTerm?id=GO:0043234), |
| **GOTERM_MF_DIRECT** | [transcription factor activity, sequence-specific DNA binding](http://www.ebi.ac.uk/QuickGO/GTerm?id=GO:0003700), [transcription corepressor activity](http://www.ebi.ac.uk/QuickGO/GTerm?id=GO:0003714), [zinc ion binding](http://www.ebi.ac.uk/QuickGO/GTerm?id=GO:0008270), [sequence-specific DNA binding](http://www.ebi.ac.uk/QuickGO/GTerm?id=GO:0043565), |
| **INTERPRO** | [Homeodomain](http://www.ebi.ac.uk/interpro/IEntry?ac=IPR001356), [Zinc finger, LIM-type](http://www.ebi.ac.uk/interpro/IEntry?ac=IPR001781), [Homeodomain-like](http://www.ebi.ac.uk/interpro/IEntry?ac=IPR009057), [Homeobox, conserved site](http://www.ebi.ac.uk/interpro/IEntry?ac=IPR017970), |
| **SMART** | [LIM](http://smart.embl.de/smart/do_annotation.pl?DOMAIN=SM00132), [HOX](http://smart.embl.de/smart/do_annotation.pl?DOMAIN=SM00389), |
| **UP_KEYWORDS** | [Complete proteome](http://www.uniprot.org/keywords/?query=Complete%20proteome), [DNA-binding](http://www.uniprot.org/keywords/?query=DNA-binding), [Homeobox](http://www.uniprot.org/keywords/?query=Homeobox), [LIM domain](http://www.uniprot.org/keywords/?query=LIM%20domain), [Metal-binding](http://www.uniprot.org/keywords/?query=Metal-binding), [Nucleus](http://www.uniprot.org/keywords/?query=Nucleus), [Reference proteome](http://www.uniprot.org/keywords/?query=Reference%20proteome), [Repeat](http://www.uniprot.org/keywords/?query=Repeat), [Zinc](http://www.uniprot.org/keywords/?query=Zinc), |
| **UP_SEQ_FEATURE** | chain:LIM/homeobox protein Lhx1, DNA-binding region:Homeobox, domain:LIM zinc-binding 1, domain:LIM zinc-binding 2, |
| **MPHOSPH6** | [**M-phase phosphoprotein 6(MPHOSPH6)**](https://david.ncifcrf.gov/geneReportFull.jsp?rowids=415811) |
| **GOTERM_BP_DIRECT** | [maturation of 5.8S rRNA](http://www.ebi.ac.uk/QuickGO/GTerm?id=GO:0000460), |
| **GOTERM_CC_DIRECT** | [nuclear exosome (RNase complex)](http://www.ebi.ac.uk/QuickGO/GTerm?id=GO:0000176), [nucleolus](http://www.ebi.ac.uk/QuickGO/GTerm?id=GO:0005730), [cytoplasm](http://www.ebi.ac.uk/QuickGO/GTerm?id=GO:0005737), |
| **INTERPRO** | [M-phase phosphoprotein 6](http://www.ebi.ac.uk/interpro/IEntry?ac=IPR019324), |
| **KEGG_PATHWAY** | [RNA degradation](https://david.ncifcrf.gov/kegg.jsp?path=gga03018$RNA%20degradation&termId=550025629&source=kegg), |
| **UP_KEYWORDS** | [Complete proteome](http://www.uniprot.org/keywords/?query=Complete%20proteome), [Reference proteome](http://www.uniprot.org/keywords/?query=Reference%20proteome), |
| **NAT** | [**N-acetyltransferase, liver isozyme(NAT)**](https://david.ncifcrf.gov/geneReportFull.jsp?rowids=396537) |
| **GOTERM_BP_DIRECT** | [metabolic process](http://www.ebi.ac.uk/QuickGO/GTerm?id=GO:0008152), |
| **GOTERM_MF_DIRECT** | [arylamine N-acetyltransferase activity](http://www.ebi.ac.uk/QuickGO/GTerm?id=GO:0004060), [acetyltransferase activity](http://www.ebi.ac.uk/QuickGO/GTerm?id=GO:0016407), |
| **INTERPRO** | [Arylamine N-acetyltransferase](http://www.ebi.ac.uk/interpro/IEntry?ac=IPR001447), |
| **KEGG_PATHWAY** | [Caffeine metabolism](https://david.ncifcrf.gov/kegg.jsp?path=gga00232$Caffeine%20metabolism&termId=550025550&source=kegg), [Drug metabolism - other enzymes](https://david.ncifcrf.gov/kegg.jsp?path=gga00983$Drug%20metabolism%20-%20other%20enzymes&termId=550025615&source=kegg), [Metabolic pathways](https://david.ncifcrf.gov/kegg.jsp?path=gga01100$Metabolic%20pathways&termId=550025617&source=kegg), |
| **UP_KEYWORDS** | [Acyltransferase](http://www.uniprot.org/keywords/?query=Acyltransferase), [Complete proteome](http://www.uniprot.org/keywords/?query=Complete%20proteome), [Direct protein sequencing](http://www.uniprot.org/keywords/?query=Direct%20protein%20sequencing), [Reference proteome](http://www.uniprot.org/keywords/?query=Reference%20proteome), [Transferase](http://www.uniprot.org/keywords/?query=Transferase), |
| **UP_SEQ_FEATURE** | active site:Acyl-thioester intermediate, chain:Arylamine N-acetyltransferase, liver isozyme, |
| **PNAT10** | [**N-acetyltransferase, pineal gland isozyme NAT-10(PNAT10)**](https://david.ncifcrf.gov/geneReportFull.jsp?rowids=396283) |
| **GOTERM_BP_DIRECT** | [metabolic process](http://www.ebi.ac.uk/QuickGO/GTerm?id=GO:0008152), |
| **GOTERM_MF_DIRECT** | [arylamine N-acetyltransferase activity](http://www.ebi.ac.uk/QuickGO/GTerm?id=GO:0004060), [acetyltransferase activity](http://www.ebi.ac.uk/QuickGO/GTerm?id=GO:0016407), |
| **INTERPRO** | [Arylamine N-acetyltransferase](http://www.ebi.ac.uk/interpro/IEntry?ac=IPR001447), |
| **KEGG_PATHWAY** | [Caffeine metabolism](https://david.ncifcrf.gov/kegg.jsp?path=gga00232$Caffeine%20metabolism&termId=550025550&source=kegg), [Drug metabolism - other enzymes](https://david.ncifcrf.gov/kegg.jsp?path=gga00983$Drug%20metabolism%20-%20other%20enzymes&termId=550025615&source=kegg), [Metabolic pathways](https://david.ncifcrf.gov/kegg.jsp?path=gga01100$Metabolic%20pathways&termId=550025617&source=kegg), |
| **UP_KEYWORDS** | [Acyltransferase](http://www.uniprot.org/keywords/?query=Acyltransferase), [Complete proteome](http://www.uniprot.org/keywords/?query=Complete%20proteome), [Reference proteome](http://www.uniprot.org/keywords/?query=Reference%20proteome), [Transferase](http://www.uniprot.org/keywords/?query=Transferase), |
| **UP_SEQ_FEATURE** | active site:Acyl-thioester intermediate, chain:Arylamine N-acetyltransferase, pineal gland isozyme NAT-10, |
| **PNAT3** | [**N-acetyltransferase, pineal gland isozyme NAT-3(PNAT3)**](https://david.ncifcrf.gov/geneReportFull.jsp?rowids=415809) |
| **GOTERM_BP_DIRECT** | [metabolic process](http://www.ebi.ac.uk/QuickGO/GTerm?id=GO:0008152), |
| **GOTERM_MF_DIRECT** | [arylamine N-acetyltransferase activity](http://www.ebi.ac.uk/QuickGO/GTerm?id=GO:0004060), |
| **INTERPRO** | [Arylamine N-acetyltransferase](http://www.ebi.ac.uk/interpro/IEntry?ac=IPR001447), |
| **KEGG_PATHWAY** | [Caffeine metabolism](https://david.ncifcrf.gov/kegg.jsp?path=gga00232$Caffeine%20metabolism&termId=550025550&source=kegg), [Drug metabolism - other enzymes](https://david.ncifcrf.gov/kegg.jsp?path=gga00983$Drug%20metabolism%20-%20other%20enzymes&termId=550025615&source=kegg), [Metabolic pathways](https://david.ncifcrf.gov/kegg.jsp?path=gga01100$Metabolic%20pathways&termId=550025617&source=kegg), |
| **UP_KEYWORDS** | [Acyltransferase](http://www.uniprot.org/keywords/?query=Acyltransferase), [Complete proteome](http://www.uniprot.org/keywords/?query=Complete%20proteome), [Reference proteome](http://www.uniprot.org/keywords/?query=Reference%20proteome), [Transferase](http://www.uniprot.org/keywords/?query=Transferase), |
| **UP_SEQ_FEATURE** | active site:Acyl-thioester intermediate, chain:Arylamine N-acetyltransferase, pineal gland isozyme NAT-3, |
| **NACC2** | [**NACC family member 2, BEN and BTB (POZ) domain containing(NACC2)**](https://david.ncifcrf.gov/geneReportFull.jsp?rowids=417128) |
| **GOTERM_BP_DIRECT** | [negative regulation of cell proliferation](http://www.ebi.ac.uk/QuickGO/GTerm?id=GO:0008285), [posttranscriptional regulation of gene expression](http://www.ebi.ac.uk/QuickGO/GTerm?id=GO:0010608), [cellular protein complex localization](http://www.ebi.ac.uk/QuickGO/GTerm?id=GO:0034629), [protein homooligomerization](http://www.ebi.ac.uk/QuickGO/GTerm?id=GO:0051260), [negative regulation of G1/S transition of mitotic cell cycle by negative regulation of transcription from RNA polymerase II promoter](http://www.ebi.ac.uk/QuickGO/GTerm?id=GO:1900477), [positive regulation of intrinsic apoptotic signaling pathway in response to DNA damage](http://www.ebi.ac.uk/QuickGO/GTerm?id=GO:1902231), |
| **GOTERM_CC_DIRECT** | [nuclear chromatin](http://www.ebi.ac.uk/QuickGO/GTerm?id=GO:0000790), [NuRD complex](http://www.ebi.ac.uk/QuickGO/GTerm?id=GO:0016581), |
| **GOTERM_MF_DIRECT** | [RNA polymerase II core promoter proximal region sequence-specific DNA binding](http://www.ebi.ac.uk/QuickGO/GTerm?id=GO:0000978), [transcription factor activity, RNA polymerase II transcription factor binding](http://www.ebi.ac.uk/QuickGO/GTerm?id=GO:0001076), [transcriptional repressor activity, RNA polymerase II core promoter proximal region sequence-specific binding](http://www.ebi.ac.uk/QuickGO/GTerm?id=GO:0001078), [histone deacetylase activity](http://www.ebi.ac.uk/QuickGO/GTerm?id=GO:0004407), |
| **INTERPRO** | [BTB/POZ-like](http://www.ebi.ac.uk/interpro/IEntry?ac=IPR000210), [BTB/POZ fold](http://www.ebi.ac.uk/interpro/IEntry?ac=IPR011333), [BEN domain](http://www.ebi.ac.uk/interpro/IEntry?ac=IPR018379), |
| **SMART** | [BTB](http://smart.embl.de/smart/do_annotation.pl?DOMAIN=SM00225), [SM01025](http://smart.embl.de/smart/do_annotation.pl?DOMAIN=SM01025), |
| **UP_KEYWORDS** | [Complete proteome](http://www.uniprot.org/keywords/?query=Complete%20proteome), [Reference proteome](http://www.uniprot.org/keywords/?query=Reference%20proteome), |
| **RASA3** | [**RAS p21 protein activator 3(RASA3)**](https://david.ncifcrf.gov/geneReportFull.jsp?rowids=418736) |
| **GOTERM_BP_DIRECT** | [intracellular signal transduction](http://www.ebi.ac.uk/QuickGO/GTerm?id=GO:0035556), [negative regulation of Ras protein signal transduction](http://www.ebi.ac.uk/QuickGO/GTerm?id=GO:0046580), |
| **GOTERM_CC_DIRECT** | [intracellular](http://www.ebi.ac.uk/QuickGO/GTerm?id=GO:0005622), [cytoplasm](http://www.ebi.ac.uk/QuickGO/GTerm?id=GO:0005737), [intrinsic component of the cytoplasmic side of the plasma membrane](http://www.ebi.ac.uk/QuickGO/GTerm?id=GO:0031235), |
| **GOTERM_MF_DIRECT** | [GTPase activator activity](http://www.ebi.ac.uk/QuickGO/GTerm?id=GO:0005096), [calcium-release channel activity](http://www.ebi.ac.uk/QuickGO/GTerm?id=GO:0015278), |
| **INTERPRO** | [C2 calcium-dependent membrane targeting](http://www.ebi.ac.uk/interpro/IEntry?ac=IPR000008), [Zinc finger, Btk motif](http://www.ebi.ac.uk/interpro/IEntry?ac=IPR001562), [Pleckstrin homology domain](http://www.ebi.ac.uk/interpro/IEntry?ac=IPR001849), [Ras GTPase-activating protein](http://www.ebi.ac.uk/interpro/IEntry?ac=IPR001936), [Rho GTPase activation protein](http://www.ebi.ac.uk/interpro/IEntry?ac=IPR008936), [Pleckstrin homology-like domain](http://www.ebi.ac.uk/interpro/IEntry?ac=IPR011993), [Ras GTPase-activating protein, conserved site](http://www.ebi.ac.uk/interpro/IEntry?ac=IPR023152), |
| **SMART** | [BTK](http://smart.embl.de/smart/do_annotation.pl?DOMAIN=SM00107), [PH](http://smart.embl.de/smart/do_annotation.pl?DOMAIN=SM00233), [C2](http://smart.embl.de/smart/do_annotation.pl?DOMAIN=SM00239), [RasGAP](http://smart.embl.de/smart/do_annotation.pl?DOMAIN=SM00323), |
| **UP_KEYWORDS** | [Complete proteome](http://www.uniprot.org/keywords/?query=Complete%20proteome), [GTPase activation](http://www.uniprot.org/keywords/?query=GTPase%20activation), [Reference proteome](http://www.uniprot.org/keywords/?query=Reference%20proteome), |
| **RPAIN** | [**RPA interacting protein(RPAIN)**](https://david.ncifcrf.gov/geneReportFull.jsp?rowids=417500) |
| **UP_KEYWORDS** | [Complete proteome](http://www.uniprot.org/keywords/?query=Complete%20proteome), [Reference proteome](http://www.uniprot.org/keywords/?query=Reference%20proteome), |
| **RASSF5** | [**Ras association domain family member 5(RASSF5)**](https://david.ncifcrf.gov/geneReportFull.jsp?rowids=419844) |
| **GOTERM_BP_DIRECT** | [signal transduction](http://www.ebi.ac.uk/QuickGO/GTerm?id=GO:0007165), [negative regulation of cell proliferation](http://www.ebi.ac.uk/QuickGO/GTerm?id=GO:0008285), [positive regulation of protein ubiquitination](http://www.ebi.ac.uk/QuickGO/GTerm?id=GO:0031398), [regulation of apoptotic process](http://www.ebi.ac.uk/QuickGO/GTerm?id=GO:0042981), [regulation of protein localization to nucleus](http://www.ebi.ac.uk/QuickGO/GTerm?id=GO:1900180), |
| **GOTERM_CC_DIRECT** | [nucleus](http://www.ebi.ac.uk/QuickGO/GTerm?id=GO:0005634), |
| **INTERPRO** | [Ras-association](http://www.ebi.ac.uk/interpro/IEntry?ac=IPR000159), [SARAH domain](http://www.ebi.ac.uk/interpro/IEntry?ac=IPR011524), |
| **SMART** | [RA](http://smart.embl.de/smart/do_annotation.pl?DOMAIN=SM00314), |
| **UP_KEYWORDS** | [Coiled coil](http://www.uniprot.org/keywords/?query=Coiled%20coil), [Complete proteome](http://www.uniprot.org/keywords/?query=Complete%20proteome), [Reference proteome](http://www.uniprot.org/keywords/?query=Reference%20proteome), |
| **SAR1B** | [**SAR1 homolog B (S. cerevisiae)(SAR1B)**](https://david.ncifcrf.gov/geneReportFull.jsp?rowids=416314) |
| **GOTERM_BP_DIRECT** | [intracellular protein transport](http://www.ebi.ac.uk/QuickGO/GTerm?id=GO:0006886), [vesicle-mediated transport](http://www.ebi.ac.uk/QuickGO/GTerm?id=GO:0016192), |
| **GOTERM_CC_DIRECT** | [endoplasmic reticulum](http://www.ebi.ac.uk/QuickGO/GTerm?id=GO:0005783), [Golgi apparatus](http://www.ebi.ac.uk/QuickGO/GTerm?id=GO:0005794), |
| **GOTERM_MF_DIRECT** | [GTP binding](http://www.ebi.ac.uk/QuickGO/GTerm?id=GO:0005525), |
| **INTERPRO** | [Small GTP-binding protein domain](http://www.ebi.ac.uk/interpro/IEntry?ac=IPR005225), [Small GTPase superfamily, SAR1-type](http://www.ebi.ac.uk/interpro/IEntry?ac=IPR006687), [Small GTPase superfamily, ARF/SAR type](http://www.ebi.ac.uk/interpro/IEntry?ac=IPR006689), [P-loop containing nucleoside triphosphate hydrolase](http://www.ebi.ac.uk/interpro/IEntry?ac=IPR027417), |
| **KEGG_PATHWAY** | [Protein processing in endoplasmic reticulum](https://david.ncifcrf.gov/kegg.jsp?path=gga04141$Protein%20processing%20in%20endoplasmic%20reticulum&termId=550025657&source=kegg), |
| **UP_KEYWORDS** | [Complete proteome](http://www.uniprot.org/keywords/?query=Complete%20proteome), [Endoplasmic reticulum](http://www.uniprot.org/keywords/?query=Endoplasmic%20reticulum), [ER-Golgi transport](http://www.uniprot.org/keywords/?query=ER-Golgi%20transport), [Golgi apparatus](http://www.uniprot.org/keywords/?query=Golgi%20apparatus), [GTP-binding](http://www.uniprot.org/keywords/?query=GTP-binding), [Nucleotide-binding](http://www.uniprot.org/keywords/?query=Nucleotide-binding), [Protein transport](http://www.uniprot.org/keywords/?query=Protein%20transport), [Reference proteome](http://www.uniprot.org/keywords/?query=Reference%20proteome), [Transport](http://www.uniprot.org/keywords/?query=Transport), |
| **SEC24A** | [**SEC24 family member A(SEC24A)**](https://david.ncifcrf.gov/geneReportFull.jsp?rowids=416313) |
| **COG_ONTOLOGY** | [Intracellular trafficking and secretion](http://www.ncbi.nlm.nih.gov/COG/new/), |
| **GOTERM_BP_DIRECT** | [intracellular protein transport](http://www.ebi.ac.uk/QuickGO/GTerm?id=GO:0006886), [ER to Golgi vesicle-mediated transport](http://www.ebi.ac.uk/QuickGO/GTerm?id=GO:0006888), [regulation of low-density lipoprotein particle receptor biosynthetic process](http://www.ebi.ac.uk/QuickGO/GTerm?id=GO:0045714), [positive regulation of protein secretion](http://www.ebi.ac.uk/QuickGO/GTerm?id=GO:0050714), [positive regulation of cholesterol homeostasis](http://www.ebi.ac.uk/QuickGO/GTerm?id=GO:2000189), |
| **GOTERM_CC_DIRECT** | [COPII vesicle coat](http://www.ebi.ac.uk/QuickGO/GTerm?id=GO:0030127), |
| **GOTERM_MF_DIRECT** | [zinc ion binding](http://www.ebi.ac.uk/QuickGO/GTerm?id=GO:0008270), |
| **INTERPRO** | [von Willebrand factor, type A](http://www.ebi.ac.uk/interpro/IEntry?ac=IPR002035), [Zinc finger, Sec23/Sec24-type](http://www.ebi.ac.uk/interpro/IEntry?ac=IPR006895), [Sec23/Sec24, trunk domain](http://www.ebi.ac.uk/interpro/IEntry?ac=IPR006896), [Sec23/Sec24, helical domain](http://www.ebi.ac.uk/interpro/IEntry?ac=IPR006900), [Gelsolin domain](http://www.ebi.ac.uk/interpro/IEntry?ac=IPR007123), [Sec23/Sec24 beta-sandwich](http://www.ebi.ac.uk/interpro/IEntry?ac=IPR012990), |
| **KEGG_PATHWAY** | [Protein processing in endoplasmic reticulum](https://david.ncifcrf.gov/kegg.jsp?path=gga04141$Protein%20processing%20in%20endoplasmic%20reticulum&termId=550025657&source=kegg), |
| **UP_KEYWORDS** | [Complete proteome](http://www.uniprot.org/keywords/?query=Complete%20proteome), [Protein transport](http://www.uniprot.org/keywords/?query=Protein%20transport), [Reference proteome](http://www.uniprot.org/keywords/?query=Reference%20proteome), [Transport](http://www.uniprot.org/keywords/?query=Transport), |
| **UPF3A** | [**UPF3 regulator of nonsense transcripts homolog A (yeast)(UPF3A)**](https://david.ncifcrf.gov/geneReportFull.jsp?rowids=418734) |
| **GOTERM_BP_DIRECT** | [positive regulation of translation](http://www.ebi.ac.uk/QuickGO/GTerm?id=GO:0045727), |
| **GOTERM_CC_DIRECT** | [nucleoplasm](http://www.ebi.ac.uk/QuickGO/GTerm?id=GO:0005654), [cytoplasm](http://www.ebi.ac.uk/QuickGO/GTerm?id=GO:0005737), [plasma membrane](http://www.ebi.ac.uk/QuickGO/GTerm?id=GO:0005886), [exon-exon junction complex](http://www.ebi.ac.uk/QuickGO/GTerm?id=GO:0035145), |
| **GOTERM_MF_DIRECT** | [nucleotide binding](http://www.ebi.ac.uk/QuickGO/GTerm?id=GO:0000166), [telomeric DNA binding](http://www.ebi.ac.uk/QuickGO/GTerm?id=GO:0042162), |
| **INTERPRO** | [Regulator of nonsense-mediated decay, UPF3](http://www.ebi.ac.uk/interpro/IEntry?ac=IPR005120), [Nucleotide-binding, alpha-beta plait](http://www.ebi.ac.uk/interpro/IEntry?ac=IPR012677), |
| **KEGG_PATHWAY** | [RNA transport](https://david.ncifcrf.gov/kegg.jsp?path=gga03013$RNA%20transport&termId=550025627&source=kegg), [mRNA surveillance pathway](https://david.ncifcrf.gov/kegg.jsp?path=gga03015$mRNA%20surveillance%20pathway&termId=550025628&source=kegg), |
| **UP_KEYWORDS** | [Coiled coil](http://www.uniprot.org/keywords/?query=Coiled%20coil), [Complete proteome](http://www.uniprot.org/keywords/?query=Complete%20proteome), [Reference proteome](http://www.uniprot.org/keywords/?query=Reference%20proteome), |
| **ACACA** | [**acetyl-CoA carboxylase alpha(ACACA)**](https://david.ncifcrf.gov/geneReportFull.jsp?rowids=396504) |
| **COG_ONTOLOGY** | [Lipid metabolism](http://www.ncbi.nlm.nih.gov/COG/new/), |
| **GOTERM_BP_DIRECT** | [tissue homeostasis](http://www.ebi.ac.uk/QuickGO/GTerm?id=GO:0001894), [acetyl-CoA metabolic process](http://www.ebi.ac.uk/QuickGO/GTerm?id=GO:0006084), [fatty acid biosynthetic process](http://www.ebi.ac.uk/QuickGO/GTerm?id=GO:0006633), [transport](http://www.ebi.ac.uk/QuickGO/GTerm?id=GO:0006810), [response to carbohydrate](http://www.ebi.ac.uk/QuickGO/GTerm?id=GO:0009743), [positive regulation of gene expression](http://www.ebi.ac.uk/QuickGO/GTerm?id=GO:0010628), [response to insulin](http://www.ebi.ac.uk/QuickGO/GTerm?id=GO:0032868), [regulation of gene expression, epigenetic](http://www.ebi.ac.uk/QuickGO/GTerm?id=GO:0040029), [multicellular organismal protein metabolic process](http://www.ebi.ac.uk/QuickGO/GTerm?id=GO:0044268), [positive regulation of transcription, DNA-templated](http://www.ebi.ac.uk/QuickGO/GTerm?id=GO:0045893), [protein homotetramerization](http://www.ebi.ac.uk/QuickGO/GTerm?id=GO:0051289), [lipid homeostasis](http://www.ebi.ac.uk/QuickGO/GTerm?id=GO:0055088), [regulation of biological quality](http://www.ebi.ac.uk/QuickGO/GTerm?id=GO:0065008), [response to fatty acid](http://www.ebi.ac.uk/QuickGO/GTerm?id=GO:0070542), [cellular response to prostaglandin E stimulus](http://www.ebi.ac.uk/QuickGO/GTerm?id=GO:0071380), [response to thyroid hormone](http://www.ebi.ac.uk/QuickGO/GTerm?id=GO:0097066), [malonyl-CoA biosynthetic process](http://www.ebi.ac.uk/QuickGO/GTerm?id=GO:2001295), |
| **GOTERM_CC_DIRECT** | [cell](http://www.ebi.ac.uk/QuickGO/GTerm?id=GO:0005623), [nucleolus](http://www.ebi.ac.uk/QuickGO/GTerm?id=GO:0005730), [cytoplasm](http://www.ebi.ac.uk/QuickGO/GTerm?id=GO:0005737), [mitochondrion](http://www.ebi.ac.uk/QuickGO/GTerm?id=GO:0005739), [cytosol](http://www.ebi.ac.uk/QuickGO/GTerm?id=GO:0005829), [actin cytoskeleton](http://www.ebi.ac.uk/QuickGO/GTerm?id=GO:0015629), [extracellular exosome](http://www.ebi.ac.uk/QuickGO/GTerm?id=GO:0070062), |
| **GOTERM_MF_DIRECT** | [acetyl-CoA carboxylase activity](http://www.ebi.ac.uk/QuickGO/GTerm?id=GO:0003989), [biotin carboxylase activity](http://www.ebi.ac.uk/QuickGO/GTerm?id=GO:0004075), [receptor binding](http://www.ebi.ac.uk/QuickGO/GTerm?id=GO:0005102), [ATP binding](http://www.ebi.ac.uk/QuickGO/GTerm?id=GO:0005524), [biotin binding](http://www.ebi.ac.uk/QuickGO/GTerm?id=GO:0009374), [sterol response element binding](http://www.ebi.ac.uk/QuickGO/GTerm?id=GO:0032810), [metal ion binding](http://www.ebi.ac.uk/QuickGO/GTerm?id=GO:0046872), [thyroid hormone receptor binding](http://www.ebi.ac.uk/QuickGO/GTerm?id=GO:0046966), [DBD domain binding](http://www.ebi.ac.uk/QuickGO/GTerm?id=GO:0050692), |
| **INTERPRO** | [Carboxyl transferase](http://www.ebi.ac.uk/interpro/IEntry?ac=IPR000022), [Biotin/lipoyl attachment](http://www.ebi.ac.uk/interpro/IEntry?ac=IPR000089), [Biotin-binding site](http://www.ebi.ac.uk/interpro/IEntry?ac=IPR001882), [Carbamoyl-phosphate synthetase large subunit-like, ATP-binding domain](http://www.ebi.ac.uk/interpro/IEntry?ac=IPR005479), [Carbamoyl-phosphate synthase, large subunit, N-terminal](http://www.ebi.ac.uk/interpro/IEntry?ac=IPR005481), [Biotin carboxylase, C-terminal](http://www.ebi.ac.uk/interpro/IEntry?ac=IPR005482), [Single hybrid motif](http://www.ebi.ac.uk/interpro/IEntry?ac=IPR011053), [Rudiment single hybrid motif](http://www.ebi.ac.uk/interpro/IEntry?ac=IPR011054), [ATP-grasp fold](http://www.ebi.ac.uk/interpro/IEntry?ac=IPR011761), [Acetyl-coenzyme A carboxyltransferase, N-terminal](http://www.ebi.ac.uk/interpro/IEntry?ac=IPR011762), [Acetyl-coenzyme A carboxyltransferase, C-terminal](http://www.ebi.ac.uk/interpro/IEntry?ac=IPR011763), [Biotin carboxylation domain](http://www.ebi.ac.uk/interpro/IEntry?ac=IPR011764), [Acetyl-CoA carboxylase, central domain](http://www.ebi.ac.uk/interpro/IEntry?ac=IPR013537), [ATP-grasp fold, subdomain 1](http://www.ebi.ac.uk/interpro/IEntry?ac=IPR013815), [ATP-grasp fold, subdomain 2](http://www.ebi.ac.uk/interpro/IEntry?ac=IPR013816), [Pre-ATP-grasp domain](http://www.ebi.ac.uk/interpro/IEntry?ac=IPR016185), |
| **KEGG_PATHWAY** | [Fatty acid biosynthesis](https://david.ncifcrf.gov/kegg.jsp?path=gga00061$Fatty%20acid%20biosynthesis&termId=550025539&source=kegg), [Pyruvate metabolism](https://david.ncifcrf.gov/kegg.jsp?path=gga00620$Pyruvate%20metabolism&termId=550025594&source=kegg), [Propanoate metabolism](https://david.ncifcrf.gov/kegg.jsp?path=gga00640$Propanoate%20metabolism&termId=550025596&source=kegg), [Metabolic pathways](https://david.ncifcrf.gov/kegg.jsp?path=gga01100$Metabolic%20pathways&termId=550025617&source=kegg), [Fatty acid metabolism](https://david.ncifcrf.gov/kegg.jsp?path=gga01212$Fatty%20acid%20metabolism&termId=550025621&source=kegg), [Insulin signaling pathway](https://david.ncifcrf.gov/kegg.jsp?path=gga04910$Insulin%20signaling%20pathway&termId=550025687&source=kegg), |
| **SMART** | [SM00878](http://smart.embl.de/smart/do_annotation.pl?DOMAIN=SM00878), |
| **UP_KEYWORDS** | [Acetylation](http://www.uniprot.org/keywords/?query=Acetylation), [ATP-binding](http://www.uniprot.org/keywords/?query=ATP-binding), [Biotin](http://www.uniprot.org/keywords/?query=Biotin), [Complete proteome](http://www.uniprot.org/keywords/?query=Complete%20proteome), [Cytoplasm](http://www.uniprot.org/keywords/?query=Cytoplasm), [Direct protein sequencing](http://www.uniprot.org/keywords/?query=Direct%20protein%20sequencing), [Fatty acid biosynthesis](http://www.uniprot.org/keywords/?query=Fatty%20acid%20biosynthesis), [Fatty acid metabolism](http://www.uniprot.org/keywords/?query=Fatty%20acid%20metabolism), [Ligase](http://www.uniprot.org/keywords/?query=Ligase), [Lipid biosynthesis](http://www.uniprot.org/keywords/?query=Lipid%20biosynthesis), [Lipid metabolism](http://www.uniprot.org/keywords/?query=Lipid%20metabolism), [Manganese](http://www.uniprot.org/keywords/?query=Manganese), [Metal-binding](http://www.uniprot.org/keywords/?query=Metal-binding), [Multifunctional enzyme](http://www.uniprot.org/keywords/?query=Multifunctional%20enzyme), [Nucleotide-binding](http://www.uniprot.org/keywords/?query=Nucleotide-binding), [Phosphoprotein](http://www.uniprot.org/keywords/?query=Phosphoprotein), [Reference proteome](http://www.uniprot.org/keywords/?query=Reference%20proteome), |
| **UP_SEQ_FEATURE** | binding site:Coenzyme A, chain:Acetyl-CoA carboxylase, domain:ATP-grasp, domain:Biotin carboxylation, domain:Biotinyl-binding, domain:Carboxyltransferase, metal ion-binding site:Manganese 1, metal ion-binding site:Manganese 2, modified residue, nucleotide phosphate-binding region:ATP, |
| **ADCYAP1** | [**adenylate cyclase activating polypeptide 1 (pituitary)(ADCYAP1)**](https://david.ncifcrf.gov/geneReportFull.jsp?rowids=408251) |
| **GOTERM_BP_DIRECT** | [aggressive behavior](http://www.ebi.ac.uk/QuickGO/GTerm?id=GO:0002118), [positive regulation of cAMP biosynthetic process](http://www.ebi.ac.uk/QuickGO/GTerm?id=GO:0030819), [social behavior](http://www.ebi.ac.uk/QuickGO/GTerm?id=GO:0035176), [locomotion](http://www.ebi.ac.uk/QuickGO/GTerm?id=GO:0040011), [drinking behavior](http://www.ebi.ac.uk/QuickGO/GTerm?id=GO:0042756), [positive regulation of hormone secretion](http://www.ebi.ac.uk/QuickGO/GTerm?id=GO:0046887), |
| **GOTERM_CC_DIRECT** | [extracellular region](http://www.ebi.ac.uk/QuickGO/GTerm?id=GO:0005576), [cell](http://www.ebi.ac.uk/QuickGO/GTerm?id=GO:0005623), [neuron projection](http://www.ebi.ac.uk/QuickGO/GTerm?id=GO:0043005), [perikaryon](http://www.ebi.ac.uk/QuickGO/GTerm?id=GO:0043204), [cell body](http://www.ebi.ac.uk/QuickGO/GTerm?id=GO:0044297), [cell body fiber](http://www.ebi.ac.uk/QuickGO/GTerm?id=GO:0070852), |
| **GOTERM_MF_DIRECT** | [hormone activity](http://www.ebi.ac.uk/QuickGO/GTerm?id=GO:0005179), |
| **INTERPRO** | [Glucagon/GIP/secretin/VIP](http://www.ebi.ac.uk/interpro/IEntry?ac=IPR000532), |
| **SMART** | [GLUCA](http://smart.embl.de/smart/do_annotation.pl?DOMAIN=SM00070), |
| **UP_KEYWORDS** | [Alternative splicing](http://www.uniprot.org/keywords/?query=Alternative%20splicing), [Amidation](http://www.uniprot.org/keywords/?query=Amidation), [Cleavage on pair of basic residues](http://www.uniprot.org/keywords/?query=Cleavage%20on%20pair%20of%20basic%20residues), [Complete proteome](http://www.uniprot.org/keywords/?query=Complete%20proteome), [Direct protein sequencing](http://www.uniprot.org/keywords/?query=Direct%20protein%20sequencing), [Hormone](http://www.uniprot.org/keywords/?query=Hormone), [Reference proteome](http://www.uniprot.org/keywords/?query=Reference%20proteome), [Secreted](http://www.uniprot.org/keywords/?query=Secreted), [Signal](http://www.uniprot.org/keywords/?query=Signal), |
| **UP_SEQ_FEATURE** | modified residue, peptide:Growth hormone-releasing factor 1-46, peptide:Pituitary adenylate cyclase-activating polypeptide 27, peptide:Pituitary adenylate cyclase-activating polypeptide 38, signal peptide, splice variant, |
| **ALB** | [**albumin(ALB)**](https://david.ncifcrf.gov/geneReportFull.jsp?rowids=396197) |
| **GOTERM_BP_DIRECT** | [retina homeostasis](http://www.ebi.ac.uk/QuickGO/GTerm?id=GO:0001895), [transport](http://www.ebi.ac.uk/QuickGO/GTerm?id=GO:0006810), [cellular response to starvation](http://www.ebi.ac.uk/QuickGO/GTerm?id=GO:0009267), [response to virus](http://www.ebi.ac.uk/QuickGO/GTerm?id=GO:0009615), [hemolysis by symbiont of host erythrocytes](http://www.ebi.ac.uk/QuickGO/GTerm?id=GO:0019836), [response to vitamin A](http://www.ebi.ac.uk/QuickGO/GTerm?id=GO:0033189), [negative regulation of apoptotic process](http://www.ebi.ac.uk/QuickGO/GTerm?id=GO:0043066), [maintenance of mitochondrion location](http://www.ebi.ac.uk/QuickGO/GTerm?id=GO:0051659), |
| **GOTERM_CC_DIRECT** | [extracellular space](http://www.ebi.ac.uk/QuickGO/GTerm?id=GO:0005615), [nucleus](http://www.ebi.ac.uk/QuickGO/GTerm?id=GO:0005634), [endoplasmic reticulum](http://www.ebi.ac.uk/QuickGO/GTerm?id=GO:0005783), [Golgi apparatus](http://www.ebi.ac.uk/QuickGO/GTerm?id=GO:0005794), [myelin sheath](http://www.ebi.ac.uk/QuickGO/GTerm?id=GO:0043209), [protein complex](http://www.ebi.ac.uk/QuickGO/GTerm?id=GO:0043234), [yolk](http://www.ebi.ac.uk/QuickGO/GTerm?id=GO:0060417), [extracellular exosome](http://www.ebi.ac.uk/QuickGO/GTerm?id=GO:0070062), [blood microparticle](http://www.ebi.ac.uk/QuickGO/GTerm?id=GO:0072562), |
| **GOTERM_MF_DIRECT** | [DNA binding](http://www.ebi.ac.uk/QuickGO/GTerm?id=GO:0003677), [fatty acid binding](http://www.ebi.ac.uk/QuickGO/GTerm?id=GO:0005504), [drug binding](http://www.ebi.ac.uk/QuickGO/GTerm?id=GO:0008144), [toxic substance binding](http://www.ebi.ac.uk/QuickGO/GTerm?id=GO:0015643), [oxygen binding](http://www.ebi.ac.uk/QuickGO/GTerm?id=GO:0019825), [pyridoxal phosphate binding](http://www.ebi.ac.uk/QuickGO/GTerm?id=GO:0030170), [small molecule binding](http://www.ebi.ac.uk/QuickGO/GTerm?id=GO:0036094), [metal ion binding](http://www.ebi.ac.uk/QuickGO/GTerm?id=GO:0046872), |
| **INTERPRO** | [ALB/AFP/VDB](http://www.ebi.ac.uk/interpro/IEntry?ac=IPR000264), [Serum albumin, N-terminal](http://www.ebi.ac.uk/interpro/IEntry?ac=IPR014760), [Serum albumin, conserved site](http://www.ebi.ac.uk/interpro/IEntry?ac=IPR020857), [Serum albumin-like](http://www.ebi.ac.uk/interpro/IEntry?ac=IPR020858), [Serum albumin/Alpha-fetoprotein](http://www.ebi.ac.uk/interpro/IEntry?ac=IPR021177), |
| **PIR_SUPERFAMILY** | [serum albumin](http://pir.georgetown.edu/cgi-bin/ipcSF?id=PIRSF002520), |
| **SMART** | [ALBUMIN](http://smart.embl.de/smart/do_annotation.pl?DOMAIN=SM00103), |
| **UP_KEYWORDS** | [Allergen](http://www.uniprot.org/keywords/?query=Allergen), [Complete proteome](http://www.uniprot.org/keywords/?query=Complete%20proteome), [Copper](http://www.uniprot.org/keywords/?query=Copper), [Direct protein sequencing](http://www.uniprot.org/keywords/?query=Direct%20protein%20sequencing), [Disulfide bond](http://www.uniprot.org/keywords/?query=Disulfide%20bond), [Glycoprotein](http://www.uniprot.org/keywords/?query=Glycoprotein), [Lipid-binding](http://www.uniprot.org/keywords/?query=Lipid-binding), [Metal-binding](http://www.uniprot.org/keywords/?query=Metal-binding), [Reference proteome](http://www.uniprot.org/keywords/?query=Reference%20proteome), [Repeat](http://www.uniprot.org/keywords/?query=Repeat), [Secreted](http://www.uniprot.org/keywords/?query=Secreted), [Signal](http://www.uniprot.org/keywords/?query=Signal), |
| **UP_SEQ_FEATURE** | chain:Serum albumin, disulfide bond, domain:Albumin 1, domain:Albumin 2, domain:Albumin 3, glycosylation site:N-linked (GlcNAc...), metal ion-binding site:Copper, sequence conflict, signal peptide, |
| **CDH13** | [**cadherin 13(CDH13)**](https://david.ncifcrf.gov/geneReportFull.jsp?rowids=414849) |
| **GOTERM_BP_DIRECT** | [positive regulation of endothelial cell proliferation](http://www.ebi.ac.uk/QuickGO/GTerm?id=GO:0001938), [positive regulation of cell-matrix adhesion](http://www.ebi.ac.uk/QuickGO/GTerm?id=GO:0001954), [sprouting angiogenesis](http://www.ebi.ac.uk/QuickGO/GTerm?id=GO:0002040), [homophilic cell adhesion via plasma membrane adhesion molecules](http://www.ebi.ac.uk/QuickGO/GTerm?id=GO:0007156), [negative regulation of cell adhesion](http://www.ebi.ac.uk/QuickGO/GTerm?id=GO:0007162), [Rho protein signal transduction](http://www.ebi.ac.uk/QuickGO/GTerm?id=GO:0007266), [negative regulation of cell proliferation](http://www.ebi.ac.uk/QuickGO/GTerm?id=GO:0008285), [calcium-dependent cell-cell adhesion via plasma membrane cell adhesion molecules](http://www.ebi.ac.uk/QuickGO/GTerm?id=GO:0016339), [Rac protein signal transduction](http://www.ebi.ac.uk/QuickGO/GTerm?id=GO:0016601), [lamellipodium assembly](http://www.ebi.ac.uk/QuickGO/GTerm?id=GO:0030032), [regulation of endocytosis](http://www.ebi.ac.uk/QuickGO/GTerm?id=GO:0030100), [positive regulation of cell migration](http://www.ebi.ac.uk/QuickGO/GTerm?id=GO:0030335), [regulation of epidermal growth factor receptor signaling pathway](http://www.ebi.ac.uk/QuickGO/GTerm?id=GO:0042058), [endothelial cell migration](http://www.ebi.ac.uk/QuickGO/GTerm?id=GO:0043542), [keratinocyte proliferation](http://www.ebi.ac.uk/QuickGO/GTerm?id=GO:0043616), [positive regulation of transcription from RNA polymerase II promoter](http://www.ebi.ac.uk/QuickGO/GTerm?id=GO:0045944), [positive regulation of smooth muscle cell proliferation](http://www.ebi.ac.uk/QuickGO/GTerm?id=GO:0048661), [positive regulation of calcium-mediated signaling](http://www.ebi.ac.uk/QuickGO/GTerm?id=GO:0050850), [positive regulation of positive chemotaxis](http://www.ebi.ac.uk/QuickGO/GTerm?id=GO:0050927), [localization within membrane](http://www.ebi.ac.uk/QuickGO/GTerm?id=GO:0051668), [low-density lipoprotein particle mediated signaling](http://www.ebi.ac.uk/QuickGO/GTerm?id=GO:0055096), |
| **GOTERM_CC_DIRECT** | [extracellular space](http://www.ebi.ac.uk/QuickGO/GTerm?id=GO:0005615), [cytoplasm](http://www.ebi.ac.uk/QuickGO/GTerm?id=GO:0005737), [plasma membrane](http://www.ebi.ac.uk/QuickGO/GTerm?id=GO:0005886), [caveola](http://www.ebi.ac.uk/QuickGO/GTerm?id=GO:0005901), [focal adhesion](http://www.ebi.ac.uk/QuickGO/GTerm?id=GO:0005925), [external side of plasma membrane](http://www.ebi.ac.uk/QuickGO/GTerm?id=GO:0009897), [cell surface](http://www.ebi.ac.uk/QuickGO/GTerm?id=GO:0009986), [anchored component of membrane](http://www.ebi.ac.uk/QuickGO/GTerm?id=GO:0031225), [neuron projection](http://www.ebi.ac.uk/QuickGO/GTerm?id=GO:0043005), [fibril](http://www.ebi.ac.uk/QuickGO/GTerm?id=GO:0043205), [extracellular exosome](http://www.ebi.ac.uk/QuickGO/GTerm?id=GO:0070062), |
| **GOTERM_MF_DIRECT** | [calcium ion binding](http://www.ebi.ac.uk/QuickGO/GTerm?id=GO:0005509), [low-density lipoprotein particle binding](http://www.ebi.ac.uk/QuickGO/GTerm?id=GO:0030169), [protein homodimerization activity](http://www.ebi.ac.uk/QuickGO/GTerm?id=GO:0042803), |
| **INTERPRO** | [Cadherin](http://www.ebi.ac.uk/interpro/IEntry?ac=IPR002126), [Cadherin prodomain](http://www.ebi.ac.uk/interpro/IEntry?ac=IPR014868), [Cadherin-like](http://www.ebi.ac.uk/interpro/IEntry?ac=IPR015919), [Cadherin conserved site](http://www.ebi.ac.uk/interpro/IEntry?ac=IPR020894), |
| **SMART** | [CA](http://smart.embl.de/smart/do_annotation.pl?DOMAIN=SM00112), [SM01055](http://smart.embl.de/smart/do_annotation.pl?DOMAIN=SM01055), |
| **UP_KEYWORDS** | [3D-structure](http://www.uniprot.org/keywords/?query=3D-structure), [Alternative splicing](http://www.uniprot.org/keywords/?query=Alternative%20splicing), [Calcium](http://www.uniprot.org/keywords/?query=Calcium), [Cell adhesion](http://www.uniprot.org/keywords/?query=Cell%20adhesion), [Cell membrane](http://www.uniprot.org/keywords/?query=Cell%20membrane), [Cleavage on pair of basic residues](http://www.uniprot.org/keywords/?query=Cleavage%20on%20pair%20of%20basic%20residues), [Complete proteome](http://www.uniprot.org/keywords/?query=Complete%20proteome), [Direct protein sequencing](http://www.uniprot.org/keywords/?query=Direct%20protein%20sequencing), [Glycoprotein](http://www.uniprot.org/keywords/?query=Glycoprotein), [GPI-anchor](http://www.uniprot.org/keywords/?query=GPI-anchor), [Lipoprotein](http://www.uniprot.org/keywords/?query=Lipoprotein), [Membrane](http://www.uniprot.org/keywords/?query=Membrane), [Metal-binding](http://www.uniprot.org/keywords/?query=Metal-binding), [Reference proteome](http://www.uniprot.org/keywords/?query=Reference%20proteome), [Repeat](http://www.uniprot.org/keywords/?query=Repeat), [Signal](http://www.uniprot.org/keywords/?query=Signal), |
| **UP_SEQ_FEATURE** | chain:Cadherin-13, domain:Cadherin 1, domain:Cadherin 2, domain:Cadherin 3, domain:Cadherin 4, domain:Cadherin 5, glycosylation site:N-linked (GlcNAc...), lipid moiety-binding region:GPI-anchor amidated aspartate, propeptide:Removed in mature form, signal peptide, splice variant, |
| **CDH2** | [**cadherin 2, type 1, N-cadherin (neuronal)(CDH2)**](https://david.ncifcrf.gov/geneReportFull.jsp?rowids=414745) |
| **GOTERM_BP_DIRECT** | [cartilage condensation](http://www.ebi.ac.uk/QuickGO/GTerm?id=GO:0001502), [neural tube formation](http://www.ebi.ac.uk/QuickGO/GTerm?id=GO:0001841), [cell adhesion](http://www.ebi.ac.uk/QuickGO/GTerm?id=GO:0007155), [homophilic cell adhesion via plasma membrane adhesion molecules](http://www.ebi.ac.uk/QuickGO/GTerm?id=GO:0007156), [heterophilic cell-cell adhesion via plasma membrane cell adhesion molecules](http://www.ebi.ac.uk/QuickGO/GTerm?id=GO:0007157), [glial cell differentiation](http://www.ebi.ac.uk/QuickGO/GTerm?id=GO:0010001), [calcium-dependent cell-cell adhesion via plasma membrane cell adhesion molecules](http://www.ebi.ac.uk/QuickGO/GTerm?id=GO:0016339), [cell migration](http://www.ebi.ac.uk/QuickGO/GTerm?id=GO:0016477), [cerebral cortex development](http://www.ebi.ac.uk/QuickGO/GTerm?id=GO:0021987), [negative regulation of cell migration](http://www.ebi.ac.uk/QuickGO/GTerm?id=GO:0030336), [neural crest cell delamination](http://www.ebi.ac.uk/QuickGO/GTerm?id=GO:0036032), [positive regulation of MAPK cascade](http://www.ebi.ac.uk/QuickGO/GTerm?id=GO:0043410), [cell-cell adhesion mediated by cadherin](http://www.ebi.ac.uk/QuickGO/GTerm?id=GO:0044331), [blood vessel morphogenesis](http://www.ebi.ac.uk/QuickGO/GTerm?id=GO:0048514), [brain morphogenesis](http://www.ebi.ac.uk/QuickGO/GTerm?id=GO:0048854), [homeostasis of number of cells](http://www.ebi.ac.uk/QuickGO/GTerm?id=GO:0048872), [striated muscle cell differentiation](http://www.ebi.ac.uk/QuickGO/GTerm?id=GO:0051146), [radial glial cell differentiation](http://www.ebi.ac.uk/QuickGO/GTerm?id=GO:0060019), [limb development](http://www.ebi.ac.uk/QuickGO/GTerm?id=GO:0060173), [neuroepithelial cell differentiation](http://www.ebi.ac.uk/QuickGO/GTerm?id=GO:0060563), [trigeminal ganglion formation](http://www.ebi.ac.uk/QuickGO/GTerm?id=GO:0061561), [trigeminal ganglion structural organization](http://www.ebi.ac.uk/QuickGO/GTerm?id=GO:0061563), [regulation of oligodendrocyte progenitor proliferation](http://www.ebi.ac.uk/QuickGO/GTerm?id=GO:0070445), [establishment of protein localization to plasma membrane](http://www.ebi.ac.uk/QuickGO/GTerm?id=GO:0090002), [negative regulation of canonical Wnt signaling pathway](http://www.ebi.ac.uk/QuickGO/GTerm?id=GO:0090090), [neuroligin clustering involved in postsynaptic membrane assembly](http://www.ebi.ac.uk/QuickGO/GTerm?id=GO:0097118), [neuronal stem cell population maintenance](http://www.ebi.ac.uk/QuickGO/GTerm?id=GO:0097150), [cell-cell adhesion](http://www.ebi.ac.uk/QuickGO/GTerm?id=GO:0098609), [cell aggregation](http://www.ebi.ac.uk/QuickGO/GTerm?id=GO:0098743), [regulation of postsynaptic density protein 95 clustering](http://www.ebi.ac.uk/QuickGO/GTerm?id=GO:1902897), [positive regulation of synaptic vesicle clustering](http://www.ebi.ac.uk/QuickGO/GTerm?id=GO:2000809), |
| **GOTERM_CC_DIRECT** | [cell](http://www.ebi.ac.uk/QuickGO/GTerm?id=GO:0005623), [cytosol](http://www.ebi.ac.uk/QuickGO/GTerm?id=GO:0005829), [plasma membrane](http://www.ebi.ac.uk/QuickGO/GTerm?id=GO:0005886), [cell-cell junction](http://www.ebi.ac.uk/QuickGO/GTerm?id=GO:0005911), [adherens junction](http://www.ebi.ac.uk/QuickGO/GTerm?id=GO:0005912), [cell-cell adherens junction](http://www.ebi.ac.uk/QuickGO/GTerm?id=GO:0005913), [fascia adherens](http://www.ebi.ac.uk/QuickGO/GTerm?id=GO:0005916), [focal adhesion](http://www.ebi.ac.uk/QuickGO/GTerm?id=GO:0005925), [cell surface](http://www.ebi.ac.uk/QuickGO/GTerm?id=GO:0009986), [intercalated disc](http://www.ebi.ac.uk/QuickGO/GTerm?id=GO:0014704), [integral component of membrane](http://www.ebi.ac.uk/QuickGO/GTerm?id=GO:0016021), [basolateral plasma membrane](http://www.ebi.ac.uk/QuickGO/GTerm?id=GO:0016323), [apical plasma membrane](http://www.ebi.ac.uk/QuickGO/GTerm?id=GO:0016324), [catenin complex](http://www.ebi.ac.uk/QuickGO/GTerm?id=GO:0016342), [lamellipodium](http://www.ebi.ac.uk/QuickGO/GTerm?id=GO:0030027), [cortical actin cytoskeleton](http://www.ebi.ac.uk/QuickGO/GTerm?id=GO:0030864), [neuron projection](http://www.ebi.ac.uk/QuickGO/GTerm?id=GO:0043005), [cell body](http://www.ebi.ac.uk/QuickGO/GTerm?id=GO:0044297), [plasma membrane raft](http://www.ebi.ac.uk/QuickGO/GTerm?id=GO:0044853), [apical part of cell](http://www.ebi.ac.uk/QuickGO/GTerm?id=GO:0045177), [synapse](http://www.ebi.ac.uk/QuickGO/GTerm?id=GO:0045202), [extracellular exosome](http://www.ebi.ac.uk/QuickGO/GTerm?id=GO:0070062), |
| **GOTERM_MF_DIRECT** | [calcium ion binding](http://www.ebi.ac.uk/QuickGO/GTerm?id=GO:0005509), [protein binding](http://www.ebi.ac.uk/QuickGO/GTerm?id=GO:0005515), [protein phosphatase binding](http://www.ebi.ac.uk/QuickGO/GTerm?id=GO:0019903), [alpha-catenin binding](http://www.ebi.ac.uk/QuickGO/GTerm?id=GO:0045294), [gamma-catenin binding](http://www.ebi.ac.uk/QuickGO/GTerm?id=GO:0045295), [cadherin binding](http://www.ebi.ac.uk/QuickGO/GTerm?id=GO:0045296), [extracellular matrix binding](http://www.ebi.ac.uk/QuickGO/GTerm?id=GO:0050840), |
| **INTERPRO** | [Cadherin, cytoplasmic domain](http://www.ebi.ac.uk/interpro/IEntry?ac=IPR000233), [Cadherin](http://www.ebi.ac.uk/interpro/IEntry?ac=IPR002126), [Cadherin prodomain](http://www.ebi.ac.uk/interpro/IEntry?ac=IPR014868), [Cadherin-like](http://www.ebi.ac.uk/interpro/IEntry?ac=IPR015919), [Cadherin conserved site](http://www.ebi.ac.uk/interpro/IEntry?ac=IPR020894), [Catenin binding domain](http://www.ebi.ac.uk/interpro/IEntry?ac=IPR027397), |
| **KEGG_PATHWAY** | [Cell adhesion molecules (CAMs)](https://david.ncifcrf.gov/kegg.jsp?path=gga04514$Cell%20adhesion%20molecules%20(CAMs)&termId=550025675&source=kegg), |
| **SMART** | [CA](http://smart.embl.de/smart/do_annotation.pl?DOMAIN=SM00112), [SM01055](http://smart.embl.de/smart/do_annotation.pl?DOMAIN=SM01055), |
| **UP_KEYWORDS** | [Calcium](http://www.uniprot.org/keywords/?query=Calcium), [Cell adhesion](http://www.uniprot.org/keywords/?query=Cell%20adhesion), [Cell membrane](http://www.uniprot.org/keywords/?query=Cell%20membrane), [Cleavage on pair of basic residues](http://www.uniprot.org/keywords/?query=Cleavage%20on%20pair%20of%20basic%20residues), [Complete proteome](http://www.uniprot.org/keywords/?query=Complete%20proteome), [Glycoprotein](http://www.uniprot.org/keywords/?query=Glycoprotein), [Membrane](http://www.uniprot.org/keywords/?query=Membrane), [Metal-binding](http://www.uniprot.org/keywords/?query=Metal-binding), [Reference proteome](http://www.uniprot.org/keywords/?query=Reference%20proteome), [Repeat](http://www.uniprot.org/keywords/?query=Repeat), [Signal](http://www.uniprot.org/keywords/?query=Signal), [Transmembrane](http://www.uniprot.org/keywords/?query=Transmembrane), [Transmembrane helix](http://www.uniprot.org/keywords/?query=Transmembrane%20helix), |
| **UP_SEQ_FEATURE** | chain:Cadherin-2, compositionally biased region:Ser-rich, domain:Cadherin 1, domain:Cadherin 2, domain:Cadherin 3, domain:Cadherin 4, domain:Cadherin 5, glycosylation site:N-linked (GlcNAc...), sequence conflict, signal peptide, topological domain:Cytoplasmic, topological domain:Extracellular, transmembrane region, |
| **CALB1** | [**calbindin 1, 28kDa(CALB1)**](https://david.ncifcrf.gov/geneReportFull.jsp?rowids=396519) |
| **GOTERM_BP_DIRECT** | [calcium ion transport](http://www.ebi.ac.uk/QuickGO/GTerm?id=GO:0006816), [cellular calcium ion homeostasis](http://www.ebi.ac.uk/QuickGO/GTerm?id=GO:0006874), [short-term memory](http://www.ebi.ac.uk/QuickGO/GTerm?id=GO:0007614), [long-term memory](http://www.ebi.ac.uk/QuickGO/GTerm?id=GO:0007616), [locomotory behavior](http://www.ebi.ac.uk/QuickGO/GTerm?id=GO:0007626), [retina layer formation](http://www.ebi.ac.uk/QuickGO/GTerm?id=GO:0010842), [sensory perception of pain](http://www.ebi.ac.uk/QuickGO/GTerm?id=GO:0019233), [metanephric part of ureteric bud development](http://www.ebi.ac.uk/QuickGO/GTerm?id=GO:0035502), [regulation of cytosolic calcium ion concentration](http://www.ebi.ac.uk/QuickGO/GTerm?id=GO:0051480), [cellular response to organic substance](http://www.ebi.ac.uk/QuickGO/GTerm?id=GO:0071310), [metanephric collecting duct development](http://www.ebi.ac.uk/QuickGO/GTerm?id=GO:0072205), [metanephric distal convoluted tubule development](http://www.ebi.ac.uk/QuickGO/GTerm?id=GO:0072221), [metanephric connecting tubule development](http://www.ebi.ac.uk/QuickGO/GTerm?id=GO:0072286), |
| **GOTERM_CC_DIRECT** | [nucleus](http://www.ebi.ac.uk/QuickGO/GTerm?id=GO:0005634), [cytosol](http://www.ebi.ac.uk/QuickGO/GTerm?id=GO:0005829), [vesicle membrane](http://www.ebi.ac.uk/QuickGO/GTerm?id=GO:0012506), [axon](http://www.ebi.ac.uk/QuickGO/GTerm?id=GO:0030424), [dendrite](http://www.ebi.ac.uk/QuickGO/GTerm?id=GO:0030425), [neuron projection](http://www.ebi.ac.uk/QuickGO/GTerm?id=GO:0043005), [neuronal cell body](http://www.ebi.ac.uk/QuickGO/GTerm?id=GO:0043025), [postsynaptic membrane](http://www.ebi.ac.uk/QuickGO/GTerm?id=GO:0045211), [extracellular exosome](http://www.ebi.ac.uk/QuickGO/GTerm?id=GO:0070062), |
| **GOTERM_MF_DIRECT** | [vitamin D binding](http://www.ebi.ac.uk/QuickGO/GTerm?id=GO:0005499), [calcium ion binding](http://www.ebi.ac.uk/QuickGO/GTerm?id=GO:0005509), [zinc ion binding](http://www.ebi.ac.uk/QuickGO/GTerm?id=GO:0008270), |
| **INTERPRO** | [EF-hand domain](http://www.ebi.ac.uk/interpro/IEntry?ac=IPR002048), [EF-hand-like domain](http://www.ebi.ac.uk/interpro/IEntry?ac=IPR011992), [EF-Hand 1, calcium-binding site](http://www.ebi.ac.uk/interpro/IEntry?ac=IPR018247), |
| **SMART** | [EFh](http://smart.embl.de/smart/do_annotation.pl?DOMAIN=SM00054), |
| **UP_KEYWORDS** | [Acetylation](http://www.uniprot.org/keywords/?query=Acetylation), [Calcium](http://www.uniprot.org/keywords/?query=Calcium), [Complete proteome](http://www.uniprot.org/keywords/?query=Complete%20proteome), [Direct protein sequencing](http://www.uniprot.org/keywords/?query=Direct%20protein%20sequencing), [Metal-binding](http://www.uniprot.org/keywords/?query=Metal-binding), [Reference proteome](http://www.uniprot.org/keywords/?query=Reference%20proteome), [Repeat](http://www.uniprot.org/keywords/?query=Repeat), [Vitamin D](http://www.uniprot.org/keywords/?query=Vitamin%20D), |
| **UP_SEQ_FEATURE** | calcium-binding region:1, calcium-binding region:2, calcium-binding region:3, calcium-binding region:4, chain:Calbindin, domain:EF-hand 1, domain:EF-hand 2, domain:EF-hand 3, domain:EF-hand 4, domain:EF-hand 5, modified residue, |
| **CAMKK1** | [**calcium/calmodulin-dependent protein kinase kinase 1, alpha(CAMKK1)**](https://david.ncifcrf.gov/geneReportFull.jsp?rowids=771003) |
| **GOTERM_MF_DIRECT** | [protein serine/threonine kinase activity](http://www.ebi.ac.uk/QuickGO/GTerm?id=GO:0004674), [ATP binding](http://www.ebi.ac.uk/QuickGO/GTerm?id=GO:0005524), |
| **INTERPRO** | [Protein kinase, catalytic domain](http://www.ebi.ac.uk/interpro/IEntry?ac=IPR000719), [Serine/threonine-protein kinase, active site](http://www.ebi.ac.uk/interpro/IEntry?ac=IPR008271), [Protein kinase-like domain](http://www.ebi.ac.uk/interpro/IEntry?ac=IPR011009), [Protein kinase, ATP binding site](http://www.ebi.ac.uk/interpro/IEntry?ac=IPR017441), [Calcium/calmodulin-dependent/calcium-dependent protein kinase](http://www.ebi.ac.uk/interpro/IEntry?ac=IPR020636), |
| **KEGG_PATHWAY** | [Adipocytokine signaling pathway](https://david.ncifcrf.gov/kegg.jsp?path=gga04920$Adipocytokine%20signaling%20pathway&termId=550025691&source=kegg), |
| **SMART** | [S_TKc](http://smart.embl.de/smart/do_annotation.pl?DOMAIN=SM00220), |
| **UP_KEYWORDS** | [ATP-binding](http://www.uniprot.org/keywords/?query=ATP-binding), [Complete proteome](http://www.uniprot.org/keywords/?query=Complete%20proteome), [Kinase](http://www.uniprot.org/keywords/?query=Kinase), [Nucleotide-binding](http://www.uniprot.org/keywords/?query=Nucleotide-binding), [Reference proteome](http://www.uniprot.org/keywords/?query=Reference%20proteome), [Serine/threonine-protein kinase](http://www.uniprot.org/keywords/?query=Serine/threonine-protein%20kinase), [Transferase](http://www.uniprot.org/keywords/?query=Transferase), |
| **CAMSAP1** | [**calmodulin regulated spectrin associated protein 1(CAMSAP1)**](https://david.ncifcrf.gov/geneReportFull.jsp?rowids=417127) |
| **GOTERM_BP_DIRECT** | [cytoskeleton organization](http://www.ebi.ac.uk/QuickGO/GTerm?id=GO:0007010), [regulation of cell morphogenesis](http://www.ebi.ac.uk/QuickGO/GTerm?id=GO:0022604), [neuron projection development](http://www.ebi.ac.uk/QuickGO/GTerm?id=GO:0031175), |
| **GOTERM_CC_DIRECT** | [microtubule](http://www.ebi.ac.uk/QuickGO/GTerm?id=GO:0005874), |
| **INTERPRO** | [Calponin homology domain](http://www.ebi.ac.uk/interpro/IEntry?ac=IPR001715), [PRC-barrel-like](http://www.ebi.ac.uk/interpro/IEntry?ac=IPR011033), [CKK domain](http://www.ebi.ac.uk/interpro/IEntry?ac=IPR014797), [Calmodulin-regulated spectrin-associated protein, CH domain](http://www.ebi.ac.uk/interpro/IEntry?ac=IPR022613), |
| **SMART** | [SM01051](http://smart.embl.de/smart/do_annotation.pl?DOMAIN=SM01051), |
| **UP_KEYWORDS** | [Coiled coil](http://www.uniprot.org/keywords/?query=Coiled%20coil), [Complete proteome](http://www.uniprot.org/keywords/?query=Complete%20proteome), [Reference proteome](http://www.uniprot.org/keywords/?query=Reference%20proteome), |
| **CA4** | [**carbonic anhydrase IV(CA4)**](https://david.ncifcrf.gov/geneReportFull.jsp?rowids=417647) |
| **GOTERM_BP_DIRECT** | [bicarbonate transport](http://www.ebi.ac.uk/QuickGO/GTerm?id=GO:0015701), |
| **GOTERM_CC_DIRECT** | [rough endoplasmic reticulum](http://www.ebi.ac.uk/QuickGO/GTerm?id=GO:0005791), [endoplasmic reticulum-Golgi intermediate compartment](http://www.ebi.ac.uk/QuickGO/GTerm?id=GO:0005793), [trans-Golgi network](http://www.ebi.ac.uk/QuickGO/GTerm?id=GO:0005802), [apical plasma membrane](http://www.ebi.ac.uk/QuickGO/GTerm?id=GO:0016324), [transport vesicle membrane](http://www.ebi.ac.uk/QuickGO/GTerm?id=GO:0030658), [secretory granule membrane](http://www.ebi.ac.uk/QuickGO/GTerm?id=GO:0030667), [anchored component of external side of plasma membrane](http://www.ebi.ac.uk/QuickGO/GTerm?id=GO:0031362), [brush border membrane](http://www.ebi.ac.uk/QuickGO/GTerm?id=GO:0031526), [perinuclear region of cytoplasm](http://www.ebi.ac.uk/QuickGO/GTerm?id=GO:0048471), [extracellular exosome](http://www.ebi.ac.uk/QuickGO/GTerm?id=GO:0070062), |
| **GOTERM_MF_DIRECT** | [carbonate dehydratase activity](http://www.ebi.ac.uk/QuickGO/GTerm?id=GO:0004089), [zinc ion binding](http://www.ebi.ac.uk/QuickGO/GTerm?id=GO:0008270), |
| **INTERPRO** | [Alpha carbonic anhydrase](http://www.ebi.ac.uk/interpro/IEntry?ac=IPR001148), [Carbonic anhydrase, CA-IV](http://www.ebi.ac.uk/interpro/IEntry?ac=IPR018343), [Carbonic anhydrase, alpha-class](http://www.ebi.ac.uk/interpro/IEntry?ac=IPR023561), |
| **KEGG_PATHWAY** | [Nitrogen metabolism](https://david.ncifcrf.gov/kegg.jsp?path=gga00910$Nitrogen%20metabolism&termId=550025610&source=kegg), |
| **SMART** | [SM01057](http://smart.embl.de/smart/do_annotation.pl?DOMAIN=SM01057), |
| **UP_KEYWORDS** | [Complete proteome](http://www.uniprot.org/keywords/?query=Complete%20proteome), [Reference proteome](http://www.uniprot.org/keywords/?query=Reference%20proteome), |
| **CDC16** | [**cell division cycle 16(CDC16)**](https://david.ncifcrf.gov/geneReportFull.jsp?rowids=418735) |
| **GOTERM_BP_DIRECT** | [protein K11-linked ubiquitination](http://www.ebi.ac.uk/QuickGO/GTerm?id=GO:0070979), |
| **GOTERM_CC_DIRECT** | [anaphase-promoting complex](http://www.ebi.ac.uk/QuickGO/GTerm?id=GO:0005680), [cytoplasm](http://www.ebi.ac.uk/QuickGO/GTerm?id=GO:0005737), [centrosome](http://www.ebi.ac.uk/QuickGO/GTerm?id=GO:0005813), [spindle microtubule](http://www.ebi.ac.uk/QuickGO/GTerm?id=GO:0005876), |
| **INTERPRO** | [Tetratricopeptide-like helical](http://www.ebi.ac.uk/interpro/IEntry?ac=IPR011990), [Tetratricopeptide repeat-containing domain](http://www.ebi.ac.uk/interpro/IEntry?ac=IPR013026), [Tetratricopeptide repeat](http://www.ebi.ac.uk/interpro/IEntry?ac=IPR019734), |
| **KEGG_PATHWAY** | [Cell cycle](https://david.ncifcrf.gov/kegg.jsp?path=gga04110$Cell%20cycle&termId=550025650&source=kegg), [Oocyte meiosis](https://david.ncifcrf.gov/kegg.jsp?path=gga04114$Oocyte%20meiosis&termId=550025651&source=kegg), [Ubiquitin mediated proteolysis](https://david.ncifcrf.gov/kegg.jsp?path=gga04120$Ubiquitin%20mediated%20proteolysis&termId=550025653&source=kegg), [Progesterone-mediated oocyte maturation](https://david.ncifcrf.gov/kegg.jsp?path=gga04914$Progesterone-mediated%20oocyte%20maturation&termId=550025689&source=kegg), |
| **SMART** | [TPR](http://smart.embl.de/smart/do_annotation.pl?DOMAIN=SM00028), |
| **UP_KEYWORDS** | [Complete proteome](http://www.uniprot.org/keywords/?query=Complete%20proteome), [Reference proteome](http://www.uniprot.org/keywords/?query=Reference%20proteome), |
| **C1H21ORF91** | [**chromosome 1 open reading frame, human C21orf91(C1H21ORF91)**](https://david.ncifcrf.gov/geneReportFull.jsp?rowids=395489) |
| **INTERPRO** | [EURL protein](http://www.ebi.ac.uk/interpro/IEntry?ac=IPR009704), |
| **UP_KEYWORDS** | [Coiled coil](http://www.uniprot.org/keywords/?query=Coiled%20coil), [Complete proteome](http://www.uniprot.org/keywords/?query=Complete%20proteome), [Reference proteome](http://www.uniprot.org/keywords/?query=Reference%20proteome), |
| **UP_SEQ_FEATURE** | chain:Protein EURL, |
| **CHAMP1** | [**chromosome alignment maintaining phosphoprotein 1(CHAMP1)**](https://david.ncifcrf.gov/geneReportFull.jsp?rowids=418733) |
| **GOTERM_BP_DIRECT** | [sister chromatid biorientation](http://www.ebi.ac.uk/QuickGO/GTerm?id=GO:0031134), [protein localization to kinetochore](http://www.ebi.ac.uk/QuickGO/GTerm?id=GO:0034501), [protein localization to microtubule](http://www.ebi.ac.uk/QuickGO/GTerm?id=GO:0035372), [attachment of mitotic spindle microtubules to kinetochore](http://www.ebi.ac.uk/QuickGO/GTerm?id=GO:0051315), |
| **GOTERM_CC_DIRECT** | [condensed chromosome kinetochore](http://www.ebi.ac.uk/QuickGO/GTerm?id=GO:0000777), [nucleoplasm](http://www.ebi.ac.uk/QuickGO/GTerm?id=GO:0005654), [spindle](http://www.ebi.ac.uk/QuickGO/GTerm?id=GO:0005819), |
| **GOTERM_MF_DIRECT** | [nucleic acid binding](http://www.ebi.ac.uk/QuickGO/GTerm?id=GO:0003676), [metal ion binding](http://www.ebi.ac.uk/QuickGO/GTerm?id=GO:0046872), |
| **INTERPRO** | [Zinc finger, C2H2](http://www.ebi.ac.uk/interpro/IEntry?ac=IPR007087), [Zinc finger C2H2-type/integrase DNA-binding domain](http://www.ebi.ac.uk/interpro/IEntry?ac=IPR013087), [Zinc finger, C2H2-like](http://www.ebi.ac.uk/interpro/IEntry?ac=IPR015880), |
| **SMART** | [ZnF_C2H2](http://smart.embl.de/smart/do_annotation.pl?DOMAIN=SM00355), |
| **UP_KEYWORDS** | [Complete proteome](http://www.uniprot.org/keywords/?query=Complete%20proteome), [Reference proteome](http://www.uniprot.org/keywords/?query=Reference%20proteome), |
| **C1QBP** | [**complement component 1, q subcomponent binding protein(C1QBP)**](https://david.ncifcrf.gov/geneReportFull.jsp?rowids=395538) |
| **KEGG_PATHWAY** | [Herpes simplex infection](https://david.ncifcrf.gov/kegg.jsp?path=gga05168$Herpes%20simplex%20infection&termId=550025695&source=kegg), |
| **CRIP1** | [**cysteine rich protein 1(CRIP1)**](https://david.ncifcrf.gov/geneReportFull.jsp?rowids=101747696) |
| **GOTERM_BP_DIRECT** | [intrinsic apoptotic signaling pathway in response to DNA damage](http://www.ebi.ac.uk/QuickGO/GTerm?id=GO:0008630), [response to zinc ion](http://www.ebi.ac.uk/QuickGO/GTerm?id=GO:0010043), [cellular response to antibiotic](http://www.ebi.ac.uk/QuickGO/GTerm?id=GO:0071236), [cellular response to UV-B](http://www.ebi.ac.uk/QuickGO/GTerm?id=GO:0071493), |
| **GOTERM_CC_DIRECT** | [cytoplasm](http://www.ebi.ac.uk/QuickGO/GTerm?id=GO:0005737), |
| **GOTERM_MF_DIRECT** | [AT DNA binding](http://www.ebi.ac.uk/QuickGO/GTerm?id=GO:0003680), [zinc ion binding](http://www.ebi.ac.uk/QuickGO/GTerm?id=GO:0008270), [DNA binding, bending](http://www.ebi.ac.uk/QuickGO/GTerm?id=GO:0008301), [peptide binding](http://www.ebi.ac.uk/QuickGO/GTerm?id=GO:0042277), |
| **INTERPRO** | [Zinc finger, LIM-type](http://www.ebi.ac.uk/interpro/IEntry?ac=IPR001781), |
| **SMART** | [LIM](http://smart.embl.de/smart/do_annotation.pl?DOMAIN=SM00132), |
| **UP_KEYWORDS** | [Complete proteome](http://www.uniprot.org/keywords/?query=Complete%20proteome), [LIM domain](http://www.uniprot.org/keywords/?query=LIM%20domain), [Metal-binding](http://www.uniprot.org/keywords/?query=Metal-binding), [Reference proteome](http://www.uniprot.org/keywords/?query=Reference%20proteome), [Zinc](http://www.uniprot.org/keywords/?query=Zinc), |
| **DHRSX** | [**dehydrogenase/reductase (SDR family) X-linked(DHRSX)**](https://david.ncifcrf.gov/geneReportFull.jsp?rowids=428007) |
| **COG_ONTOLOGY** | [Secondary metabolites biosynthesis, transport, and catabolism / General function prediction only](http://www.ncbi.nlm.nih.gov/COG/new/), |
| **DHRS11** | [**dehydrogenase/reductase (SDR family) member 11(DHRS11)**](https://david.ncifcrf.gov/geneReportFull.jsp?rowids=395172) |
| **GOTERM_BP_DIRECT** | [oxidation-reduction process](http://www.ebi.ac.uk/QuickGO/GTerm?id=GO:0055114), |
| **GOTERM_CC_DIRECT** | [extracellular region](http://www.ebi.ac.uk/QuickGO/GTerm?id=GO:0005576), |
| **GOTERM_MF_DIRECT** | [oxidoreductase activity](http://www.ebi.ac.uk/QuickGO/GTerm?id=GO:0016491), |
| **INTERPRO** | [Glucose/ribitol dehydrogenase](http://www.ebi.ac.uk/interpro/IEntry?ac=IPR002347), [NAD(P)-binding domain](http://www.ebi.ac.uk/interpro/IEntry?ac=IPR016040), [Short-chain dehydrogenase/reductase, conserved site](http://www.ebi.ac.uk/interpro/IEntry?ac=IPR020904), |
| **UP_KEYWORDS** | [Complete proteome](http://www.uniprot.org/keywords/?query=Complete%20proteome), [NADP](http://www.uniprot.org/keywords/?query=NADP), [Oxidoreductase](http://www.uniprot.org/keywords/?query=Oxidoreductase), [Reference proteome](http://www.uniprot.org/keywords/?query=Reference%20proteome), [Secreted](http://www.uniprot.org/keywords/?query=Secreted), [Signal](http://www.uniprot.org/keywords/?query=Signal), |
| **UP_SEQ_FEATURE** | binding site:Acetate, chain:Dehydrogenase/reductase SDR family member 11, nucleotide phosphate-binding region:NADP, signal peptide, |
| **DYRK3** | [**dual-specificity tyrosine-(Y)-phosphorylation regulated kinase 3(DYRK3)**](https://david.ncifcrf.gov/geneReportFull.jsp?rowids=419846) |
| **GOTERM_BP_DIRECT** | [erythrocyte differentiation](http://www.ebi.ac.uk/QuickGO/GTerm?id=GO:0030218), |
| **GOTERM_CC_DIRECT** | [nucleus](http://www.ebi.ac.uk/QuickGO/GTerm?id=GO:0005634), |
| **GOTERM_MF_DIRECT** | [magnesium ion binding](http://www.ebi.ac.uk/QuickGO/GTerm?id=GO:0000287), [protein kinase activity](http://www.ebi.ac.uk/QuickGO/GTerm?id=GO:0004672), [ATP binding](http://www.ebi.ac.uk/QuickGO/GTerm?id=GO:0005524), |
| **INTERPRO** | [Protein kinase, catalytic domain](http://www.ebi.ac.uk/interpro/IEntry?ac=IPR000719), [Serine/threonine-protein kinase, active site](http://www.ebi.ac.uk/interpro/IEntry?ac=IPR008271), [Protein kinase-like domain](http://www.ebi.ac.uk/interpro/IEntry?ac=IPR011009), [Protein kinase, ATP binding site](http://www.ebi.ac.uk/interpro/IEntry?ac=IPR017441), |
| **SMART** | [S_TKc](http://smart.embl.de/smart/do_annotation.pl?DOMAIN=SM00220), |
| **UP_KEYWORDS** | [Complete proteome](http://www.uniprot.org/keywords/?query=Complete%20proteome), [Reference proteome](http://www.uniprot.org/keywords/?query=Reference%20proteome), |
| **EIF2D** | [**eukaryotic translation initiation factor 2D(EIF2D)**](https://david.ncifcrf.gov/geneReportFull.jsp?rowids=419845) |
| **COG_ONTOLOGY** | [Translation, ribosomal structure and biogenesis](http://www.ncbi.nlm.nih.gov/COG/new/), |
| **GOTERM_BP_DIRECT** | [formation of translation preinitiation complex](http://www.ebi.ac.uk/QuickGO/GTerm?id=GO:0001731), [IRES-dependent translational initiation](http://www.ebi.ac.uk/QuickGO/GTerm?id=GO:0002192), [ribosome disassembly](http://www.ebi.ac.uk/QuickGO/GTerm?id=GO:0032790), |
| **GOTERM_CC_DIRECT** | [nucleus](http://www.ebi.ac.uk/QuickGO/GTerm?id=GO:0005634), [cytosolic small ribosomal subunit](http://www.ebi.ac.uk/QuickGO/GTerm?id=GO:0022627), |
| **GOTERM_MF_DIRECT** | [RNA binding](http://www.ebi.ac.uk/QuickGO/GTerm?id=GO:0003723), [translation initiation factor activity](http://www.ebi.ac.uk/QuickGO/GTerm?id=GO:0003743), |
| **INTERPRO** | [Translation initiation factor SUI1](http://www.ebi.ac.uk/interpro/IEntry?ac=IPR001950), [Pseudouridine synthase/archaeosine transglycosylase](http://www.ebi.ac.uk/interpro/IEntry?ac=IPR002478), [SWIB/MDM2 domain](http://www.ebi.ac.uk/interpro/IEntry?ac=IPR003121), [PUA-like domain](http://www.ebi.ac.uk/interpro/IEntry?ac=IPR015947), |
| **UP_KEYWORDS** | [Complete proteome](http://www.uniprot.org/keywords/?query=Complete%20proteome), [Reference proteome](http://www.uniprot.org/keywords/?query=Reference%20proteome), |
| **FGF2** | [**fibroblast growth factor 2 (basic)(FGF2)**](https://david.ncifcrf.gov/geneReportFull.jsp?rowids=396413) |
| **GOTERM_BP_DIRECT** | [activation of MAPKK activity](http://www.ebi.ac.uk/QuickGO/GTerm?id=GO:0000186), [angiogenesis](http://www.ebi.ac.uk/QuickGO/GTerm?id=GO:0001525), [mesoderm formation](http://www.ebi.ac.uk/QuickGO/GTerm?id=GO:0001707), [organ induction](http://www.ebi.ac.uk/QuickGO/GTerm?id=GO:0001759), [tissue homeostasis](http://www.ebi.ac.uk/QuickGO/GTerm?id=GO:0001894), [positive regulation of endothelial cell proliferation](http://www.ebi.ac.uk/QuickGO/GTerm?id=GO:0001938), [lens morphogenesis in camera-type eye](http://www.ebi.ac.uk/QuickGO/GTerm?id=GO:0002089), [epicardial cell to mesenchymal cell transition](http://www.ebi.ac.uk/QuickGO/GTerm?id=GO:0003347), [neural retina development](http://www.ebi.ac.uk/QuickGO/GTerm?id=GO:0003407), [brain development](http://www.ebi.ac.uk/QuickGO/GTerm?id=GO:0007420), [cell proliferation](http://www.ebi.ac.uk/QuickGO/GTerm?id=GO:0008283), [negative regulation of cell proliferation](http://www.ebi.ac.uk/QuickGO/GTerm?id=GO:0008285), [fibroblast growth factor receptor signaling pathway](http://www.ebi.ac.uk/QuickGO/GTerm?id=GO:0008543), [glial cell differentiation](http://www.ebi.ac.uk/QuickGO/GTerm?id=GO:0010001), [positive regulation of endothelial cell migration](http://www.ebi.ac.uk/QuickGO/GTerm?id=GO:0010595), [positive regulation of plasminogen activation](http://www.ebi.ac.uk/QuickGO/GTerm?id=GO:0010756), [growth factor dependent regulation of skeletal muscle satellite cell proliferation](http://www.ebi.ac.uk/QuickGO/GTerm?id=GO:0014843), [substantia nigra development](http://www.ebi.ac.uk/QuickGO/GTerm?id=GO:0021762), [positive regulation of cerebellar granule cell precursor proliferation](http://www.ebi.ac.uk/QuickGO/GTerm?id=GO:0021940), [neurogenesis](http://www.ebi.ac.uk/QuickGO/GTerm?id=GO:0022008), [cell differentiation](http://www.ebi.ac.uk/QuickGO/GTerm?id=GO:0030154), [lung development](http://www.ebi.ac.uk/QuickGO/GTerm?id=GO:0030324), [wound healing](http://www.ebi.ac.uk/QuickGO/GTerm?id=GO:0042060), [positive regulation of osteoblast differentiation](http://www.ebi.ac.uk/QuickGO/GTerm?id=GO:0045669), [positive regulation of angiogenesis](http://www.ebi.ac.uk/QuickGO/GTerm?id=GO:0045766), [positive regulation of transcription from RNA polymerase II promoter](http://www.ebi.ac.uk/QuickGO/GTerm?id=GO:0045944), [regulation of retinal cell programmed cell death](http://www.ebi.ac.uk/QuickGO/GTerm?id=GO:0046668), [response to axon injury](http://www.ebi.ac.uk/QuickGO/GTerm?id=GO:0048678), [stem cell development](http://www.ebi.ac.uk/QuickGO/GTerm?id=GO:0048864), [cell division](http://www.ebi.ac.uk/QuickGO/GTerm?id=GO:0051301), [regulation of cell cycle](http://www.ebi.ac.uk/QuickGO/GTerm?id=GO:0051726), [positive regulation of cell division](http://www.ebi.ac.uk/QuickGO/GTerm?id=GO:0051781), [positive regulation of protein kinase B signaling](http://www.ebi.ac.uk/QuickGO/GTerm?id=GO:0051897), [corticotropin hormone secreting cell differentiation](http://www.ebi.ac.uk/QuickGO/GTerm?id=GO:0060128), [thyroid-stimulating hormone-secreting cell differentiation](http://www.ebi.ac.uk/QuickGO/GTerm?id=GO:0060129), [neuroepithelial cell differentiation](http://www.ebi.ac.uk/QuickGO/GTerm?id=GO:0060563), [positive regulation of ERK1 and ERK2 cascade](http://www.ebi.ac.uk/QuickGO/GTerm?id=GO:0070374), [inner ear receptor cell differentiation involved in inner ear sensory epithelium regeneration](http://www.ebi.ac.uk/QuickGO/GTerm?id=GO:0070660), [stem cell proliferation](http://www.ebi.ac.uk/QuickGO/GTerm?id=GO:0072089), [metanephric glomerular visceral epithelial cell differentiation](http://www.ebi.ac.uk/QuickGO/GTerm?id=GO:0072248), [positive regulation of canonical Wnt signaling pathway](http://www.ebi.ac.uk/QuickGO/GTerm?id=GO:0090263), |
| **GOTERM_CC_DIRECT** | [extracellular region](http://www.ebi.ac.uk/QuickGO/GTerm?id=GO:0005576), [extracellular space](http://www.ebi.ac.uk/QuickGO/GTerm?id=GO:0005615), [nucleus](http://www.ebi.ac.uk/QuickGO/GTerm?id=GO:0005634), [nucleolus](http://www.ebi.ac.uk/QuickGO/GTerm?id=GO:0005730), [cytosol](http://www.ebi.ac.uk/QuickGO/GTerm?id=GO:0005829), |
| **GOTERM_MF_DIRECT** | [fibroblast growth factor receptor binding](http://www.ebi.ac.uk/QuickGO/GTerm?id=GO:0005104), [growth factor activity](http://www.ebi.ac.uk/QuickGO/GTerm?id=GO:0008083), [heparin binding](http://www.ebi.ac.uk/QuickGO/GTerm?id=GO:0008201), |
| **INTERPRO** | [Heparin-binding growth factor/Fibroblast growth factor](http://www.ebi.ac.uk/interpro/IEntry?ac=IPR002209), [Cytokine, IL-1-like](http://www.ebi.ac.uk/interpro/IEntry?ac=IPR008996), |
| **KEGG_PATHWAY** | [MAPK signaling pathway](https://david.ncifcrf.gov/kegg.jsp?path=gga04010$MAPK%20signaling%20pathway&termId=550025643&source=kegg), [Regulation of actin cytoskeleton](https://david.ncifcrf.gov/kegg.jsp?path=gga04810$Regulation%20of%20actin%20cytoskeleton&termId=550025686&source=kegg), |
| **SMART** | [FGF](http://smart.embl.de/smart/do_annotation.pl?DOMAIN=SM00442), |
| **UP_KEYWORDS** | [Angiogenesis](http://www.uniprot.org/keywords/?query=Angiogenesis), [Complete proteome](http://www.uniprot.org/keywords/?query=Complete%20proteome), [Developmental protein](http://www.uniprot.org/keywords/?query=Developmental%20protein), [Differentiation](http://www.uniprot.org/keywords/?query=Differentiation), [Growth factor](http://www.uniprot.org/keywords/?query=Growth%20factor), [Heparin-binding](http://www.uniprot.org/keywords/?query=Heparin-binding), [Mitogen](http://www.uniprot.org/keywords/?query=Mitogen), [Nucleus](http://www.uniprot.org/keywords/?query=Nucleus), [Reference proteome](http://www.uniprot.org/keywords/?query=Reference%20proteome), [Secreted](http://www.uniprot.org/keywords/?query=Secreted), |
| **UP_SEQ_FEATURE** | binding site:Heparin, chain:Heparin-binding growth factor 2, region of interest:Heparin-binding, |
| **FMO3** | [**flavin-containing monooxygenase 3(FMO3)**](https://david.ncifcrf.gov/geneReportFull.jsp?rowids=395267) |
| **GOTERM_BP_DIRECT** | [oxidation-reduction process](http://www.ebi.ac.uk/QuickGO/GTerm?id=GO:0055114), |
| **GOTERM_CC_DIRECT** | [endoplasmic reticulum membrane](http://www.ebi.ac.uk/QuickGO/GTerm?id=GO:0005789), [integral component of membrane](http://www.ebi.ac.uk/QuickGO/GTerm?id=GO:0016021), [organelle membrane](http://www.ebi.ac.uk/QuickGO/GTerm?id=GO:0031090), |
| **GOTERM_MF_DIRECT** | [monooxygenase activity](http://www.ebi.ac.uk/QuickGO/GTerm?id=GO:0004497), [N,N-dimethylaniline monooxygenase activity](http://www.ebi.ac.uk/QuickGO/GTerm?id=GO:0004499), [flavin adenine dinucleotide binding](http://www.ebi.ac.uk/QuickGO/GTerm?id=GO:0050660), [NADP binding](http://www.ebi.ac.uk/QuickGO/GTerm?id=GO:0050661), |
| **INTERPRO** | [Flavin monooxygenase FMO](http://www.ebi.ac.uk/interpro/IEntry?ac=IPR000960), [Flavin monooxygenase (FMO) 3](http://www.ebi.ac.uk/interpro/IEntry?ac=IPR002255), [Dimethylaniline monooxygenase, N-oxide-forming](http://www.ebi.ac.uk/interpro/IEntry?ac=IPR012143), [Flavin monooxygenase-like](http://www.ebi.ac.uk/interpro/IEntry?ac=IPR020946), [Pyridine nucleotide-disulphide oxidoreductase, FAD/NAD(P)-binding domain](http://www.ebi.ac.uk/interpro/IEntry?ac=IPR023753), |
| **KEGG_PATHWAY** | [Drug metabolism - cytochrome P450](https://david.ncifcrf.gov/kegg.jsp?path=gga00982$Drug%20metabolism%20-%20cytochrome%20P450&termId=550025614&source=kegg), |
| **PIR_SUPERFAMILY** | [dimethylaniline monooxygenase (N-oxide-forming)](http://pir.georgetown.edu/cgi-bin/ipcSF?id=PIRSF000332), |
| **UP_KEYWORDS** | [Complete proteome](http://www.uniprot.org/keywords/?query=Complete%20proteome), [Endoplasmic reticulum](http://www.uniprot.org/keywords/?query=Endoplasmic%20reticulum), [FAD](http://www.uniprot.org/keywords/?query=FAD), [Flavoprotein](http://www.uniprot.org/keywords/?query=Flavoprotein), [Membrane](http://www.uniprot.org/keywords/?query=Membrane), [Microsome](http://www.uniprot.org/keywords/?query=Microsome), [Monooxygenase](http://www.uniprot.org/keywords/?query=Monooxygenase), [NADP](http://www.uniprot.org/keywords/?query=NADP), [Oxidoreductase](http://www.uniprot.org/keywords/?query=Oxidoreductase), [Reference proteome](http://www.uniprot.org/keywords/?query=Reference%20proteome), [Transmembrane](http://www.uniprot.org/keywords/?query=Transmembrane), [Transmembrane helix](http://www.uniprot.org/keywords/?query=Transmembrane%20helix), |
| **GGNBP2** | [**gametogenetin binding protein 2(GGNBP2)**](https://david.ncifcrf.gov/geneReportFull.jsp?rowids=425053) |
| **GOTERM_BP_DIRECT** | [multicellular organism development](http://www.ebi.ac.uk/QuickGO/GTerm?id=GO:0007275), [spermatogenesis](http://www.ebi.ac.uk/QuickGO/GTerm?id=GO:0007283), [cell differentiation](http://www.ebi.ac.uk/QuickGO/GTerm?id=GO:0030154), |
| **GOTERM_CC_DIRECT** | [nucleus](http://www.ebi.ac.uk/QuickGO/GTerm?id=GO:0005634), [cytoplasm](http://www.ebi.ac.uk/QuickGO/GTerm?id=GO:0005737), |
| **INTERPRO** | [Gametogenetin-binding protein 2](http://www.ebi.ac.uk/interpro/IEntry?ac=IPR026073), |
| **UP_KEYWORDS** | [Alternative splicing](http://www.uniprot.org/keywords/?query=Alternative%20splicing), [Complete proteome](http://www.uniprot.org/keywords/?query=Complete%20proteome), [Cytoplasm](http://www.uniprot.org/keywords/?query=Cytoplasm), [Developmental protein](http://www.uniprot.org/keywords/?query=Developmental%20protein), [Differentiation](http://www.uniprot.org/keywords/?query=Differentiation), [Reference proteome](http://www.uniprot.org/keywords/?query=Reference%20proteome), [Spermatogenesis](http://www.uniprot.org/keywords/?query=Spermatogenesis), |
| **UP_SEQ_FEATURE** | chain:Gametogenetin-binding protein 2, sequence conflict, splice variant, |
| **GABRB2** | [**gamma-aminobutyric acid (GABA) A receptor, beta 2(GABRB2)**](https://david.ncifcrf.gov/geneReportFull.jsp?rowids=414890) |
| **GOTERM_BP_DIRECT** | [ion transport](http://www.ebi.ac.uk/QuickGO/GTerm?id=GO:0006811), [sensory perception of sound](http://www.ebi.ac.uk/QuickGO/GTerm?id=GO:0007605), [negative regulation of neuron apoptotic process](http://www.ebi.ac.uk/QuickGO/GTerm?id=GO:0043524), [inner ear receptor cell development](http://www.ebi.ac.uk/QuickGO/GTerm?id=GO:0060119), [innervation](http://www.ebi.ac.uk/QuickGO/GTerm?id=GO:0060384), [cochlea development](http://www.ebi.ac.uk/QuickGO/GTerm?id=GO:0090102), |
| **GOTERM_CC_DIRECT** | [plasma membrane](http://www.ebi.ac.uk/QuickGO/GTerm?id=GO:0005886), [integral component of membrane](http://www.ebi.ac.uk/QuickGO/GTerm?id=GO:0016021), [cell junction](http://www.ebi.ac.uk/QuickGO/GTerm?id=GO:0030054), [synapse](http://www.ebi.ac.uk/QuickGO/GTerm?id=GO:0045202), [extracellular exosome](http://www.ebi.ac.uk/QuickGO/GTerm?id=GO:0070062), [GABA-A receptor complex](http://www.ebi.ac.uk/QuickGO/GTerm?id=GO:1902711), |
| **GOTERM_MF_DIRECT** | [GABA-A receptor activity](http://www.ebi.ac.uk/QuickGO/GTerm?id=GO:0004890), [extracellular ligand-gated ion channel activity](http://www.ebi.ac.uk/QuickGO/GTerm?id=GO:0005230), |
| **INTERPRO** | [Gamma-aminobutyric-acid A receptor, beta subunit](http://www.ebi.ac.uk/interpro/IEntry?ac=IPR002289), [Gamma-aminobutyric acid A receptor](http://www.ebi.ac.uk/interpro/IEntry?ac=IPR006028), [Neurotransmitter-gated ion-channel transmembrane domain](http://www.ebi.ac.uk/interpro/IEntry?ac=IPR006029), [Neurotransmitter-gated ion-channel](http://www.ebi.ac.uk/interpro/IEntry?ac=IPR006201), [Neurotransmitter-gated ion-channel ligand-binding](http://www.ebi.ac.uk/interpro/IEntry?ac=IPR006202), [Neurotransmitter-gated ion-channel, conserved site](http://www.ebi.ac.uk/interpro/IEntry?ac=IPR018000), |
| **KEGG_PATHWAY** | [Neuroactive ligand-receptor interaction](https://david.ncifcrf.gov/kegg.jsp?path=gga04080$Neuroactive%20ligand-receptor%20interaction&termId=550025649&source=kegg), |
| **UP_KEYWORDS** | [Cell junction](http://www.uniprot.org/keywords/?query=Cell%20junction), [Cell membrane](http://www.uniprot.org/keywords/?query=Cell%20membrane), [Complete proteome](http://www.uniprot.org/keywords/?query=Complete%20proteome), [Disulfide bond](http://www.uniprot.org/keywords/?query=Disulfide%20bond), [Ion channel](http://www.uniprot.org/keywords/?query=Ion%20channel), [Ion transport](http://www.uniprot.org/keywords/?query=Ion%20transport), [Membrane](http://www.uniprot.org/keywords/?query=Membrane), [Receptor](http://www.uniprot.org/keywords/?query=Receptor), [Reference proteome](http://www.uniprot.org/keywords/?query=Reference%20proteome), [Signal](http://www.uniprot.org/keywords/?query=Signal), [Synapse](http://www.uniprot.org/keywords/?query=Synapse), [Transmembrane](http://www.uniprot.org/keywords/?query=Transmembrane), [Transmembrane helix](http://www.uniprot.org/keywords/?query=Transmembrane%20helix), [Transport](http://www.uniprot.org/keywords/?query=Transport), |
| **GLUL** | [**glutamate-ammonia ligase(GLUL)**](https://david.ncifcrf.gov/geneReportFull.jsp?rowids=396489) |
| **GOTERM_BP_DIRECT** | [glutamine biosynthetic process](http://www.ebi.ac.uk/QuickGO/GTerm?id=GO:0006542), |
| **GOTERM_CC_DIRECT** | [mitochondrion](http://www.ebi.ac.uk/QuickGO/GTerm?id=GO:0005739), |
| **GOTERM_MF_DIRECT** | [glutamate decarboxylase activity](http://www.ebi.ac.uk/QuickGO/GTerm?id=GO:0004351), [glutamate-ammonia ligase activity](http://www.ebi.ac.uk/QuickGO/GTerm?id=GO:0004356), [ATP binding](http://www.ebi.ac.uk/QuickGO/GTerm?id=GO:0005524), |
| **INTERPRO** | [Glutamine synthetase, catalytic domain](http://www.ebi.ac.uk/interpro/IEntry?ac=IPR008146), [Glutamine synthetase, beta-Grasp](http://www.ebi.ac.uk/interpro/IEntry?ac=IPR008147), [Glutamine synthetase/guanido kinase, catalytic domain](http://www.ebi.ac.uk/interpro/IEntry?ac=IPR014746), [Glutamine synthetase, N-terminal conserved site](http://www.ebi.ac.uk/interpro/IEntry?ac=IPR027302), [Glutamine synthetase, glycine-rich site](http://www.ebi.ac.uk/interpro/IEntry?ac=IPR027303), |
| **KEGG_PATHWAY** | [Arginine biosynthesis](https://david.ncifcrf.gov/kegg.jsp?path=gga00220$Arginine%20biosynthesis&termId=550025548&source=kegg), [Alanine, aspartate and glutamate metabolism](https://david.ncifcrf.gov/kegg.jsp?path=gga00250$Alanine,%20aspartate%20and%20glutamate%20metabolism&termId=550025552&source=kegg), [Glyoxylate and dicarboxylate metabolism](https://david.ncifcrf.gov/kegg.jsp?path=gga00630$Glyoxylate%20and%20dicarboxylate%20metabolism&termId=550025595&source=kegg), [Nitrogen metabolism](https://david.ncifcrf.gov/kegg.jsp?path=gga00910$Nitrogen%20metabolism&termId=550025610&source=kegg), [Metabolic pathways](https://david.ncifcrf.gov/kegg.jsp?path=gga01100$Metabolic%20pathways&termId=550025617&source=kegg), [Biosynthesis of amino acids](https://david.ncifcrf.gov/kegg.jsp?path=gga01230$Biosynthesis%20of%20amino%20acids&termId=550025623&source=kegg), |
| **SMART** | [SM01230](http://smart.embl.de/smart/do_annotation.pl?DOMAIN=SM01230), |
| **UP_KEYWORDS** | [ATP-binding](http://www.uniprot.org/keywords/?query=ATP-binding), [Complete proteome](http://www.uniprot.org/keywords/?query=Complete%20proteome), [Cytoplasm](http://www.uniprot.org/keywords/?query=Cytoplasm), [Ligase](http://www.uniprot.org/keywords/?query=Ligase), [Lyase](http://www.uniprot.org/keywords/?query=Lyase), [Mitochondrion](http://www.uniprot.org/keywords/?query=Mitochondrion), [Nucleotide-binding](http://www.uniprot.org/keywords/?query=Nucleotide-binding), [Reference proteome](http://www.uniprot.org/keywords/?query=Reference%20proteome), |
| **UP_SEQ_FEATURE** | chain:Glutamine synthetase, |
| **HSD17B2** | [**hydroxysteroid (17-beta) dehydrogenase 2(HSD17B2)**](https://david.ncifcrf.gov/geneReportFull.jsp?rowids=415807) |
| **GOTERM_BP_DIRECT** |  |
| **GOTERM_CC_DIRECT** | [integral component of membrane](http://www.ebi.ac.uk/QuickGO/GTerm?id=GO:0016021), |
| **GOTERM_MF_DIRECT** | [estradiol 17-beta-dehydrogenase activity](http://www.ebi.ac.uk/QuickGO/GTerm?id=GO:0004303), [testosterone dehydrogenase (NAD+) activity](http://www.ebi.ac.uk/QuickGO/GTerm?id=GO:0047035), |
| **INTERPRO** | [Glucose/ribitol dehydrogenase](http://www.ebi.ac.uk/interpro/IEntry?ac=IPR002347), [NAD(P)-binding domain](http://www.ebi.ac.uk/interpro/IEntry?ac=IPR016040), [Short-chain dehydrogenase/reductase, conserved site](http://www.ebi.ac.uk/interpro/IEntry?ac=IPR020904), |
| **KEGG_PATHWAY** | [Steroid hormone biosynthesis](https://david.ncifcrf.gov/kegg.jsp?path=gga00140$Steroid%20hormone%20biosynthesis&termId=550025546&source=kegg), [Metabolic pathways](https://david.ncifcrf.gov/kegg.jsp?path=gga01100$Metabolic%20pathways&termId=550025617&source=kegg), |
| **UP_KEYWORDS** | [Complete proteome](http://www.uniprot.org/keywords/?query=Complete%20proteome), [Membrane](http://www.uniprot.org/keywords/?query=Membrane), [Reference proteome](http://www.uniprot.org/keywords/?query=Reference%20proteome), [Transmembrane](http://www.uniprot.org/keywords/?query=Transmembrane), [Transmembrane helix](http://www.uniprot.org/keywords/?query=Transmembrane%20helix), |
| **IKBKE** | [**inhibitor of kappa light polypeptide gene enhancer in B-cells, kinase epsilon(IKBKE)**](https://david.ncifcrf.gov/geneReportFull.jsp?rowids=430480) |
| **GOTERM_BP_DIRECT** | [intrinsic apoptotic signaling pathway in response to DNA damage](http://www.ebi.ac.uk/QuickGO/GTerm?id=GO:0008630), [response to type I interferon](http://www.ebi.ac.uk/QuickGO/GTerm?id=GO:0034340), [response to interferon-beta](http://www.ebi.ac.uk/QuickGO/GTerm?id=GO:0035456), [positive regulation of I-kappaB kinase/NF-kappaB signaling](http://www.ebi.ac.uk/QuickGO/GTerm?id=GO:0043123), [protein homooligomerization](http://www.ebi.ac.uk/QuickGO/GTerm?id=GO:0051260), [cellular response to virus](http://www.ebi.ac.uk/QuickGO/GTerm?id=GO:0098586), |
| **GOTERM_CC_DIRECT** | [PML body](http://www.ebi.ac.uk/QuickGO/GTerm?id=GO:0016605), [mitochondrial membrane](http://www.ebi.ac.uk/QuickGO/GTerm?id=GO:0031966), |
| **GOTERM_MF_DIRECT** | [NF-kappaB-inducing kinase activity](http://www.ebi.ac.uk/QuickGO/GTerm?id=GO:0004704), [ATP binding](http://www.ebi.ac.uk/QuickGO/GTerm?id=GO:0005524), [IkappaB kinase activity](http://www.ebi.ac.uk/QuickGO/GTerm?id=GO:0008384), |
| **INTERPRO** | [Protein kinase, catalytic domain](http://www.ebi.ac.uk/interpro/IEntry?ac=IPR000719), [Protein kinase-like domain](http://www.ebi.ac.uk/interpro/IEntry?ac=IPR011009), [Protein kinase, ATP binding site](http://www.ebi.ac.uk/interpro/IEntry?ac=IPR017441), |
| **KEGG_PATHWAY** | [Toll-like receptor signaling pathway](https://david.ncifcrf.gov/kegg.jsp?path=gga04620$Toll-like%20receptor%20signaling%20pathway&termId=550025679&source=kegg), [RIG-I-like receptor signaling pathway](https://david.ncifcrf.gov/kegg.jsp?path=gga04622$RIG-I-like%20receptor%20signaling%20pathway&termId=550025681&source=kegg), [Cytosolic DNA-sensing pathway](https://david.ncifcrf.gov/kegg.jsp?path=gga04623$Cytosolic%20DNA-sensing%20pathway&termId=550025682&source=kegg), [Influenza A](https://david.ncifcrf.gov/kegg.jsp?path=gga05164$Influenza%20A&termId=550025694&source=kegg), [Herpes simplex infection](https://david.ncifcrf.gov/kegg.jsp?path=gga05168$Herpes%20simplex%20infection&termId=550025695&source=kegg), |
| **SMART** | [S_TKc](http://smart.embl.de/smart/do_annotation.pl?DOMAIN=SM00220), |
| **UP_KEYWORDS** | [Complete proteome](http://www.uniprot.org/keywords/?query=Complete%20proteome), [Reference proteome](http://www.uniprot.org/keywords/?query=Reference%20proteome), |
| **IL10** | [**interleukin 10(IL10)**](https://david.ncifcrf.gov/geneReportFull.jsp?rowids=428264) |
| **GOTERM_BP_DIRECT** | [negative regulation of cytokine secretion involved in immune response](http://www.ebi.ac.uk/QuickGO/GTerm?id=GO:0002740), [negative regulation of T-helper 1 type immune response](http://www.ebi.ac.uk/QuickGO/GTerm?id=GO:0002826), [negative regulation of chronic inflammatory response to antigenic stimulus](http://www.ebi.ac.uk/QuickGO/GTerm?id=GO:0002875), [positive regulation of B cell apoptotic process](http://www.ebi.ac.uk/QuickGO/GTerm?id=GO:0002904), [inflammatory response](http://www.ebi.ac.uk/QuickGO/GTerm?id=GO:0006954), [immune response](http://www.ebi.ac.uk/QuickGO/GTerm?id=GO:0006955), [negative regulation of myeloid dendritic cell activation](http://www.ebi.ac.uk/QuickGO/GTerm?id=GO:0030886), [negative regulation of B cell proliferation](http://www.ebi.ac.uk/QuickGO/GTerm?id=GO:0030889), [negative regulation of interferon-gamma production](http://www.ebi.ac.uk/QuickGO/GTerm?id=GO:0032689), [negative regulation of interleukin-12 production](http://www.ebi.ac.uk/QuickGO/GTerm?id=GO:0032695), [negative regulation of interleukin-6 production](http://www.ebi.ac.uk/QuickGO/GTerm?id=GO:0032715), [negative regulation of tumor necrosis factor production](http://www.ebi.ac.uk/QuickGO/GTerm?id=GO:0032720), [receptor biosynthetic process](http://www.ebi.ac.uk/QuickGO/GTerm?id=GO:0032800), [cellular response to hepatocyte growth factor stimulus](http://www.ebi.ac.uk/QuickGO/GTerm?id=GO:0035729), [defense response to bacterium](http://www.ebi.ac.uk/QuickGO/GTerm?id=GO:0042742), [defense response to protozoan](http://www.ebi.ac.uk/QuickGO/GTerm?id=GO:0042832), [negative regulation of growth of symbiont in host](http://www.ebi.ac.uk/QuickGO/GTerm?id=GO:0044130), [negative regulation of nitric oxide biosynthetic process](http://www.ebi.ac.uk/QuickGO/GTerm?id=GO:0045019), [negative regulation of interferon-gamma biosynthetic process](http://www.ebi.ac.uk/QuickGO/GTerm?id=GO:0045077), [positive regulation of MHC class II biosynthetic process](http://www.ebi.ac.uk/QuickGO/GTerm?id=GO:0045348), [positive regulation of transcription from RNA polymerase II promoter](http://www.ebi.ac.uk/QuickGO/GTerm?id=GO:0045944), [positive regulation of JAK-STAT cascade](http://www.ebi.ac.uk/QuickGO/GTerm?id=GO:0046427), [positive regulation of cytokine secretion](http://www.ebi.ac.uk/QuickGO/GTerm?id=GO:0050715), [negative regulation of membrane protein ectodomain proteolysis](http://www.ebi.ac.uk/QuickGO/GTerm?id=GO:0051045), [positive regulation of sequence-specific DNA binding transcription factor activity](http://www.ebi.ac.uk/QuickGO/GTerm?id=GO:0051091), [response to glucocorticoid](http://www.ebi.ac.uk/QuickGO/GTerm?id=GO:0051384), [negative regulation of cytokine activity](http://www.ebi.ac.uk/QuickGO/GTerm?id=GO:0060302), [cellular response to lipopolysaccharide](http://www.ebi.ac.uk/QuickGO/GTerm?id=GO:0071222), |
| **GOTERM_CC_DIRECT** | [extracellular region](http://www.ebi.ac.uk/QuickGO/GTerm?id=GO:0005576), [extracellular space](http://www.ebi.ac.uk/QuickGO/GTerm?id=GO:0005615), |
| **GOTERM_MF_DIRECT** | [cytokine activity](http://www.ebi.ac.uk/QuickGO/GTerm?id=GO:0005125), |
| **INTERPRO** | [Interleukin-10](http://www.ebi.ac.uk/interpro/IEntry?ac=IPR000098), [Four-helical cytokine-like, core](http://www.ebi.ac.uk/interpro/IEntry?ac=IPR009079), [Four-helical cytokine, core](http://www.ebi.ac.uk/interpro/IEntry?ac=IPR012351), [Interleukin-10/19/20/24](http://www.ebi.ac.uk/interpro/IEntry?ac=IPR020443), |
| **KEGG_PATHWAY** | [Cytokine-cytokine receptor interaction](https://david.ncifcrf.gov/kegg.jsp?path=gga04060$Cytokine-cytokine%20receptor%20interaction&termId=550025646&source=kegg), [FoxO signaling pathway](https://david.ncifcrf.gov/kegg.jsp?path=gga04068$FoxO%20signaling%20pathway&termId=550025647&source=kegg), [Jak-STAT signaling pathway](https://david.ncifcrf.gov/kegg.jsp?path=gga04630$Jak-STAT%20signaling%20pathway&termId=550025683&source=kegg), [Intestinal immune network for IgA production](https://david.ncifcrf.gov/kegg.jsp?path=gga04672$Intestinal%20immune%20network%20for%20IgA%20production&termId=550025684&source=kegg), |
| **SMART** | [IL10](http://smart.embl.de/smart/do_annotation.pl?DOMAIN=SM00188), |
| **UP_KEYWORDS** | [Complete proteome](http://www.uniprot.org/keywords/?query=Complete%20proteome), [Cytokine](http://www.uniprot.org/keywords/?query=Cytokine), [Disulfide bond](http://www.uniprot.org/keywords/?query=Disulfide%20bond), [Reference proteome](http://www.uniprot.org/keywords/?query=Reference%20proteome), [Secreted](http://www.uniprot.org/keywords/?query=Secreted), [Signal](http://www.uniprot.org/keywords/?query=Signal), |
| **UP_SEQ_FEATURE** | chain:Interleukin-10, disulfide bond, sequence conflict, signal peptide, |
| **IL19** | [**interleukin 19(IL19)**](https://david.ncifcrf.gov/geneReportFull.jsp?rowids=428265) |
| **KEGG_PATHWAY** | [Jak-STAT signaling pathway](https://david.ncifcrf.gov/kegg.jsp?path=gga04630$Jak-STAT%20signaling%20pathway&termId=550025683&source=kegg), |
| **IL8L2** | [**interleukin 8-like 2(IL8L2)**](https://david.ncifcrf.gov/geneReportFull.jsp?rowids=396495) |
| **GOTERM_BP_DIRECT** | [angiogenesis](http://www.ebi.ac.uk/QuickGO/GTerm?id=GO:0001525), [leukocyte chemotaxis involved in inflammatory response](http://www.ebi.ac.uk/QuickGO/GTerm?id=GO:0002232), [leukocyte activation involved in inflammatory response](http://www.ebi.ac.uk/QuickGO/GTerm?id=GO:0002269), [monocyte chemotaxis](http://www.ebi.ac.uk/QuickGO/GTerm?id=GO:0002548), [inflammatory response](http://www.ebi.ac.uk/QuickGO/GTerm?id=GO:0006954), [immune response](http://www.ebi.ac.uk/QuickGO/GTerm?id=GO:0006955), [G-protein coupled receptor signaling pathway](http://www.ebi.ac.uk/QuickGO/GTerm?id=GO:0007186), [response to bacterium](http://www.ebi.ac.uk/QuickGO/GTerm?id=GO:0009617), [positive regulation of cellular defense response](http://www.ebi.ac.uk/QuickGO/GTerm?id=GO:0010186), [positive regulation of gene expression](http://www.ebi.ac.uk/QuickGO/GTerm?id=GO:0010628), [hemopoiesis](http://www.ebi.ac.uk/QuickGO/GTerm?id=GO:0030097), [neutrophil chemotaxis](http://www.ebi.ac.uk/QuickGO/GTerm?id=GO:0030593), [leukocyte chemotaxis](http://www.ebi.ac.uk/QuickGO/GTerm?id=GO:0030595), [response to lipopolysaccharide](http://www.ebi.ac.uk/QuickGO/GTerm?id=GO:0032496), [myofibroblast differentiation](http://www.ebi.ac.uk/QuickGO/GTerm?id=GO:0036446), [wound healing](http://www.ebi.ac.uk/QuickGO/GTerm?id=GO:0042060), [neutrophil activation](http://www.ebi.ac.uk/QuickGO/GTerm?id=GO:0042119), [defense response to bacterium](http://www.ebi.ac.uk/QuickGO/GTerm?id=GO:0042742), [negative regulation of fibroblast proliferation](http://www.ebi.ac.uk/QuickGO/GTerm?id=GO:0048147), [macrophage chemotaxis](http://www.ebi.ac.uk/QuickGO/GTerm?id=GO:0048246), [lymphocyte chemotaxis](http://www.ebi.ac.uk/QuickGO/GTerm?id=GO:0048247), [positive regulation of inflammatory response](http://www.ebi.ac.uk/QuickGO/GTerm?id=GO:0050729), [chemokine-mediated signaling pathway](http://www.ebi.ac.uk/QuickGO/GTerm?id=GO:0070098), [positive regulation of neutrophil chemotaxis](http://www.ebi.ac.uk/QuickGO/GTerm?id=GO:0090023), |
| **GOTERM_CC_DIRECT** | [extracellular space](http://www.ebi.ac.uk/QuickGO/GTerm?id=GO:0005615), |
| **GOTERM_MF_DIRECT** | [cytokine activity](http://www.ebi.ac.uk/QuickGO/GTerm?id=GO:0005125), [chemokine activity](http://www.ebi.ac.uk/QuickGO/GTerm?id=GO:0008009), [growth factor activity](http://www.ebi.ac.uk/QuickGO/GTerm?id=GO:0008083), [CXCR chemokine receptor binding](http://www.ebi.ac.uk/QuickGO/GTerm?id=GO:0045236), |
| **INTERPRO** | [CXC chemokine](http://www.ebi.ac.uk/interpro/IEntry?ac=IPR001089), [Chemokine interleukin-8-like domain](http://www.ebi.ac.uk/interpro/IEntry?ac=IPR001811), [CXC chemokine, conserved site](http://www.ebi.ac.uk/interpro/IEntry?ac=IPR018048), |
| **KEGG_PATHWAY** | [Cytokine-cytokine receptor interaction](https://david.ncifcrf.gov/kegg.jsp?path=gga04060$Cytokine-cytokine%20receptor%20interaction&termId=550025646&source=kegg), [Toll-like receptor signaling pathway](https://david.ncifcrf.gov/kegg.jsp?path=gga04620$Toll-like%20receptor%20signaling%20pathway&termId=550025679&source=kegg), [NOD-like receptor signaling pathway](https://david.ncifcrf.gov/kegg.jsp?path=gga04621$NOD-like%20receptor%20signaling%20pathway&termId=550025680&source=kegg), [RIG-I-like receptor signaling pathway](https://david.ncifcrf.gov/kegg.jsp?path=gga04622$RIG-I-like%20receptor%20signaling%20pathway&termId=550025681&source=kegg), [Salmonella infection](https://david.ncifcrf.gov/kegg.jsp?path=gga05132$Salmonella%20infection&termId=550025693&source=kegg), [Influenza A](https://david.ncifcrf.gov/kegg.jsp?path=gga05164$Influenza%20A&termId=550025694&source=kegg), |
| **SMART** | [SCY](http://smart.embl.de/smart/do_annotation.pl?DOMAIN=SM00199), |
| **UP_KEYWORDS** | [Chemotaxis](http://www.uniprot.org/keywords/?query=Chemotaxis), [Complete proteome](http://www.uniprot.org/keywords/?query=Complete%20proteome), [Cytokine](http://www.uniprot.org/keywords/?query=Cytokine), [Disulfide bond](http://www.uniprot.org/keywords/?query=Disulfide%20bond), [Growth factor](http://www.uniprot.org/keywords/?query=Growth%20factor), [Reference proteome](http://www.uniprot.org/keywords/?query=Reference%20proteome), [Secreted](http://www.uniprot.org/keywords/?query=Secreted), [Signal](http://www.uniprot.org/keywords/?query=Signal), |
| **UP_SEQ_FEATURE** | chain:Interleukin-8, disulfide bond, sequence conflict, signal peptide, |
| **IFT22** | [**intraflagellar transport 22(IFT22)**](https://david.ncifcrf.gov/geneReportFull.jsp?rowids=417503) |
| **GOTERM_BP_DIRECT** | [small GTPase mediated signal transduction](http://www.ebi.ac.uk/QuickGO/GTerm?id=GO:0007264), |
| **GOTERM_CC_DIRECT** | [centrosome](http://www.ebi.ac.uk/QuickGO/GTerm?id=GO:0005813), [cilium](http://www.ebi.ac.uk/QuickGO/GTerm?id=GO:0005929), [intraciliary transport particle B](http://www.ebi.ac.uk/QuickGO/GTerm?id=GO:0030992), |
| **GOTERM_MF_DIRECT** | [GTP binding](http://www.ebi.ac.uk/QuickGO/GTerm?id=GO:0005525), |
| **INTERPRO** | [Mitochondrial Rho-like](http://www.ebi.ac.uk/interpro/IEntry?ac=IPR013684), [P-loop containing nucleoside triphosphate hydrolase](http://www.ebi.ac.uk/interpro/IEntry?ac=IPR027417), |
| **UP_KEYWORDS** | [Complete proteome](http://www.uniprot.org/keywords/?query=Complete%20proteome), [Reference proteome](http://www.uniprot.org/keywords/?query=Reference%20proteome), |
| **JARID2** | [**jumonji and AT-rich interaction domain containing 2(JARID2)**](https://david.ncifcrf.gov/geneReportFull.jsp?rowids=420839) |
| **GOTERM_BP_DIRECT** | [liver development](http://www.ebi.ac.uk/QuickGO/GTerm?id=GO:0001889), [transcription, DNA-templated](http://www.ebi.ac.uk/QuickGO/GTerm?id=GO:0006351), [multicellular organism development](http://www.ebi.ac.uk/QuickGO/GTerm?id=GO:0007275), [negative regulation of cell proliferation](http://www.ebi.ac.uk/QuickGO/GTerm?id=GO:0008285), [covalent chromatin modification](http://www.ebi.ac.uk/QuickGO/GTerm?id=GO:0016569), [negative regulation of histone methylation](http://www.ebi.ac.uk/QuickGO/GTerm?id=GO:0031061), [negative regulation of transcription, DNA-templated](http://www.ebi.ac.uk/QuickGO/GTerm?id=GO:0045892), [spleen development](http://www.ebi.ac.uk/QuickGO/GTerm?id=GO:0048536), [thymus development](http://www.ebi.ac.uk/QuickGO/GTerm?id=GO:0048538), [stem cell differentiation](http://www.ebi.ac.uk/QuickGO/GTerm?id=GO:0048863), [positive regulation of histone H3-K9 methylation](http://www.ebi.ac.uk/QuickGO/GTerm?id=GO:0051574), |
| **GOTERM_CC_DIRECT** | [nucleus](http://www.ebi.ac.uk/QuickGO/GTerm?id=GO:0005634), [histone methyltransferase complex](http://www.ebi.ac.uk/QuickGO/GTerm?id=GO:0035097), [ESC/E(Z) complex](http://www.ebi.ac.uk/QuickGO/GTerm?id=GO:0035098), |
| **GOTERM_MF_DIRECT** | [RNA polymerase II regulatory region sequence-specific DNA binding](http://www.ebi.ac.uk/QuickGO/GTerm?id=GO:0000977), [transcriptional repressor activity, RNA polymerase II transcription regulatory region sequence-specific binding](http://www.ebi.ac.uk/QuickGO/GTerm?id=GO:0001227), [DNA binding](http://www.ebi.ac.uk/QuickGO/GTerm?id=GO:0003677), [chromatin binding](http://www.ebi.ac.uk/QuickGO/GTerm?id=GO:0003682), [histone demethylase activity](http://www.ebi.ac.uk/QuickGO/GTerm?id=GO:0032452), |
| **INTERPRO** | [ARID/BRIGHT DNA-binding domain](http://www.ebi.ac.uk/interpro/IEntry?ac=IPR001606), [JmjC domain](http://www.ebi.ac.uk/interpro/IEntry?ac=IPR003347), [Transcription factor jumonji, JmjN](http://www.ebi.ac.uk/interpro/IEntry?ac=IPR003349), [Zinc finger, C5HC2-type](http://www.ebi.ac.uk/interpro/IEntry?ac=IPR004198), |
| **SMART** | [BRIGHT](http://smart.embl.de/smart/do_annotation.pl?DOMAIN=SM00501), [JmjN](http://smart.embl.de/smart/do_annotation.pl?DOMAIN=SM00545), [JmjC](http://smart.embl.de/smart/do_annotation.pl?DOMAIN=SM00558), |
| **UP_KEYWORDS** | [Chromatin regulator](http://www.uniprot.org/keywords/?query=Chromatin%20regulator), [Complete proteome](http://www.uniprot.org/keywords/?query=Complete%20proteome), [Developmental protein](http://www.uniprot.org/keywords/?query=Developmental%20protein), [Differentiation](http://www.uniprot.org/keywords/?query=Differentiation), [Nucleus](http://www.uniprot.org/keywords/?query=Nucleus), [Reference proteome](http://www.uniprot.org/keywords/?query=Reference%20proteome), [Repressor](http://www.uniprot.org/keywords/?query=Repressor), [Transcription](http://www.uniprot.org/keywords/?query=Transcription), [Transcription regulation](http://www.uniprot.org/keywords/?query=Transcription%20regulation), |
| **LECT2** | [**leukocyte cell derived chemotaxin 2(LECT2)**](https://david.ncifcrf.gov/geneReportFull.jsp?rowids=396471) |
| **GOTERM_BP_DIRECT** | [granulocyte differentiation](http://www.ebi.ac.uk/QuickGO/GTerm?id=GO:0030851), [response to antibiotic](http://www.ebi.ac.uk/QuickGO/GTerm?id=GO:0046677), |
| **GOTERM_CC_DIRECT** | [extracellular space](http://www.ebi.ac.uk/QuickGO/GTerm?id=GO:0005615), [cytolytic granule](http://www.ebi.ac.uk/QuickGO/GTerm?id=GO:0044194), |
| **INTERPRO** | [Leukocyte cell-derived chemotaxin 2](http://www.ebi.ac.uk/interpro/IEntry?ac=IPR008663), [Peptidase M23](http://www.ebi.ac.uk/interpro/IEntry?ac=IPR016047), |
| **UP_KEYWORDS** | [Complete proteome](http://www.uniprot.org/keywords/?query=Complete%20proteome), [Direct protein sequencing](http://www.uniprot.org/keywords/?query=Direct%20protein%20sequencing), [Reference proteome](http://www.uniprot.org/keywords/?query=Reference%20proteome), [Repeat](http://www.uniprot.org/keywords/?query=Repeat), [Signal](http://www.uniprot.org/keywords/?query=Signal), |
| **UP_SEQ_FEATURE** | chain:Myeloid protein 1, repeat:1, repeat:2, sequence conflict, signal peptide, |
| **MTA1** | [**metastasis associated 1(MTA1)**](https://david.ncifcrf.gov/geneReportFull.jsp?rowids=429060) |
| **GOTERM_BP_DIRECT** | [double-strand break repair](http://www.ebi.ac.uk/QuickGO/GTerm?id=GO:0006302), [response to ionizing radiation](http://www.ebi.ac.uk/QuickGO/GTerm?id=GO:0010212), [regulation of gene expression, epigenetic](http://www.ebi.ac.uk/QuickGO/GTerm?id=GO:0040029), [proteasome-mediated ubiquitin-dependent protein catabolic process](http://www.ebi.ac.uk/QuickGO/GTerm?id=GO:0043161), [positive regulation of protein autoubiquitination](http://www.ebi.ac.uk/QuickGO/GTerm?id=GO:1902499), |
| **GOTERM_CC_DIRECT** | [nucleus](http://www.ebi.ac.uk/QuickGO/GTerm?id=GO:0005634), [nuclear envelope](http://www.ebi.ac.uk/QuickGO/GTerm?id=GO:0005635), [nucleoplasm](http://www.ebi.ac.uk/QuickGO/GTerm?id=GO:0005654), [cytoplasm](http://www.ebi.ac.uk/QuickGO/GTerm?id=GO:0005737), [microtubule](http://www.ebi.ac.uk/QuickGO/GTerm?id=GO:0005874), |
| **GOTERM_MF_DIRECT** | [core promoter binding](http://www.ebi.ac.uk/QuickGO/GTerm?id=GO:0001047), [chromatin binding](http://www.ebi.ac.uk/QuickGO/GTerm?id=GO:0003682), [transcription factor activity, sequence-specific DNA binding](http://www.ebi.ac.uk/QuickGO/GTerm?id=GO:0003700), [transcription coactivator activity](http://www.ebi.ac.uk/QuickGO/GTerm?id=GO:0003713), [transcription corepressor activity](http://www.ebi.ac.uk/QuickGO/GTerm?id=GO:0003714), [zinc ion binding](http://www.ebi.ac.uk/QuickGO/GTerm?id=GO:0008270), [sequence-specific DNA binding](http://www.ebi.ac.uk/QuickGO/GTerm?id=GO:0043565), |
| **INTERPRO** | [Zinc finger, GATA-type](http://www.ebi.ac.uk/interpro/IEntry?ac=IPR000679), [ELM2 domain](http://www.ebi.ac.uk/interpro/IEntry?ac=IPR000949), [SANT/Myb domain](http://www.ebi.ac.uk/interpro/IEntry?ac=IPR001005), [Bromo adjacent homology (BAH) domain](http://www.ebi.ac.uk/interpro/IEntry?ac=IPR001025), [Homeodomain-like](http://www.ebi.ac.uk/interpro/IEntry?ac=IPR009057), [SANT domain](http://www.ebi.ac.uk/interpro/IEntry?ac=IPR017884), |
| **SMART** | [ZnF_GATA](http://smart.embl.de/smart/do_annotation.pl?DOMAIN=SM00401), [BAH](http://smart.embl.de/smart/do_annotation.pl?DOMAIN=SM00439), [SANT](http://smart.embl.de/smart/do_annotation.pl?DOMAIN=SM00717), [SM01189](http://smart.embl.de/smart/do_annotation.pl?DOMAIN=SM01189), |
| **UP_KEYWORDS** | [Complete proteome](http://www.uniprot.org/keywords/?query=Complete%20proteome), [Nucleus](http://www.uniprot.org/keywords/?query=Nucleus), [Reference proteome](http://www.uniprot.org/keywords/?query=Reference%20proteome), |
| **MRM1** | [**mitochondrial rRNA methyltransferase 1(MRM1)**](https://david.ncifcrf.gov/geneReportFull.jsp?rowids=772219) |
| **COG_ONTOLOGY** | [Translation, ribosomal structure and biogenesis](http://www.ncbi.nlm.nih.gov/COG/new/), |
| **GOTERM_BP_DIRECT** | [enzyme-directed rRNA 2'-O-methylation](http://www.ebi.ac.uk/QuickGO/GTerm?id=GO:0000453), |
| **GOTERM_CC_DIRECT** | [mitochondrion](http://www.ebi.ac.uk/QuickGO/GTerm?id=GO:0005739), |
| **GOTERM_MF_DIRECT** | [RNA binding](http://www.ebi.ac.uk/QuickGO/GTerm?id=GO:0003723), [poly(A) RNA binding](http://www.ebi.ac.uk/QuickGO/GTerm?id=GO:0044822), [rRNA (guanosine-2'-O-)-methyltransferase activity](http://www.ebi.ac.uk/QuickGO/GTerm?id=GO:0070039), |
| **INTERPRO** | [tRNA/rRNA methyltransferase, SpoU](http://www.ebi.ac.uk/interpro/IEntry?ac=IPR001537), [RNA 2-O ribose methyltransferase, substrate binding](http://www.ebi.ac.uk/interpro/IEntry?ac=IPR013123), |
| **SMART** | [SM00967](http://smart.embl.de/smart/do_annotation.pl?DOMAIN=SM00967), |
| **UP_KEYWORDS** | [Complete proteome](http://www.uniprot.org/keywords/?query=Complete%20proteome), [Reference proteome](http://www.uniprot.org/keywords/?query=Reference%20proteome), |
| **MAPKAPK2** | [**mitogen-activated protein kinase-activated protein kinase 2(MAPKAPK2)**](https://david.ncifcrf.gov/geneReportFull.jsp?rowids=419847) |
| **KEGG_PATHWAY** | [MAPK signaling pathway](https://david.ncifcrf.gov/kegg.jsp?path=gga04010$MAPK%20signaling%20pathway&termId=550025643&source=kegg), [VEGF signaling pathway](https://david.ncifcrf.gov/kegg.jsp?path=gga04370$VEGF%20signaling%20pathway&termId=550025672&source=kegg), |
| **MYO19** | [**myosin XIX(MYO19)**](https://david.ncifcrf.gov/geneReportFull.jsp?rowids=417649) |
| **GOTERM_CC_DIRECT** | [cytoplasm](http://www.ebi.ac.uk/QuickGO/GTerm?id=GO:0005737), [myosin complex](http://www.ebi.ac.uk/QuickGO/GTerm?id=GO:0016459), |
| **GOTERM_MF_DIRECT** | [ATP binding](http://www.ebi.ac.uk/QuickGO/GTerm?id=GO:0005524), [ATPase activity](http://www.ebi.ac.uk/QuickGO/GTerm?id=GO:0016887), [plus-end directed microfilament motor activity](http://www.ebi.ac.uk/QuickGO/GTerm?id=GO:0060002), |
| **INTERPRO** | [IQ motif, EF-hand binding site](http://www.ebi.ac.uk/interpro/IEntry?ac=IPR000048), [Myosin head, motor domain](http://www.ebi.ac.uk/interpro/IEntry?ac=IPR001609), [P-loop containing nucleoside triphosphate hydrolase](http://www.ebi.ac.uk/interpro/IEntry?ac=IPR027417), |
| **SMART** | [IQ](http://smart.embl.de/smart/do_annotation.pl?DOMAIN=SM00015), [MYSc](http://smart.embl.de/smart/do_annotation.pl?DOMAIN=SM00242), |
| **UP_KEYWORDS** | [Actin-binding](http://www.uniprot.org/keywords/?query=Actin-binding), [ATP-binding](http://www.uniprot.org/keywords/?query=ATP-binding), [Complete proteome](http://www.uniprot.org/keywords/?query=Complete%20proteome), [Motor protein](http://www.uniprot.org/keywords/?query=Motor%20protein), [Myosin](http://www.uniprot.org/keywords/?query=Myosin), [Nucleotide-binding](http://www.uniprot.org/keywords/?query=Nucleotide-binding), [Reference proteome](http://www.uniprot.org/keywords/?query=Reference%20proteome), |
| **MX1** | [**myxovirus (influenza virus) resistance 1, interferon-inducible protein p78 (mouse)(MX1)**](https://david.ncifcrf.gov/geneReportFull.jsp?rowids=395313) |
| **GOTERM_BP_DIRECT** | [innate immune response](http://www.ebi.ac.uk/QuickGO/GTerm?id=GO:0045087), [organelle fission](http://www.ebi.ac.uk/QuickGO/GTerm?id=GO:0048285), [defense response to virus](http://www.ebi.ac.uk/QuickGO/GTerm?id=GO:0051607), |
| **GOTERM_CC_DIRECT** | [nucleus](http://www.ebi.ac.uk/QuickGO/GTerm?id=GO:0005634), [cytoplasm](http://www.ebi.ac.uk/QuickGO/GTerm?id=GO:0005737), |
| **GOTERM_MF_DIRECT** | [GTPase activity](http://www.ebi.ac.uk/QuickGO/GTerm?id=GO:0003924), [GTP binding](http://www.ebi.ac.uk/QuickGO/GTerm?id=GO:0005525), [microtubule binding](http://www.ebi.ac.uk/QuickGO/GTerm?id=GO:0008017), |
| **INTERPRO** | [Dynamin central domain](http://www.ebi.ac.uk/interpro/IEntry?ac=IPR000375), [Dynamin, GTPase domain](http://www.ebi.ac.uk/interpro/IEntry?ac=IPR001401), [Dynamin GTPase effector](http://www.ebi.ac.uk/interpro/IEntry?ac=IPR003130), [Dynamin, GTPase region, conserved site](http://www.ebi.ac.uk/interpro/IEntry?ac=IPR019762), [GTPase effector domain, GED](http://www.ebi.ac.uk/interpro/IEntry?ac=IPR020850), [Dynamin](http://www.ebi.ac.uk/interpro/IEntry?ac=IPR022812), [P-loop containing nucleoside triphosphate hydrolase](http://www.ebi.ac.uk/interpro/IEntry?ac=IPR027417), |
| **KEGG_PATHWAY** | [Influenza A](https://david.ncifcrf.gov/kegg.jsp?path=gga05164$Influenza%20A&termId=550025694&source=kegg), |
| **SMART** | [DYNc](http://smart.embl.de/smart/do_annotation.pl?DOMAIN=SM00053), [GED](http://smart.embl.de/smart/do_annotation.pl?DOMAIN=SM00302), |
| **UP_KEYWORDS** | [Complete proteome](http://www.uniprot.org/keywords/?query=Complete%20proteome), [Cytoplasm](http://www.uniprot.org/keywords/?query=Cytoplasm), [GTP-binding](http://www.uniprot.org/keywords/?query=GTP-binding), [Nucleotide-binding](http://www.uniprot.org/keywords/?query=Nucleotide-binding), [Reference proteome](http://www.uniprot.org/keywords/?query=Reference%20proteome), |
| **UP_SEQ_FEATURE** | chain:Interferon-induced GTP-binding protein Mx, domain:GED, nucleotide phosphate-binding region:GTP, sequence variant, |
| **NUP88** | [**nucleoporin 88kDa(NUP88)**](https://david.ncifcrf.gov/geneReportFull.jsp?rowids=417501) |
| **GOTERM_BP_DIRECT** | [ribosomal large subunit export from nucleus](http://www.ebi.ac.uk/QuickGO/GTerm?id=GO:0000055), [ribosomal small subunit export from nucleus](http://www.ebi.ac.uk/QuickGO/GTerm?id=GO:0000056), [mRNA export from nucleus](http://www.ebi.ac.uk/QuickGO/GTerm?id=GO:0006406), [protein import into nucleus](http://www.ebi.ac.uk/QuickGO/GTerm?id=GO:0006606), [protein export from nucleus](http://www.ebi.ac.uk/QuickGO/GTerm?id=GO:0006611), |
| **GOTERM_CC_DIRECT** | [nuclear pore](http://www.ebi.ac.uk/QuickGO/GTerm?id=GO:0005643), [nucleoplasm](http://www.ebi.ac.uk/QuickGO/GTerm?id=GO:0005654), |
| **GOTERM_MF_DIRECT** | [transporter activity](http://www.ebi.ac.uk/QuickGO/GTerm?id=GO:0005215), |
| **INTERPRO** | [Nucleoporin Nup88](http://www.ebi.ac.uk/interpro/IEntry?ac=IPR019321), |
| **KEGG_PATHWAY** | [RNA transport](https://david.ncifcrf.gov/kegg.jsp?path=gga03013$RNA%20transport&termId=550025627&source=kegg), |
| **UP_KEYWORDS** | [Coiled coil](http://www.uniprot.org/keywords/?query=Coiled%20coil), [Complete proteome](http://www.uniprot.org/keywords/?query=Complete%20proteome), [Reference proteome](http://www.uniprot.org/keywords/?query=Reference%20proteome), |
| **PITX1** | [**paired-like homeodomain 1(PITX1)**](https://david.ncifcrf.gov/geneReportFull.jsp?rowids=374201) |
| **GOTERM_BP_DIRECT** | [transcription, DNA-templated](http://www.ebi.ac.uk/QuickGO/GTerm?id=GO:0006351), [regulation of transcription, DNA-templated](http://www.ebi.ac.uk/QuickGO/GTerm?id=GO:0006355), [multicellular organism development](http://www.ebi.ac.uk/QuickGO/GTerm?id=GO:0007275), [branchiomeric skeletal muscle development](http://www.ebi.ac.uk/QuickGO/GTerm?id=GO:0014707), [pituitary gland development](http://www.ebi.ac.uk/QuickGO/GTerm?id=GO:0021983), [embryonic hindlimb morphogenesis](http://www.ebi.ac.uk/QuickGO/GTerm?id=GO:0035116), [myoblast fate commitment](http://www.ebi.ac.uk/QuickGO/GTerm?id=GO:0048625), [cartilage development](http://www.ebi.ac.uk/QuickGO/GTerm?id=GO:0051216), |
| **GOTERM_CC_DIRECT** | [nucleus](http://www.ebi.ac.uk/QuickGO/GTerm?id=GO:0005634), [transcription factor complex](http://www.ebi.ac.uk/QuickGO/GTerm?id=GO:0005667), [nucleolus](http://www.ebi.ac.uk/QuickGO/GTerm?id=GO:0005730), [cytoplasm](http://www.ebi.ac.uk/QuickGO/GTerm?id=GO:0005737), |
| **GOTERM_MF_DIRECT** | [RNA polymerase II core promoter proximal region sequence-specific DNA binding](http://www.ebi.ac.uk/QuickGO/GTerm?id=GO:0000978), [transcriptional activator activity, RNA polymerase II core promoter proximal region sequence-specific binding](http://www.ebi.ac.uk/QuickGO/GTerm?id=GO:0001077), [transcriptional activator activity, RNA polymerase II transcription factor binding](http://www.ebi.ac.uk/QuickGO/GTerm?id=GO:0001190), [transcription factor activity, sequence-specific DNA binding](http://www.ebi.ac.uk/QuickGO/GTerm?id=GO:0003700), [sequence-specific DNA binding](http://www.ebi.ac.uk/QuickGO/GTerm?id=GO:0043565), |
| **INTERPRO** | [Homeodomain](http://www.ebi.ac.uk/interpro/IEntry?ac=IPR001356), [OAR domain](http://www.ebi.ac.uk/interpro/IEntry?ac=IPR003654), [Homeodomain-like](http://www.ebi.ac.uk/interpro/IEntry?ac=IPR009057), [Homeobox protein Pitx/unc30](http://www.ebi.ac.uk/interpro/IEntry?ac=IPR016233), [Homeobox, conserved site](http://www.ebi.ac.uk/interpro/IEntry?ac=IPR017970), |
| **PIR_SUPERFAMILY** | [homeobox protein, Pitx/Unc30 types](http://pir.georgetown.edu/cgi-bin/ipcSF?id=PIRSF000563), |
| **SMART** | [HOX](http://smart.embl.de/smart/do_annotation.pl?DOMAIN=SM00389), |
| **UP_KEYWORDS** | [Activator](http://www.uniprot.org/keywords/?query=Activator), [Complete proteome](http://www.uniprot.org/keywords/?query=Complete%20proteome), [Developmental protein](http://www.uniprot.org/keywords/?query=Developmental%20protein), [DNA-binding](http://www.uniprot.org/keywords/?query=DNA-binding), [Homeobox](http://www.uniprot.org/keywords/?query=Homeobox), [Nucleus](http://www.uniprot.org/keywords/?query=Nucleus), [Reference proteome](http://www.uniprot.org/keywords/?query=Reference%20proteome), [Transcription](http://www.uniprot.org/keywords/?query=Transcription), [Transcription regulation](http://www.uniprot.org/keywords/?query=Transcription%20regulation), |
| **UP_SEQ_FEATURE** | chain:Pituitary homeobox 1, compositionally biased region:Poly-Lys, DNA-binding region:Homeobox, region of interest:Interacts with PIT-1, sequence conflict, short sequence motif:Nuclear localization signal, short sequence motif:OAR, |
| **PEX2** | [**peroxisomal biogenesis factor 2(PEX2)**](https://david.ncifcrf.gov/geneReportFull.jsp?rowids=420192) |
| **GOTERM_BP_DIRECT** | [very long-chain fatty acid metabolic process](http://www.ebi.ac.uk/QuickGO/GTerm?id=GO:0000038), [negative regulation of transcription from RNA polymerase II promoter](http://www.ebi.ac.uk/QuickGO/GTerm?id=GO:0000122), [fatty acid beta-oxidation](http://www.ebi.ac.uk/QuickGO/GTerm?id=GO:0006635), [protein import into peroxisome matrix](http://www.ebi.ac.uk/QuickGO/GTerm?id=GO:0016558), [protein destabilization](http://www.ebi.ac.uk/QuickGO/GTerm?id=GO:0031648), [negative regulation of fibroblast proliferation](http://www.ebi.ac.uk/QuickGO/GTerm?id=GO:0048147), [negative regulation of epithelial cell proliferation](http://www.ebi.ac.uk/QuickGO/GTerm?id=GO:0050680), |
| **GOTERM_CC_DIRECT** | [integral component of peroxisomal membrane](http://www.ebi.ac.uk/QuickGO/GTerm?id=GO:0005779), [Cdc73/Paf1 complex](http://www.ebi.ac.uk/QuickGO/GTerm?id=GO:0016593), |
| **GOTERM_MF_DIRECT** | [zinc ion binding](http://www.ebi.ac.uk/QuickGO/GTerm?id=GO:0008270), |
| **INTERPRO** | [Zinc finger, RING-type](http://www.ebi.ac.uk/interpro/IEntry?ac=IPR001841), [Pex, N-terminal](http://www.ebi.ac.uk/interpro/IEntry?ac=IPR006845), [Zinc finger, RING/FYVE/PHD-type](http://www.ebi.ac.uk/interpro/IEntry?ac=IPR013083), [Zinc finger, RING-type, conserved site](http://www.ebi.ac.uk/interpro/IEntry?ac=IPR017907), [Zinc finger, C3HC4 RING-type](http://www.ebi.ac.uk/interpro/IEntry?ac=IPR018957), |
| **KEGG_PATHWAY** | [Peroxisome](https://david.ncifcrf.gov/kegg.jsp?path=gga04146$Peroxisome&termId=550025661&source=kegg), |
| **SMART** | [RING](http://smart.embl.de/smart/do_annotation.pl?DOMAIN=SM00184), |
| **UP_KEYWORDS** | [Complete proteome](http://www.uniprot.org/keywords/?query=Complete%20proteome), [Metal-binding](http://www.uniprot.org/keywords/?query=Metal-binding), [Reference proteome](http://www.uniprot.org/keywords/?query=Reference%20proteome), [Zinc](http://www.uniprot.org/keywords/?query=Zinc), [Zinc-finger](http://www.uniprot.org/keywords/?query=Zinc-finger), |
| **PIGW** | [**phosphatidylinositol glycan anchor biosynthesis class W(PIGW)**](https://david.ncifcrf.gov/geneReportFull.jsp?rowids=417650) |
| **GOTERM_BP_DIRECT** | [GPI anchor biosynthetic process](http://www.ebi.ac.uk/QuickGO/GTerm?id=GO:0006506), [protein localization to plasma membrane](http://www.ebi.ac.uk/QuickGO/GTerm?id=GO:0072659), |
| **GOTERM_CC_DIRECT** | [endoplasmic reticulum membrane](http://www.ebi.ac.uk/QuickGO/GTerm?id=GO:0005789), [integral component of membrane](http://www.ebi.ac.uk/QuickGO/GTerm?id=GO:0016021), |
| **GOTERM_MF_DIRECT** | [transferase activity, transferring acyl groups](http://www.ebi.ac.uk/QuickGO/GTerm?id=GO:0016746), |
| **INTERPRO** | [GWT1](http://www.ebi.ac.uk/interpro/IEntry?ac=IPR009447), |
| **KEGG_PATHWAY** | [Glycosylphosphatidylinositol(GPI)-anchor biosynthesis](https://david.ncifcrf.gov/kegg.jsp?path=gga00563$Glycosylphosphatidylinositol(GPI)-anchor%20biosynthesis&termId=550025584&source=kegg), [Metabolic pathways](https://david.ncifcrf.gov/kegg.jsp?path=gga01100$Metabolic%20pathways&termId=550025617&source=kegg), |
| **PIR_SUPERFAMILY** | [GPI-anchored wall transfer protein 1](http://pir.georgetown.edu/cgi-bin/ipcSF?id=PIRSF017321), |
| **UP_KEYWORDS** | [Acyltransferase](http://www.uniprot.org/keywords/?query=Acyltransferase), [Complete proteome](http://www.uniprot.org/keywords/?query=Complete%20proteome), [Endoplasmic reticulum](http://www.uniprot.org/keywords/?query=Endoplasmic%20reticulum), [GPI-anchor biosynthesis](http://www.uniprot.org/keywords/?query=GPI-anchor%20biosynthesis), [Membrane](http://www.uniprot.org/keywords/?query=Membrane), [Reference proteome](http://www.uniprot.org/keywords/?query=Reference%20proteome), [Transferase](http://www.uniprot.org/keywords/?query=Transferase), [Transmembrane](http://www.uniprot.org/keywords/?query=Transmembrane), [Transmembrane helix](http://www.uniprot.org/keywords/?query=Transmembrane%20helix), |
| **PIGR** | [**polymeric immunoglobulin receptor(PIGR)**](https://david.ncifcrf.gov/geneReportFull.jsp?rowids=419848) |
| **GOTERM_BP_DIRECT** | [detection of chemical stimulus involved in sensory perception of bitter taste](http://www.ebi.ac.uk/QuickGO/GTerm?id=GO:0001580), [retina homeostasis](http://www.ebi.ac.uk/QuickGO/GTerm?id=GO:0001895), [immunoglobulin transcytosis in epithelial cells mediated by polymeric immunoglobulin receptor](http://www.ebi.ac.uk/QuickGO/GTerm?id=GO:0002415), [epidermal growth factor receptor signaling pathway](http://www.ebi.ac.uk/QuickGO/GTerm?id=GO:0007173), [receptor clustering](http://www.ebi.ac.uk/QuickGO/GTerm?id=GO:0043113), |
| **GOTERM_CC_DIRECT** | [extracellular space](http://www.ebi.ac.uk/QuickGO/GTerm?id=GO:0005615), [plasma membrane](http://www.ebi.ac.uk/QuickGO/GTerm?id=GO:0005886), [integral component of membrane](http://www.ebi.ac.uk/QuickGO/GTerm?id=GO:0016021), [receptor complex](http://www.ebi.ac.uk/QuickGO/GTerm?id=GO:0043235), [extracellular exosome](http://www.ebi.ac.uk/QuickGO/GTerm?id=GO:0070062), |
| **GOTERM_MF_DIRECT** | [polymeric immunoglobulin receptor activity](http://www.ebi.ac.uk/QuickGO/GTerm?id=GO:0001792), |
| **INTERPRO** | [Immunoglobulin subtype](http://www.ebi.ac.uk/interpro/IEntry?ac=IPR003599), [Immunoglobulin-like domain](http://www.ebi.ac.uk/interpro/IEntry?ac=IPR007110), [Immunoglobulin V-set](http://www.ebi.ac.uk/interpro/IEntry?ac=IPR013106), [Immunoglobulin-like fold](http://www.ebi.ac.uk/interpro/IEntry?ac=IPR013783), |
| **KEGG_PATHWAY** | [Intestinal immune network for IgA production](https://david.ncifcrf.gov/kegg.jsp?path=gga04672$Intestinal%20immune%20network%20for%20IgA%20production&termId=550025684&source=kegg), |
| **SMART** | [IGv](http://smart.embl.de/smart/do_annotation.pl?DOMAIN=SM00406), [IG](http://smart.embl.de/smart/do_annotation.pl?DOMAIN=SM00409), |
| **UP_KEYWORDS** | [Complete proteome](http://www.uniprot.org/keywords/?query=Complete%20proteome), [Membrane](http://www.uniprot.org/keywords/?query=Membrane), [Reference proteome](http://www.uniprot.org/keywords/?query=Reference%20proteome), [Signal](http://www.uniprot.org/keywords/?query=Signal), [Transmembrane](http://www.uniprot.org/keywords/?query=Transmembrane), [Transmembrane helix](http://www.uniprot.org/keywords/?query=Transmembrane%20helix), |
| **KCNT1** | [**potassium channel, subfamily T, member 1(KCNT1)**](https://david.ncifcrf.gov/geneReportFull.jsp?rowids=395248) |
| **GOTERM_BP_DIRECT** | [potassium ion transmembrane transport](http://www.ebi.ac.uk/QuickGO/GTerm?id=GO:0071805), |
| **GOTERM_CC_DIRECT** | [voltage-gated potassium channel complex](http://www.ebi.ac.uk/QuickGO/GTerm?id=GO:0008076), [integral component of membrane](http://www.ebi.ac.uk/QuickGO/GTerm?id=GO:0016021), |
| **GOTERM_MF_DIRECT** | [voltage-gated potassium channel activity](http://www.ebi.ac.uk/QuickGO/GTerm?id=GO:0005249), [calcium-activated potassium channel activity](http://www.ebi.ac.uk/QuickGO/GTerm?id=GO:0015269), |
| **INTERPRO** | [Potassium channel, calcium-activated, BK, alpha subunit](http://www.ebi.ac.uk/interpro/IEntry?ac=IPR003929), [Ion transport 2](http://www.ebi.ac.uk/interpro/IEntry?ac=IPR013099), [NAD(P)-binding domain](http://www.ebi.ac.uk/interpro/IEntry?ac=IPR016040), |
| **UP_KEYWORDS** | [3D-structure](http://www.uniprot.org/keywords/?query=3D-structure), [Calcium](http://www.uniprot.org/keywords/?query=Calcium), [Cell membrane](http://www.uniprot.org/keywords/?query=Cell%20membrane), [Complete proteome](http://www.uniprot.org/keywords/?query=Complete%20proteome), [Glycoprotein](http://www.uniprot.org/keywords/?query=Glycoprotein), [Ion channel](http://www.uniprot.org/keywords/?query=Ion%20channel), [Ion transport](http://www.uniprot.org/keywords/?query=Ion%20transport), [Membrane](http://www.uniprot.org/keywords/?query=Membrane), [Potassium](http://www.uniprot.org/keywords/?query=Potassium), [Potassium channel](http://www.uniprot.org/keywords/?query=Potassium%20channel), [Potassium transport](http://www.uniprot.org/keywords/?query=Potassium%20transport), [Reference proteome](http://www.uniprot.org/keywords/?query=Reference%20proteome), [Transmembrane](http://www.uniprot.org/keywords/?query=Transmembrane), [Transmembrane helix](http://www.uniprot.org/keywords/?query=Transmembrane%20helix), [Transport](http://www.uniprot.org/keywords/?query=Transport), |
| **UP_SEQ_FEATURE** | chain:Potassium channel subfamily T member 1, domain:RCK N-terminal, glycosylation site:N-linked (GlcNAc...), region of interest:Pore-forming, in membrane, topological domain:Cytoplasmic, topological domain:Extracellular, transmembrane region, |
| **PBX1** | [**pre-B-cell leukemia homeobox 1(PBX1)**](https://david.ncifcrf.gov/geneReportFull.jsp?rowids=395505) |
| **GOTERM_BP_DIRECT** | [branching involved in ureteric bud morphogenesis](http://www.ebi.ac.uk/QuickGO/GTerm?id=GO:0001658), [positive regulation of cell proliferation](http://www.ebi.ac.uk/QuickGO/GTerm?id=GO:0008284), [anterior/posterior pattern specification](http://www.ebi.ac.uk/QuickGO/GTerm?id=GO:0009952), [proximal/distal pattern formation](http://www.ebi.ac.uk/QuickGO/GTerm?id=GO:0009954), [positive regulation of G2/M transition of mitotic cell cycle](http://www.ebi.ac.uk/QuickGO/GTerm?id=GO:0010971), [regulation of ossification](http://www.ebi.ac.uk/QuickGO/GTerm?id=GO:0030278), [adrenal gland development](http://www.ebi.ac.uk/QuickGO/GTerm?id=GO:0030325), [embryonic limb morphogenesis](http://www.ebi.ac.uk/QuickGO/GTerm?id=GO:0030326), [embryonic hemopoiesis](http://www.ebi.ac.uk/QuickGO/GTerm?id=GO:0035162), [negative regulation of neuron differentiation](http://www.ebi.ac.uk/QuickGO/GTerm?id=GO:0045665), [spleen development](http://www.ebi.ac.uk/QuickGO/GTerm?id=GO:0048536), [thymus development](http://www.ebi.ac.uk/QuickGO/GTerm?id=GO:0048538), [embryonic skeletal system development](http://www.ebi.ac.uk/QuickGO/GTerm?id=GO:0048706), |
| **GOTERM_CC_DIRECT** | [nucleus](http://www.ebi.ac.uk/QuickGO/GTerm?id=GO:0005634), [transcription factor complex](http://www.ebi.ac.uk/QuickGO/GTerm?id=GO:0005667), [cytoplasm](http://www.ebi.ac.uk/QuickGO/GTerm?id=GO:0005737), |
| **GOTERM_MF_DIRECT** | [RNA polymerase II core promoter proximal region sequence-specific DNA binding](http://www.ebi.ac.uk/QuickGO/GTerm?id=GO:0000978), [transcriptional activator activity, RNA polymerase II core promoter proximal region sequence-specific binding](http://www.ebi.ac.uk/QuickGO/GTerm?id=GO:0001077), [transcription factor activity, sequence-specific DNA binding](http://www.ebi.ac.uk/QuickGO/GTerm?id=GO:0003700), [sequence-specific DNA binding](http://www.ebi.ac.uk/QuickGO/GTerm?id=GO:0043565), |
| **INTERPRO** | [Homeodomain](http://www.ebi.ac.uk/interpro/IEntry?ac=IPR001356), [PBX](http://www.ebi.ac.uk/interpro/IEntry?ac=IPR005542), [Homeodomain-like](http://www.ebi.ac.uk/interpro/IEntry?ac=IPR009057), [Homeobox, conserved site](http://www.ebi.ac.uk/interpro/IEntry?ac=IPR017970), |
| **SMART** | [HOX](http://smart.embl.de/smart/do_annotation.pl?DOMAIN=SM00389), |
| **UP_KEYWORDS** | [Complete proteome](http://www.uniprot.org/keywords/?query=Complete%20proteome), [DNA-binding](http://www.uniprot.org/keywords/?query=DNA-binding), [Homeobox](http://www.uniprot.org/keywords/?query=Homeobox), [Nucleus](http://www.uniprot.org/keywords/?query=Nucleus), [Reference proteome](http://www.uniprot.org/keywords/?query=Reference%20proteome), |
| **PRL** | [**prolactin(PRL)**](https://david.ncifcrf.gov/geneReportFull.jsp?rowids=396453) |
| **GOTERM_BP_DIRECT** | [negative regulation of luteinizing hormone secretion](http://www.ebi.ac.uk/QuickGO/GTerm?id=GO:0033685), [regulation of multicellular organism growth](http://www.ebi.ac.uk/QuickGO/GTerm?id=GO:0040014), [positive regulation of JAK-STAT cascade](http://www.ebi.ac.uk/QuickGO/GTerm?id=GO:0046427), |
| **GOTERM_CC_DIRECT** | [extracellular region](http://www.ebi.ac.uk/QuickGO/GTerm?id=GO:0005576), [extracellular space](http://www.ebi.ac.uk/QuickGO/GTerm?id=GO:0005615), |
| **GOTERM_MF_DIRECT** | [hormone activity](http://www.ebi.ac.uk/QuickGO/GTerm?id=GO:0005179), |
| **INTERPRO** | [Somatotropin hormone](http://www.ebi.ac.uk/interpro/IEntry?ac=IPR001400), [Four-helical cytokine-like, core](http://www.ebi.ac.uk/interpro/IEntry?ac=IPR009079), [Four-helical cytokine, core](http://www.ebi.ac.uk/interpro/IEntry?ac=IPR012351), [Somatotropin hormone, conserved site](http://www.ebi.ac.uk/interpro/IEntry?ac=IPR018116), |
| **KEGG_PATHWAY** | [Cytokine-cytokine receptor interaction](https://david.ncifcrf.gov/kegg.jsp?path=gga04060$Cytokine-cytokine%20receptor%20interaction&termId=550025646&source=kegg), [Neuroactive ligand-receptor interaction](https://david.ncifcrf.gov/kegg.jsp?path=gga04080$Neuroactive%20ligand-receptor%20interaction&termId=550025649&source=kegg), [Jak-STAT signaling pathway](https://david.ncifcrf.gov/kegg.jsp?path=gga04630$Jak-STAT%20signaling%20pathway&termId=550025683&source=kegg), |
| **UP_KEYWORDS** | [Complete proteome](http://www.uniprot.org/keywords/?query=Complete%20proteome), [Disulfide bond](http://www.uniprot.org/keywords/?query=Disulfide%20bond), [Hormone](http://www.uniprot.org/keywords/?query=Hormone), [Reference proteome](http://www.uniprot.org/keywords/?query=Reference%20proteome), [Secreted](http://www.uniprot.org/keywords/?query=Secreted), [Signal](http://www.uniprot.org/keywords/?query=Signal), |
| **UP_SEQ_FEATURE** | chain:Prolactin, disulfide bond, sequence conflict, signal peptide, |
| **PCBD2** | [**pterin-4 alpha-carbinolamine dehydratase/dimerization cofactor of hepatocyte nuclear factor 1 alpha (TCF1) 2(PCBD2)**](https://david.ncifcrf.gov/geneReportFull.jsp?rowids=374030) |
| **GOTERM_BP_DIRECT** | [tetrahydrobiopterin biosynthetic process](http://www.ebi.ac.uk/QuickGO/GTerm?id=GO:0006729), [positive regulation of transcription, DNA-templated](http://www.ebi.ac.uk/QuickGO/GTerm?id=GO:0045893), [protein homotetramerization](http://www.ebi.ac.uk/QuickGO/GTerm?id=GO:0051289), [protein heterooligomerization](http://www.ebi.ac.uk/QuickGO/GTerm?id=GO:0051291), |
| **GOTERM_CC_DIRECT** | [nucleus](http://www.ebi.ac.uk/QuickGO/GTerm?id=GO:0005634), [mitochondrion](http://www.ebi.ac.uk/QuickGO/GTerm?id=GO:0005739), |
| **GOTERM_MF_DIRECT** | [phenylalanine 4-monooxygenase activity](http://www.ebi.ac.uk/QuickGO/GTerm?id=GO:0004505), [4-alpha-hydroxytetrahydrobiopterin dehydratase activity](http://www.ebi.ac.uk/QuickGO/GTerm?id=GO:0008124), |
| **INTERPRO** | [Transcriptional coactivator/pterin dehydratase](http://www.ebi.ac.uk/interpro/IEntry?ac=IPR001533), |
| **UP_KEYWORDS** | [Complete proteome](http://www.uniprot.org/keywords/?query=Complete%20proteome), [Lyase](http://www.uniprot.org/keywords/?query=Lyase), [Reference proteome](http://www.uniprot.org/keywords/?query=Reference%20proteome), [Tetrahydrobiopterin biosynthesis](http://www.uniprot.org/keywords/?query=Tetrahydrobiopterin%20biosynthesis), |
| **UP_SEQ_FEATURE** | chain:Pterin-4-alpha-carbinolamine dehydratase 2, |
| **P2RX1** | [**purinergic receptor P2X, ligand-gated ion channel, 1(P2RX1)**](https://david.ncifcrf.gov/geneReportFull.jsp?rowids=395190) |
| **GOTERM_BP_DIRECT** | [serotonin secretion by platelet](http://www.ebi.ac.uk/QuickGO/GTerm?id=GO:0002554), [regulation of vascular smooth muscle contraction](http://www.ebi.ac.uk/QuickGO/GTerm?id=GO:0003056), [activation of cysteine-type endopeptidase activity involved in apoptotic process](http://www.ebi.ac.uk/QuickGO/GTerm?id=GO:0006919), [insemination](http://www.ebi.ac.uk/QuickGO/GTerm?id=GO:0007320), [platelet activation](http://www.ebi.ac.uk/QuickGO/GTerm?id=GO:0030168), [response to ATP](http://www.ebi.ac.uk/QuickGO/GTerm?id=GO:0033198), [positive regulation of ion transport](http://www.ebi.ac.uk/QuickGO/GTerm?id=GO:0043270), [ceramide biosynthetic process](http://www.ebi.ac.uk/QuickGO/GTerm?id=GO:0046513), [regulation of calcium ion transport](http://www.ebi.ac.uk/QuickGO/GTerm?id=GO:0051924), |
| **GOTERM_CC_DIRECT** | [integral component of nuclear inner membrane](http://www.ebi.ac.uk/QuickGO/GTerm?id=GO:0005639), [integral component of plasma membrane](http://www.ebi.ac.uk/QuickGO/GTerm?id=GO:0005887), [external side of cell outer membrane](http://www.ebi.ac.uk/QuickGO/GTerm?id=GO:0031240), [postsynaptic membrane](http://www.ebi.ac.uk/QuickGO/GTerm?id=GO:0045211), |
| **GOTERM_MF_DIRECT** | [purinergic nucleotide receptor activity](http://www.ebi.ac.uk/QuickGO/GTerm?id=GO:0001614), [extracellular ATP-gated cation channel activity](http://www.ebi.ac.uk/QuickGO/GTerm?id=GO:0004931), [ATP binding](http://www.ebi.ac.uk/QuickGO/GTerm?id=GO:0005524), |
| **INTERPRO** | [P2X purinoreceptor](http://www.ebi.ac.uk/interpro/IEntry?ac=IPR001429), [P2X1 purinoceptor](http://www.ebi.ac.uk/interpro/IEntry?ac=IPR003044), [P2X purinoreceptor extracellular domain](http://www.ebi.ac.uk/interpro/IEntry?ac=IPR027309), |
| **KEGG_PATHWAY** | [Calcium signaling pathway](https://david.ncifcrf.gov/kegg.jsp?path=gga04020$Calcium%20signaling%20pathway&termId=550025645&source=kegg), [Neuroactive ligand-receptor interaction](https://david.ncifcrf.gov/kegg.jsp?path=gga04080$Neuroactive%20ligand-receptor%20interaction&termId=550025649&source=kegg), |
| **PIR_SUPERFAMILY** | [ATP-gated ion channel P2X4 receptor](http://pir.georgetown.edu/cgi-bin/ipcSF?id=PIRSF005713), |
| **UP_KEYWORDS** | [Complete proteome](http://www.uniprot.org/keywords/?query=Complete%20proteome), [Ion channel](http://www.uniprot.org/keywords/?query=Ion%20channel), [Ion transport](http://www.uniprot.org/keywords/?query=Ion%20transport), [Membrane](http://www.uniprot.org/keywords/?query=Membrane), [Receptor](http://www.uniprot.org/keywords/?query=Receptor), [Reference proteome](http://www.uniprot.org/keywords/?query=Reference%20proteome), [Transmembrane](http://www.uniprot.org/keywords/?query=Transmembrane), [Transmembrane helix](http://www.uniprot.org/keywords/?query=Transmembrane%20helix), [Transport](http://www.uniprot.org/keywords/?query=Transport), |
| **RABEP1** | [**rabaptin, RAB GTPase binding effector protein 1(RABEP1)**](https://david.ncifcrf.gov/geneReportFull.jsp?rowids=395930) |
| **GOTERM_BP_DIRECT** | [endocytosis](http://www.ebi.ac.uk/QuickGO/GTerm?id=GO:0006897), |
| **GOTERM_CC_DIRECT** | [endosome](http://www.ebi.ac.uk/QuickGO/GTerm?id=GO:0005768), [endocytic vesicle](http://www.ebi.ac.uk/QuickGO/GTerm?id=GO:0030139), |
| **GOTERM_MF_DIRECT** | [GTPase activator activity](http://www.ebi.ac.uk/QuickGO/GTerm?id=GO:0005096), |
| **INTERPRO** | [Rabaptin](http://www.ebi.ac.uk/interpro/IEntry?ac=IPR003914), [Rabaptin, GTPase-Rab5 binding domain](http://www.ebi.ac.uk/interpro/IEntry?ac=IPR015390), [Rabaptin coiled-coil domain](http://www.ebi.ac.uk/interpro/IEntry?ac=IPR018514), |
| **KEGG_PATHWAY** | [Endocytosis](https://david.ncifcrf.gov/kegg.jsp?path=gga04144$Endocytosis&termId=550025659&source=kegg), |
| **UP_KEYWORDS** | [Coiled coil](http://www.uniprot.org/keywords/?query=Coiled%20coil), [Complete proteome](http://www.uniprot.org/keywords/?query=Complete%20proteome), [Reference proteome](http://www.uniprot.org/keywords/?query=Reference%20proteome), |
| **RXRG** | [**retinoid X receptor, gamma(RXRG)**](https://david.ncifcrf.gov/geneReportFull.jsp?rowids=396231) |
| **GOTERM_BP_DIRECT** | [transcription, DNA-templated](http://www.ebi.ac.uk/QuickGO/GTerm?id=GO:0006351), [regulation of transcription, DNA-templated](http://www.ebi.ac.uk/QuickGO/GTerm?id=GO:0006355), [steroid hormone mediated signaling pathway](http://www.ebi.ac.uk/QuickGO/GTerm?id=GO:0043401), [protein homotetramerization](http://www.ebi.ac.uk/QuickGO/GTerm?id=GO:0051289), [positive regulation of transcription from RNA polymerase II promoter involved in cellular response to chemical stimulus](http://www.ebi.ac.uk/QuickGO/GTerm?id=GO:1901522), |
| **GOTERM_CC_DIRECT** | [cell](http://www.ebi.ac.uk/QuickGO/GTerm?id=GO:0005623), [nucleus](http://www.ebi.ac.uk/QuickGO/GTerm?id=GO:0005634), [transcription factor complex](http://www.ebi.ac.uk/QuickGO/GTerm?id=GO:0005667), |
| **GOTERM_MF_DIRECT** | [RNA polymerase II regulatory region sequence-specific DNA binding](http://www.ebi.ac.uk/QuickGO/GTerm?id=GO:0000977), [enhancer sequence-specific DNA binding](http://www.ebi.ac.uk/QuickGO/GTerm?id=GO:0001158), [transcription factor activity, sequence-specific DNA binding](http://www.ebi.ac.uk/QuickGO/GTerm?id=GO:0003700), [steroid hormone receptor activity](http://www.ebi.ac.uk/QuickGO/GTerm?id=GO:0003707), [9-cis retinoic acid receptor activity](http://www.ebi.ac.uk/QuickGO/GTerm?id=GO:0004886), [zinc ion binding](http://www.ebi.ac.uk/QuickGO/GTerm?id=GO:0008270), |
| **INTERPRO** | [Retinoid X receptor/HNF4](http://www.ebi.ac.uk/interpro/IEntry?ac=IPR000003), [Nuclear hormone receptor, ligand-binding, core](http://www.ebi.ac.uk/interpro/IEntry?ac=IPR000536), [Zinc finger, nuclear hormone receptor-type](http://www.ebi.ac.uk/interpro/IEntry?ac=IPR001628), [Steroid hormone receptor](http://www.ebi.ac.uk/interpro/IEntry?ac=IPR001723), [Zinc finger, NHR/GATA-type](http://www.ebi.ac.uk/interpro/IEntry?ac=IPR013088), [Nuclear/hormone receptor activator site AF-1](http://www.ebi.ac.uk/interpro/IEntry?ac=IPR021780), |
| **KEGG_PATHWAY** | [PPAR signaling pathway](https://david.ncifcrf.gov/kegg.jsp?path=gga03320$PPAR%20signaling%20pathway&termId=550025636&source=kegg), [Adipocytokine signaling pathway](https://david.ncifcrf.gov/kegg.jsp?path=gga04920$Adipocytokine%20signaling%20pathway&termId=550025691&source=kegg), |
| **SMART** | [ZnF_C4](http://smart.embl.de/smart/do_annotation.pl?DOMAIN=SM00399), [HOLI](http://smart.embl.de/smart/do_annotation.pl?DOMAIN=SM00430), |
| **UP_KEYWORDS** | [Alternative splicing](http://www.uniprot.org/keywords/?query=Alternative%20splicing), [Complete proteome](http://www.uniprot.org/keywords/?query=Complete%20proteome), [DNA-binding](http://www.uniprot.org/keywords/?query=DNA-binding), [Metal-binding](http://www.uniprot.org/keywords/?query=Metal-binding), [Nucleus](http://www.uniprot.org/keywords/?query=Nucleus), [Receptor](http://www.uniprot.org/keywords/?query=Receptor), [Reference proteome](http://www.uniprot.org/keywords/?query=Reference%20proteome), [Transcription](http://www.uniprot.org/keywords/?query=Transcription), [Transcription regulation](http://www.uniprot.org/keywords/?query=Transcription%20regulation), [Zinc](http://www.uniprot.org/keywords/?query=Zinc), [Zinc-finger](http://www.uniprot.org/keywords/?query=Zinc-finger), |
| **UP_SEQ_FEATURE** | chain:Retinoic acid receptor RXR-gamma, DNA-binding region:Nuclear receptor, region of interest:Hinge, region of interest:Ligand-binding, region of interest:Modulating, splice variant, zinc finger region:NR C4-type, |
| **SDR42E1** | [**short chain dehydrogenase/reductase family 42E, member 1(SDR42E1)**](https://david.ncifcrf.gov/geneReportFull.jsp?rowids=415806) |
| **COG_ONTOLOGY** | [Cell envelope biogenesis, outer membrane / Carbohydrate transport and metabolism](http://www.ncbi.nlm.nih.gov/COG/new/), |
| **GOTERM_BP_DIRECT** | [steroid biosynthetic process](http://www.ebi.ac.uk/QuickGO/GTerm?id=GO:0006694), |
| **GOTERM_CC_DIRECT** | [integral component of membrane](http://www.ebi.ac.uk/QuickGO/GTerm?id=GO:0016021), |
| **GOTERM_MF_DIRECT** | [3-beta-hydroxy-delta5-steroid dehydrogenase activity](http://www.ebi.ac.uk/QuickGO/GTerm?id=GO:0003854), |
| **INTERPRO** | [3-beta hydroxysteroid dehydrogenase/isomerase](http://www.ebi.ac.uk/interpro/IEntry?ac=IPR002225), [NAD(P)-binding domain](http://www.ebi.ac.uk/interpro/IEntry?ac=IPR016040), |
| **UP_KEYWORDS** | [Complete proteome](http://www.uniprot.org/keywords/?query=Complete%20proteome), [Membrane](http://www.uniprot.org/keywords/?query=Membrane), [Oxidoreductase](http://www.uniprot.org/keywords/?query=Oxidoreductase), [Reference proteome](http://www.uniprot.org/keywords/?query=Reference%20proteome), [Transmembrane](http://www.uniprot.org/keywords/?query=Transmembrane), [Transmembrane helix](http://www.uniprot.org/keywords/?query=Transmembrane%20helix), |
| **SLC7A10** | [**solute carrier family 7, (neutral amino acid transporter, y+ system) member 10(SLC7A10)**](https://david.ncifcrf.gov/geneReportFull.jsp?rowids=415774) |
| **GOTERM_BP_DIRECT** | [L-serine transport](http://www.ebi.ac.uk/QuickGO/GTerm?id=GO:0015825), [D-alanine transport](http://www.ebi.ac.uk/QuickGO/GTerm?id=GO:0042941), [D-serine transport](http://www.ebi.ac.uk/QuickGO/GTerm?id=GO:0042942), |
| **GOTERM_CC_DIRECT** | [integral component of plasma membrane](http://www.ebi.ac.uk/QuickGO/GTerm?id=GO:0005887), |
| **GOTERM_MF_DIRECT** | [L-serine transmembrane transporter activity](http://www.ebi.ac.uk/QuickGO/GTerm?id=GO:0015194), [antiporter activity](http://www.ebi.ac.uk/QuickGO/GTerm?id=GO:0015297), |
| **INTERPRO** | [Amino acid/polyamine transporter I](http://www.ebi.ac.uk/interpro/IEntry?ac=IPR002293), |
| **PIR_SUPERFAMILY** | [amino acid transporter](http://pir.georgetown.edu/cgi-bin/ipcSF?id=PIRSF006060), |
| **UP_KEYWORDS** | [Complete proteome](http://www.uniprot.org/keywords/?query=Complete%20proteome), [Membrane](http://www.uniprot.org/keywords/?query=Membrane), [Reference proteome](http://www.uniprot.org/keywords/?query=Reference%20proteome), [Transmembrane](http://www.uniprot.org/keywords/?query=Transmembrane), [Transmembrane helix](http://www.uniprot.org/keywords/?query=Transmembrane%20helix), |
| **SPNS3** | [**spinster homolog 3 (Drosophila)(SPNS3)**](https://david.ncifcrf.gov/geneReportFull.jsp?rowids=417493) |
| **GOTERM_BP_DIRECT** | [sphingosine-1-phosphate signaling pathway](http://www.ebi.ac.uk/QuickGO/GTerm?id=GO:0003376), [locomotion](http://www.ebi.ac.uk/QuickGO/GTerm?id=GO:0040011), [transmembrane transport](http://www.ebi.ac.uk/QuickGO/GTerm?id=GO:0055085), |
| **GOTERM_CC_DIRECT** | [lysosomal membrane](http://www.ebi.ac.uk/QuickGO/GTerm?id=GO:0005765), [integral component of membrane](http://www.ebi.ac.uk/QuickGO/GTerm?id=GO:0016021), [vesicle](http://www.ebi.ac.uk/QuickGO/GTerm?id=GO:0031982), |
| **GOTERM_MF_DIRECT** | [sphingolipid transporter activity](http://www.ebi.ac.uk/QuickGO/GTerm?id=GO:0046624), |
| **INTERPRO** | [Major facilitator superfamily](http://www.ebi.ac.uk/interpro/IEntry?ac=IPR011701), [Major facilitator superfamily domain](http://www.ebi.ac.uk/interpro/IEntry?ac=IPR020846), |
| **UP_KEYWORDS** | [Complete proteome](http://www.uniprot.org/keywords/?query=Complete%20proteome), [Membrane](http://www.uniprot.org/keywords/?query=Membrane), [Reference proteome](http://www.uniprot.org/keywords/?query=Reference%20proteome), [Transmembrane](http://www.uniprot.org/keywords/?query=Transmembrane), [Transmembrane helix](http://www.uniprot.org/keywords/?query=Transmembrane%20helix), |
| **SULT1B** | [**sulfotransferase family, cytosolic, 1B(SULT1B)**](https://david.ncifcrf.gov/geneReportFull.jsp?rowids=422661) |
| **GOTERM_BP_DIRECT** | [xenobiotic metabolic process](http://www.ebi.ac.uk/QuickGO/GTerm?id=GO:0006805), [thyroid hormone metabolic process](http://www.ebi.ac.uk/QuickGO/GTerm?id=GO:0042403), [sulfation](http://www.ebi.ac.uk/QuickGO/GTerm?id=GO:0051923), |
| **GOTERM_MF_DIRECT** | [sulfotransferase activity](http://www.ebi.ac.uk/QuickGO/GTerm?id=GO:0008146), |
| **INTERPRO** | [Sulfotransferase domain](http://www.ebi.ac.uk/interpro/IEntry?ac=IPR000863), [P-loop containing nucleoside triphosphate hydrolase](http://www.ebi.ac.uk/interpro/IEntry?ac=IPR027417), |
| **UP_KEYWORDS** | [Complete proteome](http://www.uniprot.org/keywords/?query=Complete%20proteome), [Reference proteome](http://www.uniprot.org/keywords/?query=Reference%20proteome), [Transferase](http://www.uniprot.org/keywords/?query=Transferase), |
| **SULT1B1** | [**sulfotransferase family, cytosolic, 1B, member 1(SULT1B1)**](https://david.ncifcrf.gov/geneReportFull.jsp?rowids=395227) |
| **GOTERM_BP_DIRECT** | [xenobiotic metabolic process](http://www.ebi.ac.uk/QuickGO/GTerm?id=GO:0006805), [steroid metabolic process](http://www.ebi.ac.uk/QuickGO/GTerm?id=GO:0008202), [thyroid hormone metabolic process](http://www.ebi.ac.uk/QuickGO/GTerm?id=GO:0042403), [sulfation](http://www.ebi.ac.uk/QuickGO/GTerm?id=GO:0051923), |
| **GOTERM_CC_DIRECT** | [cytoplasm](http://www.ebi.ac.uk/QuickGO/GTerm?id=GO:0005737), |
| **GOTERM_MF_DIRECT** | [sulfotransferase activity](http://www.ebi.ac.uk/QuickGO/GTerm?id=GO:0008146), |
| **INTERPRO** | [Sulfotransferase domain](http://www.ebi.ac.uk/interpro/IEntry?ac=IPR000863), [P-loop containing nucleoside triphosphate hydrolase](http://www.ebi.ac.uk/interpro/IEntry?ac=IPR027417), |
| **UP_KEYWORDS** | [Complete proteome](http://www.uniprot.org/keywords/?query=Complete%20proteome), [Cytoplasm](http://www.uniprot.org/keywords/?query=Cytoplasm), [Lipid metabolism](http://www.uniprot.org/keywords/?query=Lipid%20metabolism), [Reference proteome](http://www.uniprot.org/keywords/?query=Reference%20proteome), [Steroid metabolism](http://www.uniprot.org/keywords/?query=Steroid%20metabolism), [Transferase](http://www.uniprot.org/keywords/?query=Transferase), |
| **UP_SEQ_FEATURE** | active site:Proton acceptor, binding site:PAPS, chain:Sulfotransferase family cytosolic 1B member 1, nucleotide phosphate-binding region:PAPS, |
| **TXNDC15** | [**thioredoxin domain containing 15(TXNDC15)**](https://david.ncifcrf.gov/geneReportFull.jsp?rowids=416310) |
| **GOTERM_BP_DIRECT** | [cell redox homeostasis](http://www.ebi.ac.uk/QuickGO/GTerm?id=GO:0045454), |
| **GOTERM_CC_DIRECT** | [cell](http://www.ebi.ac.uk/QuickGO/GTerm?id=GO:0005623), [integral component of membrane](http://www.ebi.ac.uk/QuickGO/GTerm?id=GO:0016021), |
| **INTERPRO** | [Thioredoxin-like fold](http://www.ebi.ac.uk/interpro/IEntry?ac=IPR012336), [Thioredoxin domain](http://www.ebi.ac.uk/interpro/IEntry?ac=IPR013766), |
| **UP_KEYWORDS** | [Complete proteome](http://www.uniprot.org/keywords/?query=Complete%20proteome), [Membrane](http://www.uniprot.org/keywords/?query=Membrane), [Reference proteome](http://www.uniprot.org/keywords/?query=Reference%20proteome), [Signal](http://www.uniprot.org/keywords/?query=Signal), [Transmembrane](http://www.uniprot.org/keywords/?query=Transmembrane), [Transmembrane helix](http://www.uniprot.org/keywords/?query=Transmembrane%20helix), |
| **TMPRSS11F** | [**transmembrane protease, serine 11F(TMPRSS11F)**](https://david.ncifcrf.gov/geneReportFull.jsp?rowids=428763) |
| **GOTERM_CC_DIRECT** | [extracellular region](http://www.ebi.ac.uk/QuickGO/GTerm?id=GO:0005576), [integral component of plasma membrane](http://www.ebi.ac.uk/QuickGO/GTerm?id=GO:0005887), |
| **GOTERM_MF_DIRECT** | [serine-type endopeptidase activity](http://www.ebi.ac.uk/QuickGO/GTerm?id=GO:0004252), |
| **INTERPRO** | [SEA domain](http://www.ebi.ac.uk/interpro/IEntry?ac=IPR000082), [Peptidase S1](http://www.ebi.ac.uk/interpro/IEntry?ac=IPR001254), [Peptidase S1A, chymotrypsin-type](http://www.ebi.ac.uk/interpro/IEntry?ac=IPR001314), [Trypsin-like cysteine/serine peptidase domain](http://www.ebi.ac.uk/interpro/IEntry?ac=IPR009003), [Peptidase S1A, HAT/DESC1](http://www.ebi.ac.uk/interpro/IEntry?ac=IPR017329), [Peptidase S1, trypsin family, active site](http://www.ebi.ac.uk/interpro/IEntry?ac=IPR018114), |
| **PIR_SUPERFAMILY** | [transmembrane serine protease, TMPRSS11A type](http://pir.georgetown.edu/cgi-bin/ipcSF?id=PIRSF037941), |
| **SMART** | [Tryp_SPc](http://smart.embl.de/smart/do_annotation.pl?DOMAIN=SM00020), |
| **UP_KEYWORDS** | [Complete proteome](http://www.uniprot.org/keywords/?query=Complete%20proteome), [Disulfide bond](http://www.uniprot.org/keywords/?query=Disulfide%20bond), [Hydrolase](http://www.uniprot.org/keywords/?query=Hydrolase), [Membrane](http://www.uniprot.org/keywords/?query=Membrane), [Protease](http://www.uniprot.org/keywords/?query=Protease), [Reference proteome](http://www.uniprot.org/keywords/?query=Reference%20proteome), [Serine protease](http://www.uniprot.org/keywords/?query=Serine%20protease), [Transmembrane](http://www.uniprot.org/keywords/?query=Transmembrane), [Transmembrane helix](http://www.uniprot.org/keywords/?query=Transmembrane%20helix), |
| **TMEM121** | [**transmembrane protein 121(TMEM121)**](https://david.ncifcrf.gov/geneReportFull.jsp?rowids=395216) |
| **GOTERM_CC_DIRECT** | [integral component of membrane](http://www.ebi.ac.uk/QuickGO/GTerm?id=GO:0016021), |
| **UP_KEYWORDS** | [Complete proteome](http://www.uniprot.org/keywords/?query=Complete%20proteome), [Membrane](http://www.uniprot.org/keywords/?query=Membrane), [Reference proteome](http://www.uniprot.org/keywords/?query=Reference%20proteome), [Transmembrane](http://www.uniprot.org/keywords/?query=Transmembrane), [Transmembrane helix](http://www.uniprot.org/keywords/?query=Transmembrane%20helix), |
| **UP_SEQ_FEATURE** | chain:Transmembrane protein 121, transmembrane region, |
| **ZFHX4** | [**zinc finger homeobox 4(ZFHX4)**](https://david.ncifcrf.gov/geneReportFull.jsp?rowids=395904) |
| **COG_ONTOLOGY** | [Transcription](http://www.ncbi.nlm.nih.gov/COG/new/), |
| **GOTERM_BP_DIRECT** | [transcription, DNA-templated](http://www.ebi.ac.uk/QuickGO/GTerm?id=GO:0006351), [regulation of transcription, DNA-templated](http://www.ebi.ac.uk/QuickGO/GTerm?id=GO:0006355), |
| **GOTERM_CC_DIRECT** | [nucleus](http://www.ebi.ac.uk/QuickGO/GTerm?id=GO:0005634), |
| **GOTERM_MF_DIRECT** | [zinc ion binding](http://www.ebi.ac.uk/QuickGO/GTerm?id=GO:0008270), [sequence-specific DNA binding](http://www.ebi.ac.uk/QuickGO/GTerm?id=GO:0043565), |
| **INTERPRO** | [Homeodomain](http://www.ebi.ac.uk/interpro/IEntry?ac=IPR001356), [Zinc finger, U1-type](http://www.ebi.ac.uk/interpro/IEntry?ac=IPR003604), [Zinc finger, C2H2](http://www.ebi.ac.uk/interpro/IEntry?ac=IPR007087), [Homeodomain-like](http://www.ebi.ac.uk/interpro/IEntry?ac=IPR009057), [Zinc finger C2H2-type/integrase DNA-binding domain](http://www.ebi.ac.uk/interpro/IEntry?ac=IPR013087), [Zinc finger, C2H2-like](http://www.ebi.ac.uk/interpro/IEntry?ac=IPR015880), [Homeobox, conserved site](http://www.ebi.ac.uk/interpro/IEntry?ac=IPR017970), |
| **SMART** | [ZnF_C2H2](http://smart.embl.de/smart/do_annotation.pl?DOMAIN=SM00355), [HOX](http://smart.embl.de/smart/do_annotation.pl?DOMAIN=SM00389), [ZnF_U1](http://smart.embl.de/smart/do_annotation.pl?DOMAIN=SM00451), |
| **UP_KEYWORDS** | [Coiled coil](http://www.uniprot.org/keywords/?query=Coiled%20coil), [Complete proteome](http://www.uniprot.org/keywords/?query=Complete%20proteome), [DNA-binding](http://www.uniprot.org/keywords/?query=DNA-binding), [Homeobox](http://www.uniprot.org/keywords/?query=Homeobox), [Metal-binding](http://www.uniprot.org/keywords/?query=Metal-binding), [Nucleus](http://www.uniprot.org/keywords/?query=Nucleus), [Reference proteome](http://www.uniprot.org/keywords/?query=Reference%20proteome), [Repeat](http://www.uniprot.org/keywords/?query=Repeat), [Repressor](http://www.uniprot.org/keywords/?query=Repressor), [Transcription](http://www.uniprot.org/keywords/?query=Transcription), [Transcription regulation](http://www.uniprot.org/keywords/?query=Transcription%20regulation), [Zinc](http://www.uniprot.org/keywords/?query=Zinc), [Zinc-finger](http://www.uniprot.org/keywords/?query=Zinc-finger), |
| **UP_SEQ_FEATURE** | chain:Zinc finger homeobox protein 4, compositionally biased region:Gln-rich, compositionally biased region:Poly-Ser, compositionally biased region:Pro-rich, compositionally biased region:Ser-rich, DNA-binding region:Homeobox 1, DNA-binding region:Homeobox 2, DNA-binding region:Homeobox 3, DNA-binding region:Homeobox 4, sequence conflict, zinc finger region:C2H2-type 1, zinc finger region:C2H2-type 10, zinc finger region:C2H2-type 11, zinc finger region:C2H2-type 12, zinc finger region:C2H2-type 13, zinc finger region:C2H2-type 14, zinc finger region:C2H2-type 15; degenerate, zinc finger region:C2H2-type 16, zinc finger region:C2H2-type 17, zinc finger region:C2H2-type 18; degenerate, zinc finger region:C2H2-type 19; degenerate, zinc finger region:C2H2-type 2, zinc finger region:C2H2-type 20, zinc finger region:C2H2-type 3, zinc finger region:C2H2-type 4; degenerate, zinc finger region:C2H2-type 5, zinc finger region:C2H2-type 6, zinc finger region:C2H2-type 7, zinc finger region:C2H2-type 8, zinc finger region:C2H2-type 9, |
| **ZBED1** | [**zinc finger, BED-type containing 1(ZBED1)**](https://david.ncifcrf.gov/geneReportFull.jsp?rowids=418663) |
| **GOTERM_BP_DIRECT** | [regulation of transcription from RNA polymerase II promoter](http://www.ebi.ac.uk/QuickGO/GTerm?id=GO:0006357), |
| **GOTERM_CC_DIRECT** | [nucleus](http://www.ebi.ac.uk/QuickGO/GTerm?id=GO:0005634), [cytoplasm](http://www.ebi.ac.uk/QuickGO/GTerm?id=GO:0005737), [actin cytoskeleton](http://www.ebi.ac.uk/QuickGO/GTerm?id=GO:0015629), |
| **GOTERM_MF_DIRECT** | [RNA polymerase II regulatory region sequence-specific DNA binding](http://www.ebi.ac.uk/QuickGO/GTerm?id=GO:0000977), [transcription factor activity, sequence-specific DNA binding](http://www.ebi.ac.uk/QuickGO/GTerm?id=GO:0003700), |
| **INTERPRO** | [Zinc finger, BED-type predicted](http://www.ebi.ac.uk/interpro/IEntry?ac=IPR003656), [HAT dimerisation](http://www.ebi.ac.uk/interpro/IEntry?ac=IPR008906), [Ribonuclease H-like domain](http://www.ebi.ac.uk/interpro/IEntry?ac=IPR012337), |
| **SMART** | [ZnF_BED](http://smart.embl.de/smart/do_annotation.pl?DOMAIN=SM00614), |
| **UP_KEYWORDS** | [Complete proteome](http://www.uniprot.org/keywords/?query=Complete%20proteome), [Reference proteome](http://www.uniprot.org/keywords/?query=Reference%20proteome), |
| **ZNHIT3** | [**zinc finger, HIT-type containing 3(ZNHIT3)**](https://david.ncifcrf.gov/geneReportFull.jsp?rowids=100859083) |
| **INTERPRO** | [Zinc finger, HIT-type](http://www.ebi.ac.uk/interpro/IEntry?ac=IPR007529), |
| **UP_KEYWORDS** | [Complete proteome](http://www.uniprot.org/keywords/?query=Complete%20proteome), [Reference proteome](http://www.uniprot.org/keywords/?query=Reference%20proteome), |
