# Supplementary material for: Genome-Wide Association Study of Growth Performance and Immune Response to Newcastle Disease Virus of Indigenous Chicken in Rwanda
Source: Front Genet. 2021 Aug 16;12:723980. doi: 10.3389/fgene.2021.723980 (PMC8570395; doi:10.3389/fgene.2021.723980)
Supplement: Supplementary file 2 [file Data_Sheet_2.docx]

Figure 2 and Figure 3 revealed the relation of normal theoretical quantiles of the probability distributions between expected (x-axis) and observed (y-axis) p-values from IC antibody response to Newcastle disease in Rwanda.


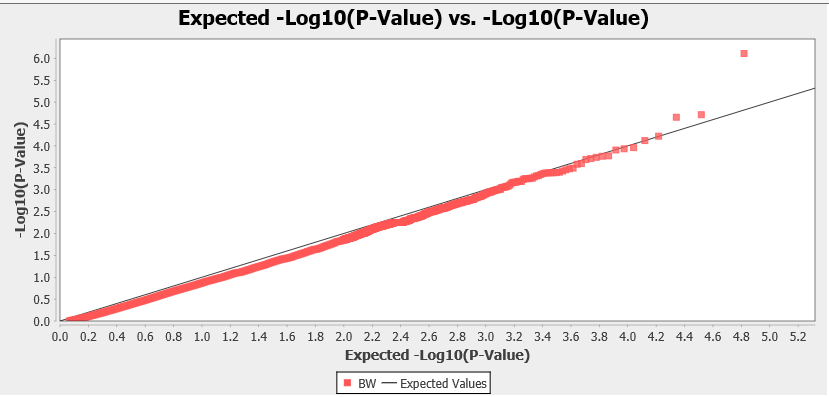


**Figure 2. QQ-plot showing the relation of normal theoretical quantiles of the probability distributions between expected (x-axis) and observed (y-axis) p-values from BW of IC in Rwanda**


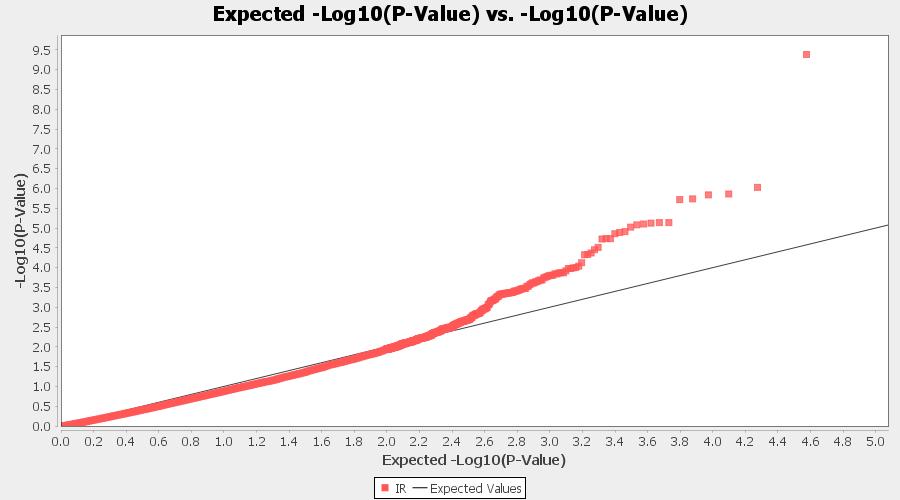


**Figure 3. QQ-plot showing the relation of normal theoretical quantiles of the probability distributions between expected (x-axis) and observed (y-axis) p-values from IC antibody response to Newcastle disease in Rwanda**
